# Anti-schistosomal activities of quinoxaline-containing compounds: From hit identification to lead optimisation

**DOI:** 10.1016/j.ejmech.2021.113823

**Published:** 2021-12-15

**Authors:** Gilda Padalino, Nelly El-Sakkary, Lawrence J. Liu, Chenxi Liu, Danielle S.G. Harte, Rachel E. Barnes, Edward Sayers, Josephine Forde-Thomas, Helen Whiteland, Marcella Bassetto, Salvatore Ferla, George Johnson, Arwyn T. Jones, Conor R. Caffrey, Iain Chalmers, Andrea Brancale, Karl F. Hoffmann

**Affiliations:** a Institute of Biological, Environmental and Rural Sciences (IBERS), Aberystwyth University, Aberystwyth, SY23 3DA, United Kingdom; b Center for Discovery and Innovation in Parasitic Diseases (CDIPD), Skaggs School of Pharmacy and Pharmaceutical Sciences, University of California, San Diego, La Jolla, CA 92093, USA; c Swansea University Medical School, Swansea University, Swansea, SA2 8PP, United Kingdom; d School of Pharmacy and Pharmaceutical Sciences, Cardiff University, Redwood Building, King Edward VII Avenue, Cardiff, CF10 3NB, United Kingdom; e Department of Chemistry, College of Science and Engineering, Swansea University, Swansea, SA2 8PP, United Kingdom

**Keywords:** Schistosomiasis, Quinoxaline, SAR, Drug discovery

## Abstract

Schistosomiasis is a neglected disease of poverty that is caused by infection with blood fluke species contained within the genus *Schistosoma*. For the last 40 years, control of schistosomiasis in endemic regions has predominantly been facilitated by administration of a single drug, praziquantel. Due to limitations in this mono-chemotherapeutic approach for sustaining schistosomiasis control into the future, alternative anti-schistosomal compounds are increasingly being sought by the drug discovery community. Herein, we describe a multi-pronged, integrated strategy that led to the identification and further exploration of the quinoxaline core as a promising anti-schistosomal scaffold.

Firstly, phenotypic screening of commercially available small molecules resulted in the identification of a moderately active hit compound against *Schistosoma mansoni* (1, EC_50_ = 4.59 μM on schistosomula). Secondary exploration of the chemical space around compound 1 led to the identification of a quinoxaline-core containing, non-genotoxic lead (compound 22). Compound 22 demonstrated substantially improved activities on both intra-mammalian (EC_50_ = 0.44 μM, 0.20 μM and 84.7 nM, on schistosomula, juvenile and adult worms, respectively) and intra-molluscan (sporocyst) *S. mansoni* lifecycle stages. Further medicinal chemistry optimisation of compound 22, resulting in the generation of 20 additional analogues, improved our understanding of the structure-activity relationship and resulted in considerable improvements in both anti-schistosome potency and selectivity (e.g. compound 30; EC_50_ = 2.59 nM on adult worms; selectivity index compared to the HepG2 cell line = 348). Some derivatives of compound 22 (e.g. 31 and 33) also demonstrated significant activity against the two other medically important species, *Schistosoma haematobium* and *Schistosoma japonicum*. Further optimisation of this class of anti-schistosomal is ongoing and could lead to the development of an urgently needed alternative to praziquantel for assisting in schistosomiasis elimination strategies.

## Highlights

• Lead compound 22 was identified with EC_50_ of 0.44 µM and 84.7 nM for schistosomula and adult worms.
• 20 analogues of the lead compound 22 were synthesised.
• Compounds 25, 30 and 32 showed the best selectivity profile.
• Compounds 31 and 33 are the most active on three medically important schistosome species.

## Graphical abstract

**Figure:**
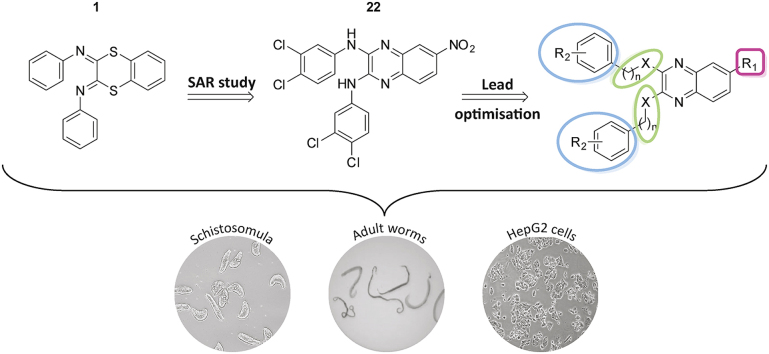

## 1 Introduction

The neglected tropical disease (NTD) schistosomiasis is the second most debilitating human parasitic disease after malaria [1,2]. It is currently listed in the World Health Organisation's road map for ‘*Ending the Neglect to Attain the Sustainable Development Goals*’ with an ambition to eliminate schistosomiasis as a public health problem in 78 endemic countries by 2030 [3]. In the absence of a clinically-approved vaccine, the complete (over) reliance on a single chemotherapy (praziquantel, PZQ) to treat schistosomiasis represents a serious challenge in reaching this ambitious objective.

Therefore, there is an urgent need to identify novel anti-schistosomals as an alternative to PZQ, should drug insensitive schistosomes develop [4], or to be used in combination with PZQ, to improve upon some of PZQ's limitations [5,6] (e.g. ineffectiveness against juvenile stage schistosomes [7]). Towards this goal, several drug discovery strategies have been applied in the pursuit of identifying novel chemotherapeutic agents and include (e.g.) drug repositioning [[8], [9], [10]], ‘piggybacking’ approaches [[11], [12], [13]], machine learning [14] and the *de novo* design of compounds using either ligand- or target-based molecular modelling approaches [[15], [16], [17], [18], [19]].

Regardless of the strategy used to initiate drug discovery for schistosomiasis, next-generation anti-schistosomals should be prioritised for their effectiveness in killing schistosome lifecycle stages found in the human host (e.g. schistosomula, juvenile worms and adult worms) as well as their ability to inhibit the production of eggs, which drives both pathogenesis and transmission of schistosomiasis [20]. Compounds should also demonstrate broad anti-schistosomal activity against human-infective species endemic to both New (e.g. South America) and Old (e.g. Africa) Worlds (*Schistosoma mansoni*) [21], species responsible for most pathology as well as additionally linked to bladder cancer (*Schistosoma haematobium*) [22,23] and species predominantly responsible for schistosomiasis in Asia (*Schistosoma japonicum*) [24]. Finally, anti-schistosomal compounds should show a greater selectivity for the parasite (when compared to the definitive host) and not display overt genotoxicity. While other criteria (e.g. cost of goods, synthesis steps, compound stability/shelf life, oral delivery, safe in children/pregnant women/breast-feeding mothers) are being actively discussed within the schistosomiasis drug-discovery community, the considerations listed above are often used as go/no-go decision points in early-stage hit discovery and hit to lead optimisation projects.

In this study, as part of our ongoing small-molecule screening activities, we present the discovery of a novel anti-schistosomal hit compound (1) that led to the identification of a more active, quinoxaline-containing compound 22. As this compound additionally demonstrated favourable cytotoxicity and genotoxicity characteristics, optimisation around the central quinoxaline scaffold was performed resulting in analogues displaying substantial improvements in both anti-parasitic activity and selectivity. Progression of these leads represents a promising starting point for the development of urgently needed, next-generation anti-schistosomals.

## 2 Results and discussion

### 2.1 Primary screen and hit compound identification

**Fig. 1:**
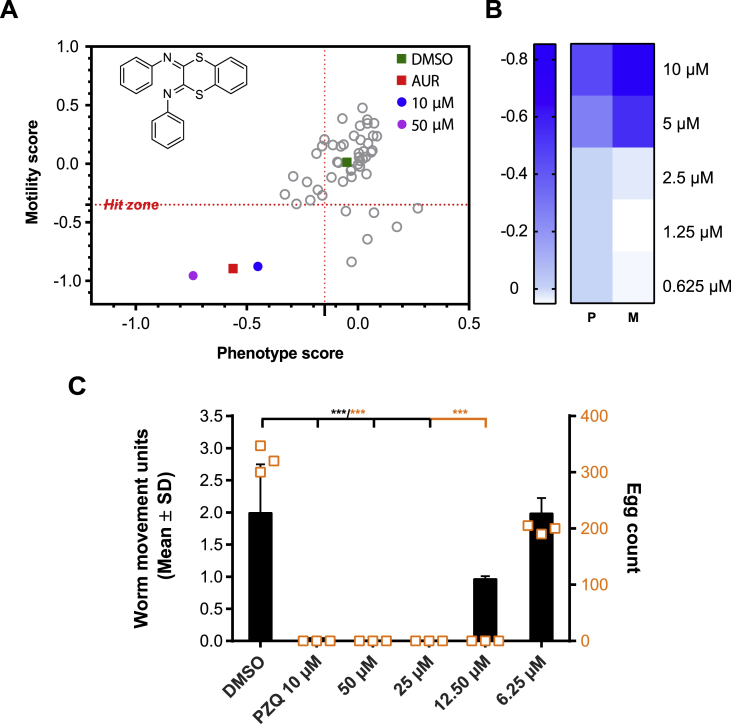
Anti-schistosomal activities of compound 1. (A) - Mechanically-transformed schistosomula were incubated for 72 h with a pre-plated library of small molecules (the Specs pre-plated fragment-based library). Here is shown only a subset of 80 small molecules of this library relevant to this study. Each compound was tested at 10 and 50 μM (each of them in duplicate). One of the four representative screens (primary screens) is shown here (barcode 0255, Z′ scores are reported in Table S1). Compounds with activity on both schistosomula phenotype and motility are shown within the ‘Hit Zone’ (delineated by the dotted red lines, defining - 0.15 and - 0.35 as threshold anti-schistosomula values for phenotype and motility scores, respectively). The chemical structure of compound 1 is shown here. The grey circles represent the remaining compounds not displaying any significant activity. (B) - Compound 1 was screened against mechanically-transformed schistosomula during four repeat dose-response titrations (0.625, 1.25, 2.5, 5 and 10 μM) with average phenotype (P) and motility (M) scores reported as a heat map. The range of scores is shown as a gradient: dark blue indicates the most positive effect of the compound on either phenotype or motility, light blue represents progressively reduced compound efficacy and white represents minimal/no effect on either phenotype or motility. Z′ scores for these four repeat titrations are reported in Table S1. (C) - A dose response titration (50–6.25 μM) of compound 1 was additionally performed on adult *S. mansoni* worms. After 72 h, worm motility (black bars) and egg production (orange squares) was quantified. Each titration was performed in three independent screens. A Kruskal-Wallis ANOVA followed by Dunn's multiple comparisons test was performed (comparing treatments to DMSO control). ∗∗∗ represents *p* < 0.0002.

During phenotypic screening of a pre-plated small molecule library (the Specs pre-plated fragment-based library, provided as 96 deep well plates with 80 compounds each [25]), compound 1 was identified as a hit at both 50 and 10 μM on *S. mansoni* schistosomula (Fig. 1A and Fig. S1) after 72 h of co-incubation (number of replicates performed and Z′ scores reported in Table S1). Further dose-response titration on this intra-mammalian larval stage (Fig. 1B) showed that this compound retained activity on the parasite down to 5 μM (EC_50_ of 4.72 and 4.47 μM for phenotype and motility, respectively; Z′ scores reported in Table S1). Translation of compound 1's anti-schistosomula activity on adult male and female worm pairs was subsequently investigated (Fig. 1C). At the highest concentrations tested (50 and 25 μM), compound 1 had a lethal effect on adults (absence of body/gut movement, plate detachment and severe damage to the tegument). At lower compound concentrations, worm motility began to recover with individuals displaying normal motility at 6.25 μM (i.e. no different than worms co-cultured in media containing only the DMSO solvent). Compound 1 also significantly inhibited the production of *in vitro* laid eggs (IVLEs) up until 12.5 μM; reductions in IVLEs were also found at the lowest concentration tested (6.25 μM), although not significant.

### 2.2 Secondary screens and prioritisation of lead compound for follow-up studies

Aiming to characterise related compounds with increased anti-schistosomal potencies, additional small molecules were sought for secondary screening based on their structural similarity to compound 1 (similarity threshold 50%) and commercial availability (from Specs, availability >0 mg at the time this study was performed). Structural diversity of these compounds was focused broadly around three regions of compound 1: the central condensed scaffold, functionalisation of the C6 position and substituents on the *N*-aromatic ring (Fig. S2). As a result, an additional 23 small molecules (Table S2), containing the commercially-available, structurally-related modifications were identified and purchased from the Specs library (from which compound 1 was originally obtained for the primary screen).

**Fig. 2:**
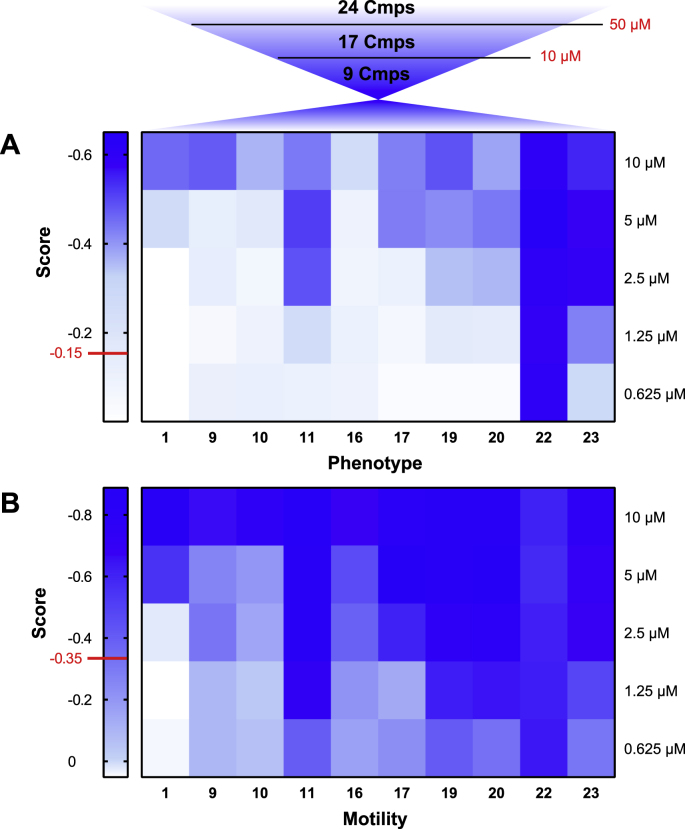
Compound 1 related analogues demonstrate variable anti-schistosomula activities. A total of 24 compounds (hit compound 1 and 23 structural analogues) were tested on schistosomula for 72 h at 50 μM resulting in the identification of 17 hits, 9 of which retained activity at 10 μM. These 9 compounds were further titrated (for 72 h) on schistosomula (0.625, 1.25, 2.5, 5 and 10 μM). Two independent dose response titrations were performed, and each compound concentration was evaluated in duplicate (Z′ scores for each screen are reported in Table S3). The mean average on phenotype and motility is here represented as heat maps (shades defined in Fig. 1) in panels (A) and (B), respectively. Scores of - 0.15 and - 0.35 are defined as threshold anti-schistosomula values for phenotype and motility, respectively (shown in red on the heat map).

An initial evaluation of these compounds (in addition to compound 1) was performed on the schistosomula stage at 10 and 50 μM (replicate primary screens for compound/concentration point and Z′ scores are reported in Table S3). A total of 17/24 compounds were hits at 50 μM; however, only 9 compounds (9, 10, 11, 16, 17, 19, 20, 22 and 23; Table 1) retained activity at 10 μM. Based on these findings, the 9 hit compounds (at 10 μM) were selected for further titrations (Fig. 2; Z′ scores of these dose-response titration screens reported in Table S3). Among the compounds screened, three (9, 10 and 16; Table 1) showed no improvement in anti-schistosomal activity compared to the initial hit compound (1). Analysis of the dose-response curves of the remaining 6 compounds (compounds 11, 17, 19, 20, 22 and 23; Table 1) highlighted a substantial improvement in potency over compound 1 on both phenotype and motility metrics (Fig. 2A and B, respectively).

**Table 1:** Anti-schistosomula activities of compounds 1–24.

	Activity on schistosomula (EC50, μM - 72 h)			
Compound	Phenotype	Motility		Average
1	4.72 (4.04–5.19)	4.47 (3.98–5.45)		4.59
2	>50			ND
3	>50			ND
4	>50			ND
5	>10			ND
6	>10			ND
7	>10			ND
8	>50			ND
9	5.92 (4.37–7.47)	4.87 (3.38–6.79)		5.39
10	5.12 (2.82–7.42)	5.83 (4.40–7.26)		5.47
11	1.38 (1.12–1.78)	1.12 (0.99–1.25)		1.25
12	>10			ND
13	>10			ND
14	>50			ND
15	>10			ND
16	5.42 (3.27–7.57)	3.69 (1.69–5.69)		4.55
17	2.88 (2.32–3.44)	2.45 (0.65–4.25)		2.66
18	>10			ND
19	1.89 (1.57–2.28)	1.35 (1.06–1.76)		1.62
20	1.62 (1.36–1.95)	1.23 (1.01–1.54)		1.42
21	>50			ND
22	0.40 (0.25–0.55)	0.38 (0.11–0.49)		0.39
23	1.23 (0.27–2.19)	1.44 (0.54–2.34)		1.33
24	>10			ND
AUR	0.37 (0.36–0.39)		0.45 (0.42–0.47)	0.41
PZQ	1.14 (1.10–1.17)		0.88 (0.79–0.97)	1.01

Schistosomula EC_50_ (phenotype and motility metrics as well as the mean of these two metrics) values of compound 1 and its 23 derivatives were calculated based on two dose response titrations (0.313–10 μM). Similarly, EC_50_ values were calculated for Auranofin (AUR) and praziquantel (PZQ), included here for comparison. Where no effect was seen on schistosomula at the primary tested concentrations (10 and 50 μM), the EC_50_ was said to be higher than 50 μM. The EC_50_ was said to be higher than 10 μM if the compound was defined as a hit at 50 μM, but not at 10 μM. All the data were analysed using GraphPad Prism. ND: Not Determined (where titration was not performed so EC_50_ value was not available and arithmetic average was not calculated). The 95% CI (Confidence Interval) values are reported in brackets.

**Fig. 3:**
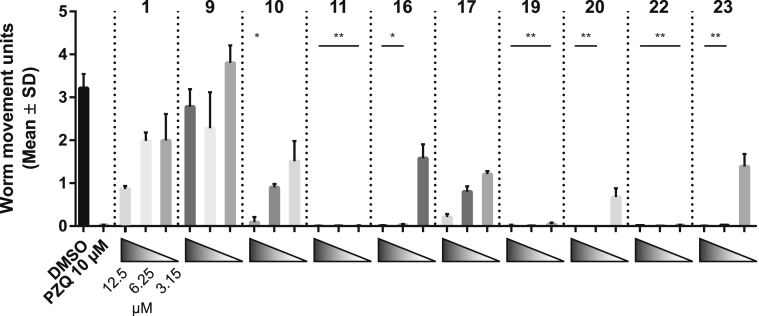
Dose response titration of compound 1-related analogues on adult *S. mansoni* worms. The 9 compounds (identified as hits at 10 μM on schistosomula) were screened on adult worms at 12.5 μM (sub-lethal concentration for 1, here reported as the reference) and two lower concentrations (3.15 and 6.25 μM). Each titration was performed in duplicate across two independent screens. The effect on schistosome motility was quantified using WormassayGP2. The bar chart shows the average worm movements recorded during the two independent screens, in comparison to the controls (0.625% DMSO and 10 μM PZQ in 0.625% DMSO). A Kruskal-Wallis ANOVA followed by Dunn's multiple comparisons test was performed to compare each population mean to DMSO mean. ∗ and ∗∗ represent *p* < 0.0332 and *p* < 0.0021, respectively.

Further investigation of these 9 compounds (compared to compound 1) aimed to quantify their effect on the adult stage of the parasite (Fig. 3). Each compound was initially tested at 12.5 μM, which we identified as a sublethal concentration for compound 1 (Fig. 1C) and then at 6.25 and 3.15 μM. While all compounds demonstrated a dose-dependent effect on adult worm motility, six (11, 16, 19, 20, 22 and 23) were particularly active at 12.5 and 6.25 μM. Compounds 11, 19 and 22 caused complete cessation of worm mobility even at the lowest concentration tested (3.15 μM), resulting in the most active compounds within the series.

The biological evaluation of these 24 compounds highlights three main series based on their effects on phenotype and motility of *S. mansoni* schistosomula (where the most complete dataset has been collected, Table 1). Firstly, six chemicals (2, 3, 4, 8, 14 and 21) did not have any effect on the parasite (even at the higher concentration tested). A weak effect on both metrics was recorded for the second group of compounds (including 5, 6, 7, 12, 13, 15, 18 and 24). Based on the calculated EC_50_ values, three quinoxaline derivatives (9, 10 and 16) showed a comparable anti-schistosomal activity to the hit compound (1, EC_50_ = 4.72 and 4.47 μM on phenotype and motility, respectively). The remaining derivatives (11, 17, 19 and 20) had an EC_50_ value between 1 and 2 μM (same range as praziquantel, PZQ). Above all, compound 22 was the most potent compound with an EC_50_ in the high nanomolar range (EC_50_ = 0.39 μM), comparable to the gold-containing anti-schistosomal compound, Auranofin (AUR) [26,27]. These anti-schistosomula results broadly match the same activity trends observed for adult worms (e.g. Fig. 3).

### 2.3 Preliminary structure-activity relationship (SAR) observations

Comparing the anti-schistosomula activities of these compounds, some trends in structure-activity relationship (SAR) were found to be associated with four distinctive structural features: (1) the central scaffold (organosulfur compounds containing one (as with compounds 2, 3 and 4) or two sulphur atoms (as with compound 1)- or a quinoxaline core - remaining compounds); (2) different substitutions at the C6 position of the central scaffold; (3) the linker between the central scaffold and the aromatic rings; (4) the different substitutions of the aromatic rings (Fig. S2). Interestingly, all compounds that contained both nitrogen and sulphur heteroatoms on the central scaffold were inactive on the parasite (2, 3 and 4; Table 1). The introduction of the quinoxaline scaffold induced a consistent increase in activity (exemplified with comparison between compounds 11 vs 4, 9 vs 3 and 15 vs 2; Fig. S3A). We also identified another general trend in activity decreasing when the nitro group in C6 position was removed (23 vs 10, assuming the methyl/ethyl ester did not significantly affect the *in vitro* activity), or when replaced by other groups such as a methoxy group (19 vs 6; Fig. S3B). Despite the limited number of compounds available, preliminary data suggested a positive contribution of the *N*-linker between the quinoxaline core and the aromatic rings on the anti-schistosomal activity (11 vs 12 and 15 vs 14; Fig. S3C).

The effect of different substituents and different patterns of substitution on the two aromatic rings linked to the central scaffold was investigated next (Fig. S3D). This analysis showed that functionalisation in the *meta* position (position 3 of the aromatic ring) alone (19 vs 20; Fig. S3D) or together with a second group in the *para* position (position 4 of the aromatic ring, such as with compounds 15 vs 16; Fig. S3D) resulted in moderate to good anti-schistosomula activity, particularly when a halogen substituent was introduced (16 vs 17). However, when a group was introduced in the *ortho* position (8 vs 9; Fig. S3D, assuming equal contribution of methoxy- or ethoxy-group on the biological activity), a decrease in anthelmintic activity was observed. Overall, these initial investigations provided insights into the SAR profile of this compound class (summarised in Fig. S3E) and identified the 2,3-bis(phenylamino)quinoxalines as promising candidates for further anti-schistosomal development.

To our knowledge, the anti-schistosomal activity of the quinoxaline core was first demonstrated during the evaluation of the Medicines for Malaria Venture (MMV) malaria box on *S. mansoni* [28]. As part of a ‘piggybacking’ approach, a N_2_,N_3_-bis(4-bromophenyl)quinoxaline-2,3-diamine derivative (MMV007224) had *in vitro* activity in the sub micromolar range and moderate *in vivo* activity. While a different approach was applied herein, our results expand upon these initial observations and further confirm the utility of the quinoxaline core (containing diverse functional groups) as a potential component of next generation anti-schistosomal compounds.

### 2.4 Further anti-schistosomal investigations of the prioritised lead compound (22)

**Fig. 4:**
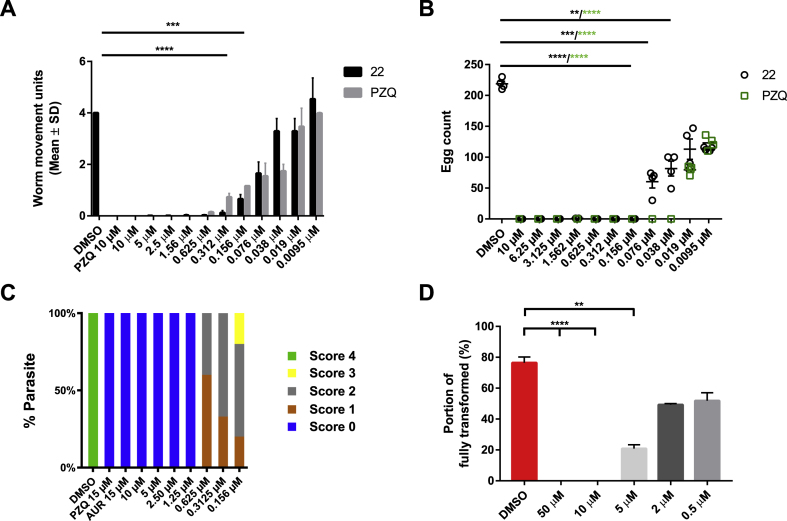
Compound 22 is active on both intra-mammalian and intra-molluscan *S. mansoni* life cycle stages. (A) - A dose response titration (0.0095–10 μM) of compound 22 and PZQ was performed to assess their comparable potencies on *S. mansoni* adult worms. The bar chart shows the average worm movements recorded by WomassayGP2 in two independent screens (each screen contained two technical replicates). The average of each compound was shown in comparison to the standard controls (0.625% DMSO and PZQ 10 μM). (B) - After 72 h, eggs were enumerated in both compound 22 and PZQ treatment groups and reported in the scatter chart. For each concentration tested, the mean of the egg count and the standard error across the two biological and technical replicates were represented on the graph. (C) - Juvenile *S. mansoni* worms (3 weeks post infection; n = 10–25) were cultured in different concentrations of compound 22 (0.156, 0.312, 0.625, 1.25, 2.50, 5 and 10 μM in 1.25% DMSO) and motility scored (0 = dead, 4 = normal movement) at 72 h. Control parasites (n = 10–25) include those co-cultivated in the presence of 1.25% DMSO and PZQ and AUR (both 15 μM in 1.25% DMSO). (D) The effect of compound 22 (0.5, 2, 5, 10 and 50 μM) on miracidia transformation was registered in terms of % fully transformed sporocysts enumerated after 48 h incubation. A Kruskal-Wallis ANOVA followed by Dunn's multiple comparisons test was performed to compare each population mean to DMSO mean. ∗∗, ∗∗∗, ∗∗∗∗ represent *p* < 0.0021, *p* < 0.0002, *p* < 0.0001, respectively.

Due to its strong anti-schistosomula (EC_50_ = 0.39 μM, Table 1) and adult worm (motility was completely inhibited at 3.15 μM, Fig. 3) activities, compound 22 was further titrated against adult worms and assessed for juvenile worm and miracidia-sporocyst transformation inhibition actions (Fig. 4).

Initially, the potency of compound 22 on adult worms was directly compared to PZQ. Here, a dose-response titration of both PZQ and compound 22 (from 0.0095 μM to 10 μM) demonstrated that both compounds induced a comparable effect on worm motility (Fig. 4A). Oviposition was similarly inhibited by both treatments with IVLEs being produced only in cultures containing nanomolar concentrations of either compound (PZQ performed slightly better than compound 22; Fig. 4B). When next titrated on juvenile worms (Fig. 4C), compound 22 induced parasite death down to 1.25 μM (supported by propidium iodide, PI, staining, Figs. S4A and B). At lower concentrations (down to 0.156 μM), compound 22 led to increased granularity (Fig. S4C) and reduced movement (V1 in Supplementary Data), but not lethality (supported by a reduced PI uptake; Fig. S4). This compound showed an EC_50_ of 0.201 μM (0.118–0.271 95% CI). Finally, compound 22 also demonstrated a concentration-dependent inhibition to miracidial transformation (Fig. 4D). This compound was lethal to this parasite stage at the higher concentrations tested (10 and 50 μM); at 5 μM, miracidia to sporocyst transformation was significantly reduced by 50%, compared to DMSO. Miracidial transformation inhibition declined to about 20% when the compound was tested at 2 and 0.5 μM. Collectively these data demonstrate the broad-spectrum activities (e.g. decreases in motility, oviposition and viability) of compound 22 on diverse (intra-mammalian and intra-molluscan) *S. mansoni* lifecycle stages.

### 2.5 Evaluation of compound 22 induced genotoxicity and cytotoxicity

**Fig. 5:**
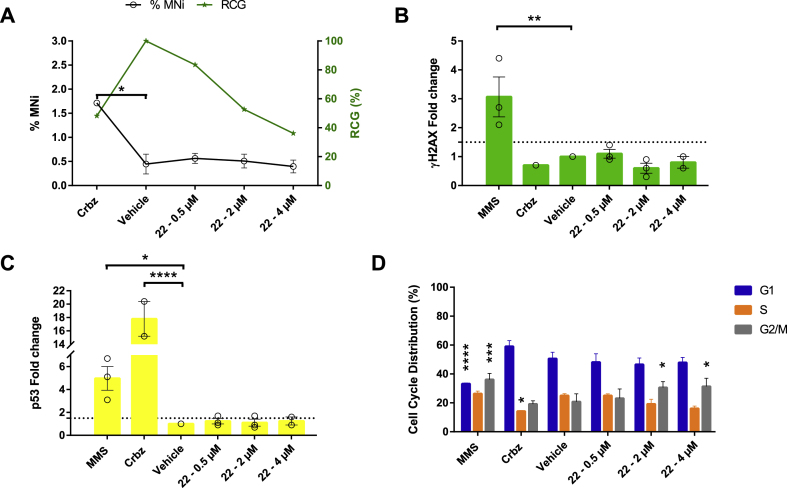
Compound 22 exhibits some cytotoxic, but no genotoxic activities in TK6 cells. (A) – Quantification of %MNi induction in TK6 cells treated with three doses of compound 22 (0.5, 2 and 4 μM) compared to the negative (1% DMSO; vehicle) and positive (carbendazim; Crbz, 8.4 μM) controls, after 1.5–2 cell-cycles (24 h). MNi were scored from the mononucleated images collected on the ImageStream Mark II using the DeepFlow neural network algorithm as described in Wills et al. [77]. Graph shows the mean value of %MNi ± SD (n = 3, except for Crbz and the highest concentration tested of compound 22; n = 2). Relative Cell Growth (RCG, as %) is also displayed. (B) - ɣH2AX observed in TK6 cells co-cultivated with either 0.5, 2 or 4 μM compound 22 compared to the positive (45.4 μM MMS and 8.4 μM Crbz) and negative (1% DMSO; vehicle) controls. The dotted line represents the fold change cut off value of 1.5. (C) - p53 measured in the same six TK6 cell/compound co-cultures described in (B). The dotted line represents the fold change cut off value (1.5). For (B) and (C), each experimental point is shown (n = 3, except for Crbz and the highest concentration tested of compound 22; n = 2). Error bars represent SD. For (A), (B) and (C) any significant difference compared to the DMSO control was quantified by a one tailed Dunnett's test (∗, ∗∗ and ∗∗∗∗ representing *p* < 0.0332, *p* < 0.0021 and *p* < 0.0001, respectively). (D) Cell-cycle analyses conducted with compound 22. The percentage of TK6 cells in each cell-cycle phase (G1, S and G2/M) is shown for compound 22 treated cells as well as the positive and negative controls (n = 3). For each phase, any significant difference compared to the DMSO control was indicated as ∗, ∗∗∗, ∗∗∗∗ representing *p* < 0.0332, *p* < 0.0002, *p* < 0.0001, respectively (by two tailed Dunnett's test).

Assessment of compound genotoxicity/cytotoxicity is a key stage in drug development and can be performed at early or late stages of the pipeline [[29], [30], [31]]. Whereas compound-associated cytotoxicity can be mitigated by medicinal chemistry and does not necessarily stop progression of a series, compound-associated genotoxicity represents a red flag and could halt progression [29,32,33]. Therefore, *in vitro* assays were conducted to assess whether compound 22 (or its metabolites) had the potential to cause DNA damage in a surrogate human cell line (TK6 cells; Fig. 5). Alteration to the chromosomal segregation machinery (chromosomal loss and chromosomal breaks; aneugenicity and clastogenicity, respectively) was first evaluated via micronuclei (MNi) formation in cells that have undergone mitosis (Fig. 5). Based on preliminary 24 h cytotoxicity data generated for compound 22 (CC_50_ = 9.36 μM; Fig. S5A), three sub-CC_50_ concentrations (0.5, 2 and 4 μM) were assessed on TK6 cells in addition to negative (DMSO, used as solvent for compound preparation) and positive (Carbendazim, Crbz) controls (Fig. 5A). The number of MNi did not change significantly (compared to DMSO treated cells) across the three assayed concentrations of compound 22. However, there was a slight decrease at the higher concentration tested (4 μM). This evidence is in line with the increased mitotic arrest at this higher dose as shown by the Cell Count Relative Cell Growth (RCG) data.

Two additional biomarkers of genotoxicity were also used in this investigation: ɣH2AX (phosphorylated histone variant H2AX at serine 139) to detect DNA double-strand breaks [34,35] and nuclear p53 content to discriminate levels resulting from increased DNA damage compared to levels normally observed in replicating cells [36]. The fold change of both p53 and ɣH2AX biomarkers in TK6 cells when exposed to different concentrations of compound 22 did not exceed the criteria for a positive result (Fig. 5B and C; cut off values of 1.5 for ɣH2AX and p53 [30,[37], [38], [39]]). In contrast, the positive (Methyl Methanesulphonate - MMS - and Carbendazim - Crbz - as representative clastogenic and aneugenic agents, respectively [36,40]) and negative (solvent or vehicle) controls behaved as expected according to Takeiri et al. [40]. From these data, we concluded that compound 22 induced little or no DNA damage and, at these concentrations, does not induce genotoxicity.

Follow-on cell-cycle analysis (Fig. 5D) revealed a G2/M phase arrest induced by compound 22 at 2 and 4 μM (mirroring the RCG results in Fig. 5A). These findings, along with low levels of MNi, ɣH2AX and p53 indicated a cell cycle effect most likely due to compound 22-induced cytotoxicity (e.g. apoptosis or necrosis) at these concentrations. During the genotoxicity studies, we observed that compound 22 displayed autofluorescence properties and was almost exclusively localised to the cytoplasm of TK6 cells (Fig. S5B). These results were confirmed in three additional human cell lines (HeLa, human cervical epithelial; MDA-MB-231, human breast epithelial; HepG2, human liver epithelial) (Fig. S6). Compound 22, thus, accumulates in the cytoplasm and displays some cytotoxic, but no genotoxic, properties; medicinal chemical modifications of this lead was next pursued to obtain more potent and less cytotoxic quinoxaline analogues.

### 2.6 Medicinal chemistry optimisation of compound 22

**Scheme 1:**
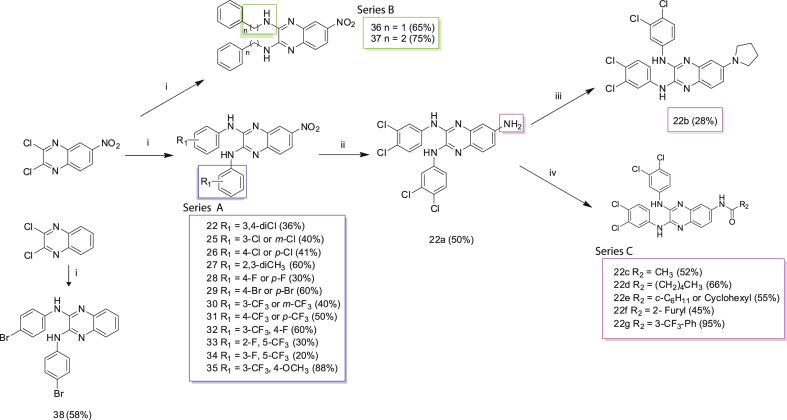
Synthesis of the 2,3-bis(phenylamino) quinoxaline series. The scheme shows the re-synthesis of compound 22 and the synthesis of 20 related derivatives (grouped by structural similarity in Series A, B and C - highlighted with blue, green and magenta boxes, respectively). Compound 38 (same as MMV007224, previously identified during the anti-schistosomal screening of the Medicines for Malaria Venture (MMV) malaria box collection) was synthesised as reference for the compounds synthesised and tested in this study. Reagents and conditions: (i) different substituted anilines (compounds 22–35) or phenyl-alkyl amines (36 and 37), anhydrous DMSO, 130 °C, 30 min; (ii) H_2_, cat. Pd/C, AcOEt, rt, 2h; (iii) Br(CH_2_)_2_Br, K_2_CO_3_, CH_3_CN, MWI (300 W), 150 °C, 15 min; (iv) R_2_COCl, anhydrous Pyr, anhydrous DCM, 0 °C → rt, 1 h. Compound yield was reported in brackets.

Based on the biological data described thus far and the identification of compound 22 as a potent lead, we next explored the impact of chemical structure modifications on anti-schistosomal activity aiming to obtain more potent and less cytotoxic quinoxaline analogues. The lead optimisation approach aimed to first explore the SAR around the aryl moiety, second to investigate the influence of the *N*-linker and third to functionalise the C6 position of the central scaffold. Towards this goal, three compound series (Series A, B and C, clustered based on their chemical similarity) were identified leading to the creation of 21 compounds (re-synthesis of compound 22 and 20 additional analogues, Scheme 1). In brief, 2,3-bis(phenylamino)-quinoxalines were obtained from the reaction of 2,3-dichloro-6-nitroquinoxaline with different substituted anilines (resynthesis of compound 22 and synthesis of compound 23–35, Series A) or phenyl-alkyl amines (compound 36 and 37, Series B) as previously described [41,42]. The yields of the reactions reported in Scheme 1 show that the derivatives were obtained in varying amounts reflecting the different reactivity of the anilines and phenyl-alkyl amines used in the reaction. Furthermore, an analogue of compound 29 (N_2_,N_3_-bis(4-bromophenyl)quinoxaline-2,3-diamine, here labelled as compound 38) was synthesised by a similar synthetic route (starting from 2,3-dichloro-quinoxaline, Scheme 1) as this chemical (MMV007224) was previously identified during anti-schistosomal screening of the Medicines for Malaria Venture (MMV) malaria box collection [28]. Therefore, compound 38 was suitable for comparing the analogues created in this study. In preparation for the synthesis of Series C compounds, the classical catalytic hydrogenation of the 6-nitro-substituted quinoxaline 22 into the amino derivative (compound 22a) was performed [43,44]. A microwave-assisted reaction was subsequently applied to the 6-amino-substituted quinoxaline and dibromo butane in acetonitrile for the synthesis of compound 22b (a Series C compound). The further synthesis of *N*-acyl compound 22 derivatives was carried out as previously described [45]. Functionalisation of the amine group contained within compound 22a was performed with the appropriate acyl derivative and an excess of anhydrous pyridine in Dichloromethane (DCM) generating the derivatives 22c-22g in 45–95% yield (remaining Series C analogues; Scheme 1).

### 2.7 Biological activities of the compound 22 analogues

**Fig. 6:**
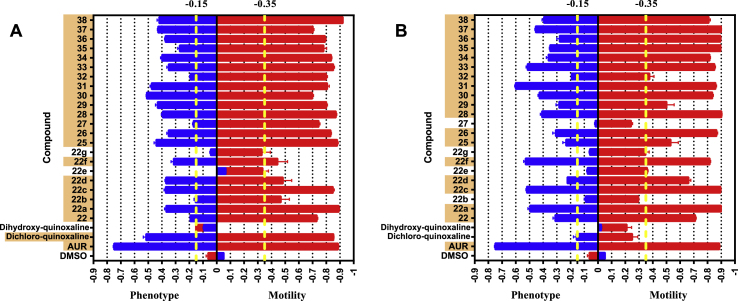
Anti-schistosomal effects of newly synthesised compounds. The effect of each compound at 50 (A) and 10 μM (B) on 72 h cultured schistosomula was assessed in duplicate in three independent screens. Positive (Auranofin, AUR) and negative (DMSO) controls were included in each screen. Here the effect on the phenotype and the motility of the parasite (phenotype – on the left - and motility – on the right - score) for each compound was shown as an average score from the different biological replicates. In total, four screens were performed and the Z′ calculated for both phenotype and motility obtained for each screen was reported in Table S4. The hit boundaries for both motility (- 0.35) and phenotype (- 0.15) are shown in yellow (dotted line). All compounds showing a score lower than both reference values were considered hits (highlighted in orange). Error bars were included to represent the standard error for the average score of the different screens. In both (A) and (B), the entries ‘dichloro-quinoxaline’ and ‘dihydroxy-quinoxaline’ represent 2,3-Dichloro-6-nitroquinoxaline and its hydroxy derivative (6-nitro-2,3-dihydroxyquinoxaline), respectively.

All newly synthesised compounds (22, 22a-g, 25–38 - 22 compounds in total) were initially screened against 72 h-cultured schistosomula at 10 and 50 μM concentrations (Fig. 6). In these screens, the starting synthesis material (2,3-dichloro-6-nitroquinoxaline) and its derivative (6-nitro-2,3-dihydroxy-quinoxaline) were also included to fully explore the SAR around the quinoxaline scaffold. These 24 compounds were screened alongside controls (AUR and DMSO) and 21 of these (87.5% hit rate) demonstrated activity at 50 μM (Fig. 6A). At the tested concentration (50 μM), all compounds affected both phenotype and motility of the schistosomula except for compound 22b; this compound affected schistosomula motility, but not phenotype. Motility scores for three compounds (22a, 25 and 38) were comparable to the positive controls (AUR treated schistosomula), although phenotype scores were not as severe. At the lower concentration (10 μM), only 18 (75% hit rate) of these compounds remained active (Fig. 6B).

To further assess the potency of the 18 hits found to be active at 10 μM, a dose-response titration (0.313–10 μM) was conducted on schistosomula (Table 2 and Table S4). This extensive investigation identified three main bioactive groups: a smaller group (including 22a, 22c, 36, 38) with an EC_50_ > 2 μM; a second group having improved activity (with 1 μM < EC_50_ < 2 μM) and a final set of 8 chemicals with a potency in the high nanomolar range (EC_50_ < 1 μM). Collectively, the *in vitro* screens highlighted three compounds (30, 31, 33) with highly improved anti-schistosomula activities when compared to lead compound 22. For comparison, the positive controls AUR and PZQ demonstrated EC_50_ values against schistosomula of 0.41 and 1.01 μM, respectively (in agreement with previous studies [28,[46], [47], [48]]).

**Table 2:** Anti-schistosomal and HepG2 cytotoxic activities of the newly synthesised quinoxaline derivatives.

	Activity on schistosomula (EC_50_, μM - 72 h)			Activity on adult worms (EC_50_, μM - 72 h)	HepG2 cells (CC_50_, μM)		Selectivity Index (SI)	
Compound	P	M	Average	Motility	24 h	72 h	Somula	Adult worms
2,3-dichloro-6-nitroquinoxaline	>10	>10	ND	ND	27.51	ND	ND	ND
6-nitro-2,3-dihydroxy-quinoxaline	>50	>50	ND	ND	136.40	ND	ND	ND
22	0.47	0.42	0.44	84.7 × 10^−3^	7.40	0.26	0.59	3.11
22a	3.51	3.25	3.38	ND	25.11	ND	ND	ND
22b	>10	>10	ND	ND	ND	ND	ND	ND
22c	3.04	2.79	2.91	703.5 × 10^−3^	8.60	4.59	1.57	6.52
22d	1.02	1.80	1.41	318.3 × 10^−3^	64.75	2.43	1.72	7.65
22e	>50	>50	ND	ND	ND	ND	ND	ND
22f	1.91	2.00	1.95	ND	14.84	5.69	2.91	ND
22g	>50	>50	ND	ND	ND	ND	ND	ND
25	1.22	1.30	1.26	11.99 × 10^−3^	6.76	0.58	0.46	47.99
26	0.42	0.39	0.41	20.31 × 10^−3^	13.05	0.13	0.32	6.33
27	>10	>10	ND	ND	ND	ND	ND	ND
28	2.14	1.40	1.77	381.6 × 10^−3^	10.16	ND	ND	ND
29	0.78	0.65	0.71	122 × 10^−3^	8.72	3.97	5.55	32.52
30	0.34	0.43	0.38	2.59 × 10^−3^	5.85	0.90	2.36	347.59
31	0.34	0.28	0.31	7.7 × 10^−3^	8.80	0.08	0.26	10.21
32	0.89	0.54	0.72	11.01 × 10^−3^	7.75	0.67	0.94	60.99
33	0.15	0.15	0.15	5.60 × 10^−3^	27.52	0.10	0.66	17.87
34	0.37	0.43	0.40	6.47 × 10^−3^	10.07	ND	ND	ND
35	0.63	0.69	0.66	44 × 10^−3^	5.64	0.43	0.65	9.73
36	3.19	2.64	2.91	ND	11.67	5.08	1.74	ND
37	2.20	1.47	1.84	195 × 10^−3^	4.40	4.52	2.47	23.20
38	3.67	1.21	2.44	162 × 10^−3^	7.12	4.64	1.90	28.65
AUR	0.37	0.45	0.41	ND	5.07	2.94	7.17	ND
PZQ	1.14	0.88	1.01	106.90 × 10^−3^	142.8	101.05	100.05	945.31

Schistosomula EC_50_ (Phenotype - P - and Motility - M) values of the new compounds were calculated based on three replicate titrations (0.313–10 μM). Similarly, EC_50_ values were calculated for Auranofin (AUR) and praziquantel (PZQ), included here for comparison. Where no effect was seen on schistosomula at the tested concentrations (10 and 50 μM), the EC_50_ was said to be higher than 50 μM. The EC_50_ was said to be higher than 10 μM if the compound was a defined hit at 50 μM, but not at 10 μM. An average schistosomula EC_50_ (Phenotype and Motility) values is also provided. Adult worm EC_50_ values of the new compounds were calculated based on three replicate titrations (0.0095–5 μM). Each titration was performed in duplicate and repeated. Worm movement was recorded with WormassayGP2. Praziquantel and auranofin (10 μM in 0.625% DMSO; positive controls) as well as DSMO (0.625%, negative control) were also included in the schistosomula and adult worm screens. Each compound was tested against the human HepG2 cell line at the concentration range spanning 200 μM–0.01 μM (three technical replicates each). The average CC_50_ values of each compound were calculated from three or two replicates after 24 or 72 h co-incubation, respectively. All data were analysed using GraphPad Prism. The Selectivity Index (SI) of the compounds on schistosomula and adult worms was calculated based on 72 h CC_50_ values and schistosomula and adult worms EC_50_ values, respectively. ND: not determined (where titration was not performed so EC_50_ value was not available and arithmetic average/selectivity index was not calculated). Refer to Table S7 for the 95% CI (Confidence Interval) values.

To determine if these analogues also displayed activity against adults, a dose response titration on adult worm pairs was performed with 15 of the most potent anti-schistosomula compounds (Table 2). Here, all the tested compounds displayed anthelmintic activity with 6 compounds (22c, 22d, 28, 29, 37 and 38) displaying EC_50_ values in the high to mid-nanomolar range (less potent than compound 22). The other compounds (25, 26, 30, 31, 32, 33, 34 and 35) showed a marked increase in activity when compared to the lead compound 22, with compounds 30, 31, 32, 33 and 34 representing the most potent. Notably, while not the most potent compound in the series, the adult worm activity registered for compound 38 was comparable to previous reports [28] and similar to praziquantel itself [28,46]. Reassuringly, the re-synthesised compound 22 showed identical anti-schistosomal activities as the original commercially sourced compound on both schistosomula (0.51 μM - 95% CI 0.43 to 0.59 - and 0.61 μM - 95% CI 0.53 to 0.72, respectively) and adult worms (73.14 nM - 95% CI 66.74–79.54- and 68.50 nM - 95% CI 63.50–73.50, respectively; Fig. S7).

**Fig. 7:**
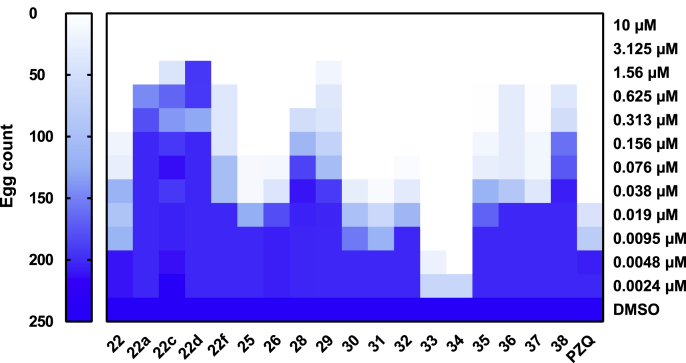
Comparative anti-fecundity activity of compound 22 and its structural analogues. Each compound was screened on *S. mansoni* adult worms and the effect of each compound on egg production (after 72 h) was recorded at each concentration tested (0.0024–10 μM in 0.625% DMSO v/v). The egg count derived from adult worms co-cultured in DMSO (0.625% v/v) was included as a negative control. Data were shown as mean average across the replicates and presented as a heat map (white to blue gradient scale; white – lowest egg count, dark blue – highest egg count).

The anthelmintic activity of these compounds was also assessed in terms of IVLE production (Fig. 7). While most compounds inhibited oviposition at low concentrations (low nanomolar range), some were not as effective (e.g. 22a, 22c, 22d, 28, 29 and 38) as compound 22. Interestingly, compounds 33 and 34 were even more potent than PZQ (and compound 22) in affecting this important aspect of schistosome lifecycle maintenance and pathology development (Fig. 7).

**Fig. 8:**
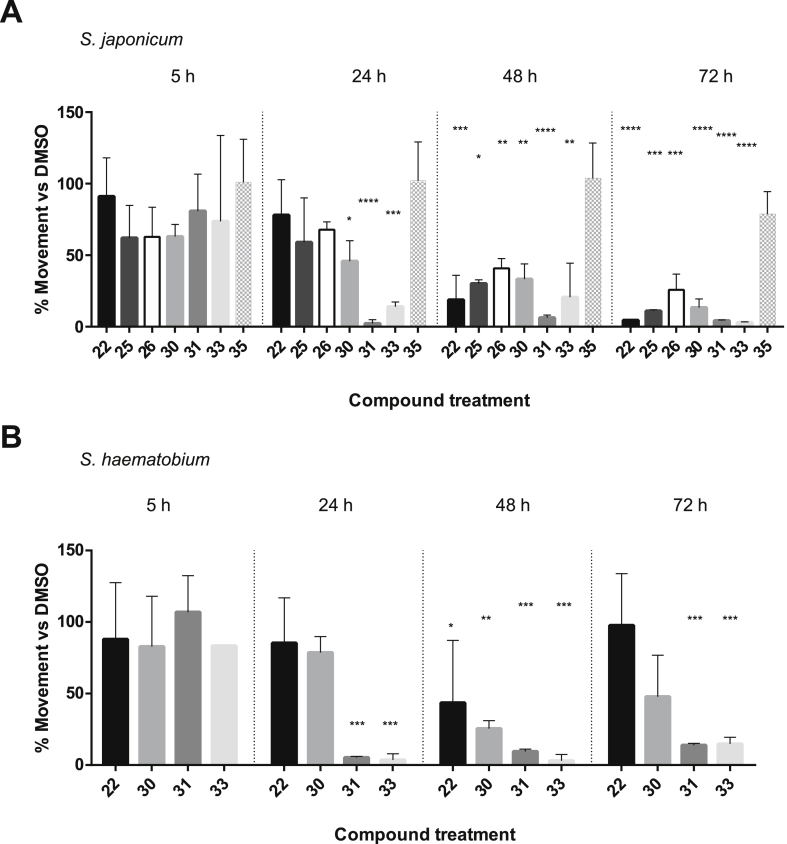
Compound 22 analogues are active against *S. japonicum* and *S. haematobium* adults. (A) - Seven compounds (22, 25, 26, 30, 31, 33 and 35) were screened against *S. japonicum*. Each compound was tested at 100 nM in triplicate (except for compounds 25 and 33, tested in duplicate). (B) - Four compounds (22, 30, 31 and 33) were screened against *S. haematobium*. Each compound was tested at 100 nM in triplicate. The negative control (DMSO) was included in each experimental screen. Parasite worm movement was recorded at 5, 24, 48 and 72 h by WormAssay. The biological results were represented as the percent reduction in compound mediated motility compared to DMSO controls and displayed as a bar chart (average motility across the replicates + SD). A one-way ANOVA followed by Dunnett's multiple comparisons test was performed to compare each treatment to DMSO. ∗, ∗∗, ∗∗∗, ∗∗∗∗ represent *p* < 0.0332, *p* < 0.0021, *p* < 0.0002, *p* < 0.0001, respectively.

Based on the *S. mansoni* adult worm activities (Table 2), the seven most active compounds (22, 25, 26, 30, 31, 33 and 35) were then screened against adult *S. japonicum* male worms (Fig. 8). Here, a single-point concentration (100 nM, based on EC_50_ values calculated on *S. mansoni* adult worms) screen was performed to assess the effect of these compounds on worm motility at four different time-points (5, 24, 48 and 72 h). On *S. japonicum*, compounds 31 and 33 decreased parasite motility as early as 24 h post treatment and this remained the case for the entire 72 h of the assay (Fig. 8A, V2). Based on phenotypic descriptors, these compounds were essentially lethal after 72 h (Table S5). Among the remaining compounds, the quinoxaline derivatives 22, 25, 26 and 30 were not lethal over the 3-day incubation, but they all decreased motility starting at 48 h. Conversely, compound 35 showed no appreciable activity against *S. japonicum* at the selected concentration. A subset of these compounds (22, 30, 31 and 33) was subsequently screened (at 100 nM) against the urinary blood fluke *S. haematobium*. Similar to their effects on *S. japonicum*, compounds 31 and 33 demonstrated the best activity on *S. haematobium* motility (Fig. 8B, V3, Table S6). Of the two remaining compounds, 22 was mainly inactive and 30 had relatively mild effects on worm movement and plate attachment. (Fig. 8B, Table S6). For both *S. japonicum* and *S. haematobium,* compounds 31 and 33 were the most active in terms of severity of phenotypic response and decrease in worm motility as well as being fast-acting compounds (onset time within 24 h). In addition, *S. japonicum* appeared to be more susceptible to these two compounds than *S. haematobium*. When we cross-compared the activity of these compounds on *S. japonicum* and *S. haematobium* species, the trend of activity was broadly consistent with the biological data collected on *S. mansoni* (Table 2). Thus, compounds 31 and 33 were the most active against all three species, whereas compound 35 was the least active. Interestingly, the lead compound 22 was not as potent (at 100 nM) on *S. haematobium* when compared to either *S. japonicum* or *S. mansoni*. While we have yet to explain this discrepancy, differences in activities between the schistosome species might be influenced by factors such as membrane composition, intra-worm compound stability/compound metabolism or sequence variations within the active site of the putative (yet to be identified) target.

**Fig. 9:**
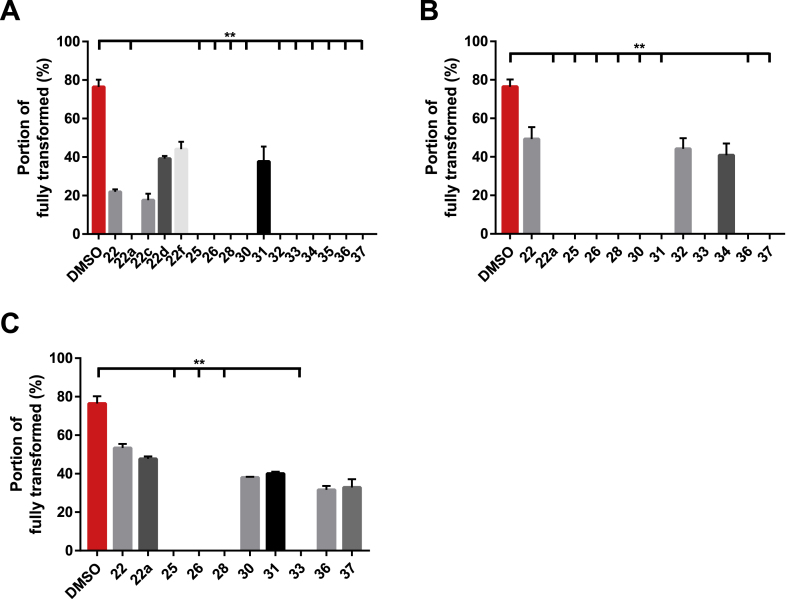
Structural analogues of compound 22 block miracidia to primary sporocyst transformation. (A) - All 16 compounds (at 5 μM in 1% DMSO) were assessed for their ability to block miracidial to sporocyst transformation and compared to compound 22 (at 5 μM in 1% DMSO) and DMSO (negative control, 1% DMSO). (B) - All compounds lethal at 5 μM were then screened at 2 μM. (C) - Another screen at 0.5 μM was performed to assess the activity of the most potent compounds derived from the 2 μM screen. Each treatment was set up in triplicate and parasites were cultured in CBSS at a controlled temperature of 26 °C (in the dark). A Kruskal-Wallis ANOVA followed by Dunn's multiple comparisons test was performed to compare each population mean to DMSO mean. ∗, ∗∗, ∗∗∗, ∗∗∗∗ represent *p* < 0.0332, *p* < 0.0021, *p* < 0.0002, *p* < 0.0001, respectively.

After examining the newly synthesised compounds’ activities against two lifecycle stages of *S. mansoni* found in the mammalian host (schistosomula and adults) and worms of the two other medically important species *S. haematobium* and *S. japonicum*, we next investigated their potential in blocking miracidia to sporocyst transition in *S. mansoni* (Fig. 9). Based on the dose-dependent effect on miracidial transformation observed with compound 22 (Fig. 4), 16 compounds (the lead compound 22 and 15 analogues) were subsequently screened to assess their activity on miracidial transformation (Fig. 9). The first screen was performed at 5 μM as this concentration was not lethal to miracidia cultured in the presence of the lead compound 22 (Fig. 9A). At this concentration, 11 compounds had a lethal effect on the parasite and, therefore, were more active than the lead compound. A second screen at 2 μM was then performed with these 11 compounds in comparison to the same concentration of compound 22 (Fig. 9B). Here, 9 compounds killed the parasite and completely inhibited transformation to sporocyst. However, only 4 compounds were lethal for miracidia at the lowest concentration tested (0.5 μM, Fig. 9C). In conclusion, these three screens led to the identification of compounds with an increased potency on miracidia transformation when compared to the lead compound. Four compounds (including compound 33) also caused parasite death at the lowest concentration tested.

As compound 22 demonstrated some cytotoxicity (Fig. 5 and Fig. S5), the cytotoxic activity of these analogues was next explored on human HepG2 cells by prioritising those which showed the most potent anti-schistosomal activity (EC_50_ on schistosomula below 10 μM). Each compound was initially tested for 24 h in a dose response titration (0.01–200 μM) with the average CC_50_ of each compound reported in Table 2. As shown, most compounds induced some degree of cytotoxicity against the HepG2 cell line (including compound 22, which confirms results presented in Fig. 5 and Fig. S5A). Compounds 30, 35 and 37 were found to be the most toxic among this family of compounds having the lowest CC_50_ values (5.85, 5.64 and 4.40 μM respectively). Nine compounds (22, 22c, 22f, 21, 28, 31, 32, 34 and 36) showed an intermediate CC_50_ between 6 and 20 μM. In contrast, compounds 22a, 22d, 28 and 33 showed low toxicity. The cytotoxicity was also evaluated after 72 h incubation and the results broadly recapitulated the trend seen during the 24 h screens (Table 2). When compared to EC_50_ values derived from schistosomula and adult schistosome screens at 72 h, a selectivity index (SI) for some of the newly synthesised compounds was subsequently calculated (Table 2). The analysis of these values highlighted that the lead optimisation of compound 22 led to the identification of compounds with a much-improved anti-schistosomal activity/(surrogate) host safety ratio (compounds 22c, 22d, 25, 26, 29, 30, 31, 32, 33, 35, 37 and 38).

During the preparation of this manuscript, an anti-schistosomal evaluation on a small subset of compounds structurally related to the quinoxaline derivatives presented in this study (particularly 22, 22c, 25, 26, 29 and 30) was published [49]. Starting from a previously described dianilinoquinoxaline (MMV007224) [28], this recent study focused on the investigation of other structurally-related analogues. While some biological discrepancies were observed between our study and that conducted by Debbert *et al.* [49], likely due to differentially-employed experimental processes [50], broadly overlapping anti - *S. mansoni* activities were found with these six compounds. However, we herein have added a substantial body of additional work that more broadly explored the anti-schistosomal activities of both dianilinoquinoxalines and 6-nitroquinoxalines (or 6-acyl derivatives); these supporting data include activities against *S. mansoni* juveniles (compound 22 only) and sporocysts (compounds 22, 22a, 22c, 22d, 22f, 25, 26, 28, 30, 31, 32, 33, 34, 35, 36 and 37) as well as adult *S. japonicum* (compounds 22, 25, 26, 30, 31, 33 and 35) and *S. haematobium* worms (compounds 22, 30, 31 and 33). Using our collective anthelmintic and cytotoxic information gathered in this study, and informed by others [49], an expansion of the preliminary SAR (introduced in Fig. S3) around the quinoxaline analogues is now possible.

## 3 Structure-activity relationship (SAR) studies on the anti-schistosomal quinoxaline derivatives

Our first objective assessed whether the quinoxaline scaffold contributed to the *in vitro* anti-schistosomal activity and then subsequently explored the effects of the linker between the central quinoxaline core and the aromatic rings. As shown in Table 3, the quinoxaline moiety was not solely responsible for the anti-parasitic activity since 2,3-dichloro-6-nitroquinoxaline or its dihydroxy derivative (6-nitro-2,3-dihydroxy-quinoxaline) had poor to moderate activity against schistosomula.

**Table 3:** SAR study around the central quinoxaline scaffold.

				Anti-schistosomal activity EC_50_^a^ (μM)				
				*Schistosomula*		*Adult*	Cytotoxic activity CC_50_^c^ (μM)	
	Compound	R_1_	R_2_	Phenotype	Motility	Motility	24 h	72 h
	2,3-dichloro-6-nitroquinoxaline	Cl	Cl	>10	>10	N.D.^b^	27.51	N.D.^b^
	6-nitro-2,3-dihydroxy-quinoxaline	OH	OH	>50	>50	N.D.^b^	136.4	N.D.^b^
	22			0.47	0.42	0.085	7.4	0.26
	19			1.89	1.35	N.D.^b^	12.59	N.D.^b^
Series B	36			3.19	2.64	N.D.^b^	11.67	5.08
	37			2.20	1.47	0.195	4.40	4.52

a 50% Effective concentration, or compound concentration required to inhibit parasite movement by 50%;

b N.D.: not determined.

c 50% Inhibitory concentration, or compound concentration required to inhibit cell proliferation by 50%.

With regards to the linker between the quinoxaline central core and the aromatic ring, we previously observed that the dianilinoquinoxalines were more active than the 2,3-bis(arylthio)- or diphenoxyquinoxalines (Fig. S3C). However, this feature had not been identified previously [49] and this observation, derived from a limited number of available compounds, will likely require further investigation. Among the dianilinoquinoxalines, we also gathered some preliminary observations around the length of *N*-linker. This linker seemed to be quite a flexible feature since the *N*-methylene derivative (36) and *N*-ethyl derivative (37) showed similar activity to the parental compound 19 (Table 3). The analysis of HepG2 cytotoxicity added some additional information regarding the *N*-linker (Series B, Table 3). In contrast to the anti-schistosomal activities, the *N*-linker length seemed to affect the cytotoxicity of the compounds. In fact, compound 19 displayed moderate toxicity (CC_50_ = 12.59 μM). The introduction of the *N*-methylene linker (compound 36, CC_50_ = 11.67 μM) did not significantly affect the toxicity which increased instead with the introduction of the *N*-ethyl linker (compound 37, CC_50_ = 4.40 μM). To conclude, this region of the molecules could be more critical for host toxicity than for anthelmintic activities.

We next expanded our investigation to include a different set of R-groups on the aromatic rings and to assess the effect of these groups on both parasite and HepG2 cells (Series A, Table 4). Firstly, we deduced that the di-chloro substitution was not essential for the activity as one of the two synthesised mono-chloro-substituted compounds (26) showed similar anti-schistosomal potency as the lead compound (22). Moreover, there is a consistent difference in activity between the two structural isomers (25 and 26) with a preference for the *para* substitution (26) in schistosomula; interestingly, an opposite trend was observed with adult worms (preferred *meta* substitution). As described in the preliminary SAR investigations (Fig. S3), the *meta* and *para* substitution was preferred to the *ortho* position. The anti-schistosomal results obtained with the compound 22 analogues further supported this statement. For example, compound 20 (with only one methyl group in the *meta* position, Fig. S3) displayed good anti-schistosomula activity (EC_50_ = 1.42 μM) whereas the introduction of another methyl group in the *ortho* position of the aromatic ring (27) decreased anti-schistosomula activity (EC_50_ > 10 μM, Table 4). The substitution of the aromatic ring with a trifluoromethyl group led to compounds 30 and 31 showing increased anti-schistosomal activity (especially against the adult stage). The positive contribution of this trifluoromethyl substituent was verified by the increased anthelmintic potency found for compounds 32, 33 and 34 compared to compound 28 (*p*-fluoro derivative) on both schistosomula and adult stages of the parasite; similar conclusions can be drawn for compound 35 where the trifluoromethyl group was combined with a methoxy group. Cytotoxic analysis of the *N*-aromatic derivatives was instead more complicated; no consistent trend was observed except for noticing an increased toxicity associated with improvement in anti-schistosomal activity (the only exception being compound 33). Moreover, the differential effect of *meta*/*para* substitution at the two cytotoxicity endpoints was generally observed (higher toxicity of *meta* and *para* substitution at 24 and 72 h, respectively). Obviously, a longer co-incubation of the compounds resulted in an increased compound-induced toxicity on HepG2 cells (CC_50_ 24 h vs 72 h).

**Table 4:** SAR study of Series A derivatives.

			Anti-schistosomal activity EC_50_^a^			Cytotoxic activity CC_50_^c^ (μM)	
			*Schistosomula* (μM)		*Adult* (nM)		
	Compound	R_1_	Phenotype	Motility	Motility	24 h	72 h
Series A	22	3,4-diCl	0.47	0.42	84.7	7.40	0.26
	25	*m*-Cl	1.22	1.30	11.99	6.76	0.58
	26	*p*-Cl	0.42	0.39	20.31	13.05	0.13
	27	2,3-diCH_3_	>10	>10	N.D.^b^	N.D.^b^	N.D.^b^
	28	*p*-F	2.14	1.40	381.60	10.16	N.D.^b^
	29	*p*-Br	1.06	0.83	122	8.72	3.97
	30	*m*-CF_3_	0.34	0.43	2.59	5.85	0.90
	31	*p*-CF_3_	0.34	0.28	7.70	8.80	0.08
	32	3-CF_3_, 4-F	0.89	0.54	11.01	7.75	0.67
	33	2-F, 5-CF_3_	0.15	0.15	5.60	27.52	0.10
	34	3-F, 5-CF_3_	0.37	0.43	6.47	10.07	N.D.^b^
	35	3-CF_3_, 4-OCH_3_	0.63	0.69	44	5.64	0.43

a 50% Effective concentration, or compound concentration required to inhibit parasite movement by 50%;

b N.D.: not determined;

c 50% Inhibitory concentration, or compound concentration required to inhibit cell proliferation by 50%.

Subsequently, the effect of a small number of C6 position functionalisation on the central scaffold was analysed (Table 5). In fact, the preliminary SAR studies (Fig. S3B) identified another general trend with *in vitro* activity decreasing when the nitro group in the C6 position was removed. Similar observations were reported in Debbert *et al*. [49], despite this effect not being consistent across all sets of analogues presented in their study. Firstly, the positive contribution of the nitro group is evident when comparing the two bromo-derivatives (compounds 29 and 38, EC_50_ = 0.71 and 2.44 μM, respectively - Table 2) with a 4-fold decrease of anti-schistosomal activity when the nitro-group was removed (compound 38).

**Table 5:** SAR study of Series C derivatives.

			Anti-schistosomal activity EC_50_^a^			Cytotoxic activity CC_50_^c^ (μM)	
			*Schistosomula* (μM)		*Adult* (nM)		
	Compound	R_1_	Phenotype	Motility	Motility	24 h	72 h
Series C	22	NO_2_	0.47	0.42	84.7	7.40	0.26
	22c	NH_2_	3.51	3.25	N.D.^b^	25.11	N.D.^b^
	22b		>10	>10	N.D.^b^	N.D.^b^	N.D.^b^
	22c		3.04	2.79	703.50	8.60	4.59
	22d		1.02	1.80	318.30	64.75	2.43
	22e		>50	>50	N.D.^b^	N.D.^b^	N.D.^b^
	22f		1.91	2.00	N.D.^b^	14.84	5.69
	22g		>50	>50	N.D.^b^	N.D.^b^	N.D.^b^

a 50% Effective concentration, or compound concentration required to inhibit parasite movement by 50%;

b N.D.: not determined;

c 50% Inhibitory concentration, or compound concentration required to inhibit cell proliferation by 50%.

As presented here, the reduction of the nitro group (in lead compound 22) to an amino derivative (22a) led to a decrease in anti-schistosomal activity of approximately 10-fold. Low anti-schistosomal activity was also identified for compound 22b where the amino group was incorporated into a cyclic ring. Among the acyl derivatives, the introduction of a long alkyl chain (22d) did not substantially decrease parasite activity (against both schistosomula and adult worms). However, a short alkyl chain did decrease activity (compound 22c). Compound 22e and 22g demonstrated the greatest loss in potency and this was associated with its cycloalkane ring. The incorporation of additional aromatic rings in the alkyl chain (22f and 22g) influenced the anti-schistosomal activity with only compound 22f (containing a heteroaromatic ring) maintaining moderate potency. As far as the cytotoxicity profile, improvements in cytotoxicity with the nitro-amine conversion mirrored other data on nitro-containing compounds [51]. In fact, an improved safety profile was registered for all the derivatives of Series C when compared to the lead compound 22. All these data should improve the SAR around the quinoxaline scaffold (Fig. S8), which would direct further chemical optimisation of this series in the future.

## 4 Conclusions

This study presents an extensive investigation around the anti-schistosomal activity of the quinoxaline scaffold. Firstly, phenotypic screening of commercially available small molecules led to the identification of the hit compound 1 (EC_50_ = 4.59 μM on schistosomula). Further exploration around the chemical space of this compound led to the identification of the lead compound 22 with a more promising anti-schistosomal profile (EC_50_ = 0.44 μM and EC_50_ = 84.7 nM for schistosomula and adult stages, respectively) and calculated selectivity indices (SI) of 0.59 and 3.11 for schistosomula and adult stages, respectively. This lead compound underwent extensive medicinal chemistry optimisation, which resulted in the creation of 21 analogues. The *in vitro* profiling of these compounds highlighted a substantial improvement in anti-schistosomal potency, particularly on the adult stage (with EC_50_ in the low nanomolar range). Despite the observed cytotoxicity on human cell lines, some compounds reported in this study had an improved safety profile compared to the initial lead compound (particularly compound 25, 30 and 32 with selectivity indices (SI) on adult worms of 47.99, 347.59 and 60.99, respectively). More interestingly and without literature precedent, we additionally reported activity against *S. mansoni* juveniles (compound 22) and miracidia/sporocyst lifecycle stages as well as *S. japonicum* and *S. haematobium* adults for a subset of the quinoxaline derivatives presented in this study. Based on these collective data, compounds 31 and 33 are extremely promising due to the demonstrated broad-species activity against the three medically important schistosome species.

Overall, these findings reveal the great potential of quinoxaline-containing compounds as drug candidates for schistosomiasis with the identification of more potent/selective compounds in this study representing valid pre-clinical candidates. Towards this end, the 2,3-dianilinoquinoxaline derivative (MMV007224) had significant *in vivo* efficacy in a murine model of schistosomiasis with worm burden reductions between 53.4% (four per oral doses of 100 mg/kg, delivered every 4 h) and 40.8% (single per oral dose of 400 mg/kg) [28]. While the *in vivo* efficacy of MMV007224 was not as high as expected based on *in vitro* potencies, this discrepancy could be explained by protein binding effects. Poor pharmacokinetic properties and *in vivo* efficacies of related quinoxalines against *S. mansoni* were also found in another recent study [49]. Therefore, we cannot exclude some challenges in translating *in vivo* efficacies of compounds 25, 30, 31, 32 and 33 despite demonstrating more potent *in vitro* activities when compared to existing anti-schistosomal quinoxaline analogues reported to date.

For this reason, we are currently working on improving the pharmacokinetic properties of these compounds as well as exploring different formulations and dosing regimens. A combination of these factors may facilitate better translation of their excellent *in vitro* activity to an *in vivo* murine model of schistosomiasis. Alongside this, we are confident that mechanism of action studies [52] would help identify the target of these compounds and perhaps lead to the further development of more potent and selective quinoxaline-containing, anti-schistosomal derivatives.

## 5 Experimental section

### 5.1 Chemistry

The starting material (2,3-Dichloro-6-nitroquinoxaline, product code 49489, purity 98%) and its hydroxy derivative (6-nitro-2,3-dihydroxyquinoxaline, product code 657239, purity 96%) were acquired from Fluorochem and Sigma, respectively. All solvents and reagents were used as supplied from Sigma-Aldrich, Fluorochem or other commercial sources without further purification or treatment. All reactions were monitored by Thin Layer Chromatography (TLC) on commercially available Merck Kieselgel 60 F254 plates (105554, Merck). TLC was performed with n-hexane/EtOAc (Ethyl Acetate) as the mobile phase; different proportions of the mobile phase constituents were used depending on the specific analogue being synthesised. Separated components were visualised using ultraviolet light (245 and 366 nm). The compounds were purified by flash column chromatography (using the eluents indicated) on an Interchim PuriFlash 430 using high performance silica gel particle size 50-μm cartridges. ^1^H and ^13^C were recorded on a Bruker Avance III HD spectrometer operating (500 and 125 MHz respectively, at 25 °C) in DMSO‑*d*_6_ solution. Spectra were calibrated to the residual signal of the deuterated solvent (*δ* = 2.50 and 39.52, for ^1^H and ^13^C NMR, respectively). Chemical shifts *δ* were given in parts per million (ppm) and rounded to two decimal places. The following abbreviations were used in the NMR signals assignment: s for singlet, br s for broad singlet, d for doublet, t for triplet, q for quartet, m for multiple. Coupling constants (*J*) were measured in Hertz (Hz) and rounded to one decimal place. The purity of synthesised compounds was determined by ultra-performance liquid chromatography-mass spectrometry (UPLC-UV-MS) analysis using a Waters UPLC system with both Diode Array detection and Electrospray (ESI)+ MS detection. The following conditions were applied: Waters Acquity UPLC BEH C18 1.7 μm 2.1 × 50 mm column, 0.5 ml/min, column temperature 40 °C; mobile phase was LC-MS grade H_2_O containing 0.1% formic acid (A) and LC-MS grade MeCN (acetonitrile) containing 0.1% formic acid (B); sample diluent: MeCN; sample concentration: 1 μg/ml; injection volume: 2 μl, gradient 90% eluent A (0.1 min), 90%–0% eluent A (1.5 min), 0% eluent A (1.4 min), 90% eluent A (0.1 min) (method 1). All compounds tested in the biological assays showed >95% purity. For each compound series (Series A, B and C), the general synthetic method is reported together with the characterisation of one representative compound. All other compound characterisations (UPLC-MS, ^1^H, ^13^C NMR) can be found in the Supplementary Information.

#### 5.1.1 Standard procedure for preparation of N-(CH_2_)_n_ aromatic quinoxaline analogues (Series A and B, compounds 22–38, n = 0, 1, 2)

To a stirring solution of purified 2,3-Dichloro-6-nitroquinoxaline (1 equivalent - 1 eq) in anhydrous DMSO under a nitrogen atmosphere, the appropriate aniline or phenyl-alkyl amine (5 eq) was added. The reaction mixture was left stirring at 130 °C for 30 min. The reaction was monitored by TLC with n-hexane/EtOAc (different proportions of the mobile phase constituents were used depending on the specific analogue being synthesised). After completion of the reaction, the mixture was diluted with EtOAc and poured into ice water. The aqueous phase was extracted with EtOAc (3 × 15 ml), the combined organic phase was then washed with 6 N hydrochloric acid (4 × 5 ml) and with brine (1 × 10 ml). The organic phase was then dried over anhydrous Na_2_SO_4_, filtered and reduced to dryness to give a crude product. The crude product was purified by flash chromatography and eluted with different proportions of n-hexane/EtOAc depending on the specific analogue being synthesised.

*N*^*2*^*, N*^*3*^*-bis(3,4-dichlorophenyl)-6-nitroquinoxaline-2,3-diamine (22):* red powder, 36% yield. ^1^H NMR (500 MHz, DMSO‑*d*_6_) *δ* 9.52 (s, 2H, 2 x N*H*), 8.34–8.18 (m, 3H, 3 x Ar*H*), 8.11 (d, *J* = 8.9 Hz, 1H, Ar*H*), 7.87 (m, 2H, 2 x Ar*H*), 7.65 (dd, *J* = 8.8, 6.9 Hz, 3H, 3 x Ar*H*); ^13^C NMR (125 MHz, DMSO) *δ* 139.65 (Ar*C*), 130.95 (Ar*C*), 130.57 (3 x Ar*C*H), 124.98 (Ar*C*), 124.50 (Ar*C*), 122.26 (Ar*C*H), 121.66 (Ar*C*H), 121.13 (Ar*C*H), 120.82 (Ar*C*H), 120.61 (Ar*C*H), 119.87 (Ar*C*H); UPLC-MS: Rt (Retention Time): 2.53 min, MS (ESI)^+^: 496.08 [M+H]^+^. The spectral data are in accordance with those reported in the literature [41,42,49].

*N*^*2*^*, N*^*3*^*-bis(3,4-dichlorophenyl)quinoxaline-2,3,6-triamine* (22a): A mixture of compound 22 (0.24 g, 0.48 mmol) and 10% Pd/C (0.25 g) was stirred under hydrogen atmosphere at room temperature in EtOAc (24 ml) for 8 h. After completion of the reaction, it was filtered through Celite. The filtrate was dried over anhydrous Na_2_SO_4_, filtered and reduced to dryness to give a crude product. The crude product was purified by automated flash column chromatography eluting with n-hexane:EtOAc 100:0 v/v increasing to 50:50 v/v in 15 CV. The final product 22a was obtained at 50% yield as a yellow powder. ^1^H NMR (500 MHz, DMSO‑*d*_6_): *δ* 9.06 (s, 1H, NH), 8.88 (s, 1H, N*H*), 8.36 (d, *J* = 2.5 Hz, 1H, Ar*H*), 8.14 (d, *J* = 2.6 Hz, 1H, Ar*H*), 7.78 (dd, *J* = 8.9, 2.6 Hz, 1H, Ar*H*), 7.70 (dd, *J* = 8.9, 2.6 Hz, 1H, Ar*H*), 7.61 (d, *J* = 8.8 Hz, 1H, Ar*H*), 7.56 (d, *J* = 8.8 Hz, 1H, Ar*H*), 7.34 (d, *J* = 8.7 Hz, 1H, Ar*H*), 6.83 (dd, *J* = 8.8, 2.5 Hz, 1H, Ar*H*), 6.72 (d, *J* = 2.4 Hz, 1H, Ar*H*), 5.42 (s, 2H, N*H*_2_); ^13^C NMR (125 MHz, DMSO) *δ* 147.98 (Ar*C*), 141.44 (Ar*C*), 140.98(Ar*C*), 140.83 (Ar*C*), 137.91(Ar*C*), 137.21 (Ar*C*), 130.76 (Ar*C*), 130.37 (2 x Ar*C*H), 128.38 (Ar*C*), 126.31 (Ar*C*H), 122.97 (Ar*C*), 122.12 (Ar*C*), 120.63 (Ar*C*H), 119.83 (Ar*C*H), 119.74 (Ar*C*H), 118.98 (Ar*C*H), 116.86 (Ar*C*H), 105.85 (Ar*C*H); UPLC-MS: Rt: 2.66 min, MS (ESI)^+^: 466.04 [M+H]^+^.

N^2^, N^3^-bis(3,4-dichlorophenyl)-6-(pyrrolidin-1-yl)quinoxaline-2,3-diamine (22b): To a mixture of compound 22b (0.03 g, 0.06 mmol, 1 eq) in CH_3_CN (2 ml), dibromobutane (0.03 g, 0.14 mmol, 2.2 eq) and anhydrous K_2_CO_3_ (0.01 g, 0.07 mmol, 1.1 eq) was added. The reaction mixture was heated at 150 °C for 15 min. After reaction completion, the mixture was filtered with EtOAc (20 ml). The resultant organic phase was washed with saturated aqueous NaHCO_3_ (3 × 5 ml) and brine (1 × 10 ml). The organic layer then dried over anhydrous Na_2_SO_4_, filtered and reduced to dryness to give a crude product. The crude product was purified by automated flash column chromatography eluting with n-hexane:EtOAc 100:0 v/v increasing to 80:20 v/v in 10 CV. The final product 22b was obtained at 28% yield as a brown powder. ^1^H NMR (500 MHz, DMSO‑*d*_6_): *δ* 9.09 (s, 1H, N*H*), 8.93 (s, 1H, N*H*), 8.23 (d, *J* = 2.6 Hz, 1H, Ar*H*), 8.15 (d, *J* = 2.5 Hz, 1H, Ar*H*), 7.90 (dd, *J* = 8.9, 2.5 Hz, 1H, Ar*H*), 7.75 (dd, *J* = 8.9, 2.5 Hz, 1H, Ar*H*), 7.63 (d, *J* = 8.8 Hz, 1H, Ar*H*), 7.58 (d, *J* = 8.8 Hz, 1H, Ar*H*), 7.47 (d, *J* = 8.9 Hz, 1H, Ar*H*), 6.90 (dd, *J* = 9.0, 2.6 Hz, 1H, Ar*H*), 6.57 (d, *J* = 2.6 Hz, 1H, Ar*H*), 3.35 (t, *J* = 6.6 Hz, 4H, 2 x C*H*_2_), 2.04–1.96 (m, 4H, 2 x C*H*_2_); ^13^C NMR (125 MHz, DMSO) *δ* 145.75 (Ar*C*), 141.34 (Ar*C*), 141.20 (Ar*C*), 140.63 (Ar*C*), 138.11 (Ar*C*), 136.89 (Ar*C*), 131.13 (Ar*C*), 131.04 (Ar*C*), 130.72 (Ar*C*H), 130.58 (Ar*C*H), 128.61(Ar*C*), 126.51 (Ar*C*H), 124.09 (Ar*C*), 122.91 (Ar*C*), 121.69 (Ar*C*H), 120.62 (Ar*C*H), 120.43 (Ar*C*H), 119.49 (Ar*C*H), 114.87 (Ar*C*H), 105.12 (Ar*C*H), 48.97 (2 x *C*H_2_), 25.01 (2 x *C*H_2_); UPLC-MS: Rt: 2.71 min, MS (ESI)^+^: 518.05 [M − H]^+^.

#### 5.1.2 Standard procedure for preparation of N-acyl derivatives of compound 22 (Series C, compounds 22c-22g)

To a solution of compound 22a (1 eq) dissolved in anhydrous DCM, anhydrous pyridine (3.6 eq) was added under nitrogen. The appropriate acyl derivate (1.1 eq) was added dropwise to the above solution cooled to 0 °C in an ice-bath under nitrogen atmosphere. The resulting mixture was stirred at room temperature for 1 h. After completion, the reaction mixture was diluted with DCM and quenched with saturated aqueous sodium bicarbonate (NaHCO_3_). The organic phase was washed first with saturated aqueous NaHCO_3_ (3 × 5 ml) and brine (1 × 10 ml). The organic layer was then dried over anhydrous Na_2_SO_4_, filtered and reduced to dryness to give a crude product. The crude product was purified by flash chromatography eluting with n-hexane/EtOAc in different proportions depending on the specific analogue being synthesised.

*N-(2,3-bis((3,4-dichlorophenyl)amino)quinoxalin-*6-yl*)acetamide (22c):* pale-yellow powder, 52% yield. ^1^H NMR (500 MHz, DMSO‑*d*_6_): *δ* 10.11 (s, 1H, N*H*–C=O), 9.23 (s, 1H, N*H*), 9.15 (s, 1H, N*H*), 8.31 (d, *J* = 2.5 Hz, 1H, Ar*H*), 8.24 (d, *J* = 2.6 Hz, 1H, Ar*H*), 8.00 (d, *J* = 2.1 Hz, 1H, Ar*H*), 7.81 (ddd, *J* = 9.3, 6.9, 2.5 Hz, 2H, 2 x Ar*H*), 7.63 (dd, *J* = 18.7, 8.8 Hz, 2H, 2 x Ar*H*), 7.58–7.50 (m, 2H, 2 x Ar*H*), 2.09 (s, 3H, CH_3_); ^13^C NMR (125 MHz, DMSO): *δ* 168.38 (*C*=O), 141.15 (Ar*C*), 140.64 (Ar*C*), 140.43 (Ar*C*), 139.82 (Ar*C*), 137.46 (Ar*C*), 136.30 (Ar*C*), 132.18 (Ar*C*), 130.79 (2 x Ar*C*), 130.47 (Ar*C*H), 130.43 (Ar*C*H), 125.78 (Ar*C*H), 123.52 (Ar*C*), 123.20 (Ar*C*), 121.04 (Ar*C*H), 120.78 (Ar*C*H), 120.05 (Ar*C*H), 119.81 (Ar*C*H), 118.78 (Ar*C*H), 113.96 (Ar*C*H), 24.12 (CH_3_); UPLC-MS: Rt: 2.19 min, MS (ESI)^+^: 508.02 [M+H]^+^. The spectral data are in accordance with those reported in the literature [49].

### 5.2 Biological evaluation

#### 5.2.1 Ethics statement

All procedures performed on mice (for provision of *S. mansoni*) adhered to the United Kingdom Home Office Animals (Scientific Procedures) Act of 1986 (project license PPL 40/3700 and P3B8C46FD) as well as the European Union Animals Directive 2010/63/EU and were approved by Aberystwyth University's Animal Welfare and Ethical Review Body (AWERB). The use of hamsters and mice (for provision of *S. haematobium* and *S. japonicum*, respectively) was approved by the Institutional Animal Care and Use Committee of the University of California San Diego.

#### 5.2.2 Compound handling

Prior to the biological assays, each compound was dissolved in dimethyl sulfoxide (DMSO, 276855, Sigma-Aldrich, UK) at a stock concentration of 10 mM and a working concentration of 1.6 mM (or lower, if required). Small aliquots of both stock and working solutions were stored at −20 °C prior to use. Similar preparations were followed for auranofin (AUR, A6733, Sigma-Aldrich, UK) and praziquantel (PZQ, P4668, Sigma-Aldrich, UK) positive controls used in the schistosomula and adult worm screens, respectively.

#### 5.2.3 Preparation of *S. mansoni* schistosomula for compound screening

Cercariae derived from the NMRI (National Medical Research Institute) Puerto Rican strain (PR-1) of *S. mansoni* were shed from two *Biomphalaria glabrata* strains, the NMRI albino and pigmented outbred strains, under intensified lighting conditions (1 h of incubation at 26 °C) [53]. Cercariae were then mechanically transformed into schistosomula as previously described [54].

High-throughput screening (HTS) of selected compounds was conducted as previously described [18,55]. Briefly, newly transformed schistosomula were distributed in a 384-well tissue culture plate (PerkinElmer, cat 6007460, seeding density of 120 parasites/well). Each compound (at a single concentration or two-fold titrations) was transferred into individual wells alongside the positive and negative controls (AUR at 10 μM final concentration in 0.625% DMSO and 0.625% DMSO, respectively). Schistosomula/compound co-cultures were then incubated at 37 °C for 72 h in a humidified atmosphere containing 5% CO_2_. Following the co-culture period, automatic assessment of compound-induced schistosomula motility and phenotypic changes were conducted with the high-content imaging platform Roboworm using the image analysis model previously described by Paveley *et al.* [56].

#### 5.2.4 S. mansoni juvenile and adult worm culture and *in vitro* compound screening

Mice (*Mus musculus* HsdOLa:TO - Tuck Ordinary; Envigo, UK) were used for the mammalian-specific propagation of the *S. mansoni* life cycle. Experimental animals were infected with 4000 or 180 *S. mansoni* cercariae/mouse and perfused three- or seven-weeks post-infection to obtain *S. mansoni* juvenile or adult worms, respectively [57]. Preparation steps following perfusion have been described previously [55]. Briefly, the parasite material was washed three times with pre-warmed adult worm media (DMEM (Gibco, Paisley, UK) supplemented with 10% (v/v) Fetal Calf Serum (FCS, Gibco, Paisley, UK), 1% (v/v) l-glutamine (Gibco, Paisley, UK) and an antibiotic mixture (150 Units/ml penicillin and 150 μg/ml streptomycin; Gibco, UK)) to remove residual host contamination [8] and incubated in a humidified environment containing 5% CO_2_ at 37 °C for at least 1 h.

Juvenile worms were distributed in a 96-well tissue culture plate (n = 20–25 individuals/well) containing 200 μl/well. Compounds were added to each treatment well at a concentration range of 10–0.156 μM. Negative (1.25% DMSO) and positive (15 μM PZQ and 15 μM AUR in 1.25% DMSO) controls were also included in each drug screen. Treated juvenile worms were incubated for 72 h in a humidified environment containing 5% CO_2_ at 37 °C. Compound-induced phenotype and motility effects were quantified using an adapted version of the WHO-TDR scoring system (in summary: 0 = dead, 1 = movement of the suckers only and slight contraction of the body, 2 = movement at the anterior and posterior regions only, 3 = full body movement but sluggish and 4 = normal movement) [58]. Additionally, differential uptake of propidium iodide (PI, P1304MP, Sigma-Aldrich, UK) was used to quantify worm viability as previously described [11,59]. In brief, after 15 min incubation with 2 μg/ml PI, the parasite cultures were imaged under both bright-field and fluorescent settings (PI detection, 562 and 624 excitation and emission wavelengths, respectively) using an ImageXpressXL high content imager (Molecular Devices, UK). Enumeration of PI positive and negative juvenile worms was manually performed across all parasites within the well and the percentage of PI positive parasite was calculated per well.

For the evaluation of anti-schistosomal activity on the adult stage of the parasite, 7-wk old worms were transferred into 48 well tissue culture plates (1–3 worm pairs/well, in duplicate). The worms were dosed with compounds at final concentrations spanning 0.78–50 μM (in 0.5% DMSO). DMSO (0.5%) and praziquantel (10 μM in 0.5% DMSO) were also included as negative and positive control treatments. Treated adult worms were incubated for 72 h in a humidified environment containing 5% CO_2_ at 37 °C. Parasite motility after compound treatment was assessed by a digital image processing-based system (WormassayGP2) [60], modified after Wormassay [61,62]. Following compound-parasite co-incubation, eggs were collected, fixed in formalin (10% v/v formaldehyde) and enumerated aiming to collect further information on any compound-induced effects on parasite fecundity and IVLE production.

#### *5.2.5* *S. mansoni* miracidia-sporocyst transformation and *in vitro* compound screening

Following hepatic portal vein perfusion of TO mice, experimentally infected 7 weeks previously, the mouse livers were collected to isolate parasite eggs. Briefly, the infected tissues were homogenised in double saline solution (1.7% w/v NaCl) using a Waring blender. The homogenates were passed through a 0.45 μm filter to retain egg material and the resulting filtered solution was collected in a volumetric flask. Miracidia hatching was induced by exposure of eggs to light in artificial river water (e.g. Lepple water) [18,63,64]. The resulting miracidial suspension was collected, washed with Chernin's balanced salt solution (CBSS) [64] and enumerated prior to being used for *in vitro* miracidia to sporocyst screens, as previously described [18]. Briefly, each well of a 24-well culture plate was loaded with 500 μl of CBSS and the relevant volume of compound added to each well to give the final concentration for the screen (usually a titration at 0.5, 2, 5, 10 and 50 μM was performed, unless differently stated). Each treatment was set up in duplicate and parasites cultured in CBSS with 1% DMSO were set up as negative controls. After 48 h incubation at 26 °C, dead, fully transformed and partially transformed miracidia were enumerated as previously described [18,[64], [65], [66]].

#### *5.2.6* *In vitro* compound screening of *S. haematobium* and *S. japonicum* adults

Adult *S. haematobium* worms (Egyptian isolate) were recovered from male Golden Syrian hamsters infected with 600 cercariae 20 weeks earlier. Adult *S. japonicum* worms (Philippine isolate) were recovered from female Swiss Webster mice infected with 100 cercariae five weeks earlier.

For each species, parasite recovery involved reverse perfusion of the hepatic portal and mesenteric system [67,68] using DMEM (Fisher Scientific). Parasites were washed six times with Basch 169 medium [69] supplemented with 100 U/ml penicillin and 100 μg/ml streptomycin before manually distributing approximately 5 males per well (on occasion 1 or 2 females might also be present) of a 24-well plate (Corning Costar 3524). The volume in each well was made up to approximately 1 ml using the same medium supplemented with 4% Fetal Bovine Serum (FBS, Gibco, Paisley, UK) and the worms were left to acclimate overnight at 37 °C in a 5% CO_2_ environment. The next morning, a final concentration of 100 nM compound (in 1 μL DMSO) was added to each well and the final volume made up to 2 ml with Basch 169 medium supplemented with antibiotics and FBS. Each compound was tested in triplicate (except for some instances tested in duplicate). Incubations were maintained at 37 °C in a 5% CO_2_ environment and phenotypic changes recorded at 5, 24, 48 and 72 h.

Using a Zeiss Axiovert A1 inverted microscope, the male parasite's phenotypic changes in shape, density and motility were recorded using a constrained nomenclature of simple descriptors [68,70,71]. To allow for the partially quantitative comparisons of compound effects, each descriptor was typically given a value of 1 and these were summed to generate a ‘severity score’ with a maximum value of 4. Descriptors for severe phenotypes, specifically, death, degeneracy or damage to the surface tegument, were given the maximum value of 4. The phenotypic characterisation of compound effects focused on male worms due to the greater range of their motility characteristics that include flexing, bending, stretching, sucker walking and adherence, compared to the more sedentary female worms. In addition to descriptors, WormAssay [61], as adapted for the schistosome, was used to measure average worm motility per well [72,73].

### 5.3 HepG2 cell culture and MTT assay

The Human Caucasian Hepatocyte Carcinoma (HepG2) cell line (85011430, Sigma Aldrich) was used to perform cytotoxicity tests of compounds used in this study. Initially the cells were handled under sterile conditions and grown at 37 °C in a humidified atmosphere containing 5% CO_2_. Cytotoxicity was evaluated by way of a 3-(4, 5- dimethylthiazol-2-yl)-2,5-diphenyl tetrazolium bromide (MTT) assay as previously described [8,11,74]. The cells were counted in a dual chamber counting slide using the BIORAD TC-10 Automated cell counter, seeded in a 96- well plate (Fisher Scientific, Loughborough, UK) at a density of 2 × 10^4^ cells/well and incubated in an atmosphere of 5% CO_2_ at 37 °C. Following a 24 h incubation, cells were incubated with the compounds at seven different concentrations (1, 10, 20, 50, 75, 100 and 200 μM) for a further 24 h in a CO_2_ incubator at 37 °C. Alternatively, when a lower seeding density (5 × 10^3^ cells/well) was used, cells were dosed at 24 h and incubated in a CO_2_ incubator at 37 °C for 72 h (the incubation time used for both schistosomula and adult worm screens). In both cases, control cell treatments included DMSO (1.25% v/v; negative control) and Triton X-100 (1% v/v, Sigma-Aldrich; positive control). Each compound was tested in triplicate and each experiment was performed either three (for 24 h timepoint) or two times (for 72 h timepoint). The absorbance readings (at 570 nm) of the soluble formazan crystals were recorded using PolarStar Omega plate reader (BMG-Labtech). Dose response curves were generated by non-linear regression analysis in GraphPad Prism 7.02 (GraphPad Software Inc., San Diego, CA, USA), after log concentration transformation and normalization of the absorbance readings of each concentration point. The cytotoxic concentration inhibiting 50% cell growth (CC_50_) was derived at 24 and 72 h. The latter was used to calculate the selectivity index (SI) of the compounds presented herein.

### 5.4 Genotoxicity assessments

The *in vitro* micronucleus (MN) assay was employed for the assessment of chromosome damage. Human, p53 competent, lymphoblastoid TK6 cells (Cat. No. 95111735) were used in this study and obtained from the European Collection of Authenticated Cell Cultures (ECACC) [75]). RPMI 1640 (Gibco, Paisley, UK) culture media supplemented with 1% penicillin/streptomycin (100 U/ml Penicillin and 100 μg/ml Streptomycin), 10% heat inactivated horse serum (Gibco, Paisley, UK), 1% l-glutamine (Gibco, Paisley, UK) and hygromycine-B was used for TK6 cell culture [76]. Cells were incubated at 37 °C in a humidified atmosphere of 5% (v/v) CO2. A preliminary cell toxicity assessment of compound 22 was performed to define the most suitable working concentration range (0.5, 2 and 4 μM, derived from dilutions in DMSO) based on Cell Count Relative Cell Growth (RCG) and toxicity not exceeding 70%.

Once cells reached confluency, sub-cultures were established. Approximately 2 × 10^5^ TK6 cells/ml were placed in a series of sterile vented tissue culture flasks (Fisher brand) and treated for 1.5–2 cell-cycle period (24 h) with no recovery with compound 22, the negative control (1% DMSO) or the positive controls (45.4 μM Methyl methanesulphonate (MMS) and 8.4 μM Carbendazim (Crbz) in DMSO; supplied from Sigma-Aldrich - CAS numbers 66-27-3 and 10605-21-7, respectively). All incubation steps occurred at 37 °C in a humidified atmosphere containing 5% CO_2_.

After the treatment period, cultures were centrifuged at 200×*g* for 5 min at room temperature, the supernatant was discarded, and the pellet re-suspended in pre-warmed phosphate-buffered saline (PBS). Subsequently, the PBS was removed via centrifugation at 200×*g* and the pellet was re-suspended in BD FACS™ lysis solution (CAS 349202) enabling cell fixation and membrane permeabilization. Post fixation, cells were centrifuged for 5 min at 200×*g*, supernatant discarded and subsequently stained for ɣH2AX (using Alexa Fluor® 488 Mouse anti-H2AX (pS139), Clone N1-431, Mouse BALB/c IgG1, κ, Cat. No. 560445, BD Biosciences) and p53 (using PE anti-p53 Antibody, Clone DO-7, Mouse IgG2b, Cat. No. 645805, BioLegend), as described [36]. Following that, cells were counter stained with DRAQ5™ (Cat. No. 564902, BD Biosciences) or left in PBS (as negative fluorescence controls). Sample incubation time for DRAQ5™ staining was a minimum of 20 min at room temperature under agitation. After all incubation periods were completed, samples were washed with PBS. Stained cells were then acquired on the ImageStreamX Mark II® imaging platform (the experiment was repeated three times).

For MNi assessment, of the 14,000–15,000 viable cells per experiment, about 4000 cell images were assessed except the positive control (Crbz) where 1600 cells were examined. Data analysis was performed as described [77]. Briefly, individual images of cell populations were exported as compensated image files (.cif) and the image files were then converted to grayscale, three 8-bit channel TIF files (nuclear fluorescence, brightfield and darkfield channels). The three individual channels were renormalised (max-min) and cropped to 64 × 64 pixel size. Automated scoring and classification of the TIF files was performed using the DeepFlow neural network. The deep neural network was previously trained on a ground truth image set representing a wide range of cell phenotypes that arose from TK6 cells treated with carbendazim and MMS as described in Wills et al. [77]. The TIF file images from this study were inputted into the trained DeepFlow convolutional neural network. TIF files of mononucleated cells and mononucleated cells with MN with a percentage confidence of 70% and above were extracted and MN response (expressed as %MNi) was calculated using the following formula: (mononucleated cells with MN/mononucleated cells) x 100.

The full 14,000–15,000 cell population was assessed for the biomarkers ɣH2AX and p53 alongside compound 22 autofluorescence. Gating parameters for ɣH2AX and p53 were determined based on the cell populations of unstained PBS controls and stained DMSO controls compared to positive control samples. These same gates were then applied for the batch processing of 22-treated cell populations.

Percentage of cells identified at each fluorescence channel were recorded in three independent experiments and data were then transformed as fold changes. A cut off for a negative vs a positive ɣH2AX and p53 response over the control is defined as a 1.5-fold change [30,37,39]. The MN response was considered positive based on a statistically significant response compared to that of DMSO control [78]. Where MN positive response was observed and biomarker response exceed fold change cut offs an indication of Mode of Action (MoA) may be inferred [36,37].

### 5.5 Laser scanning confocal microscopy on mammalian cells

To investigate the compound fluorescence emission, a preliminary lambda scan was performed on HeLa cells treated for 6 h with 5 μM of compound 22 in DMEM (Fisher Scientific) containing 10% FBS. Cells were washed three times in phenol red free DMEM and imaged using a Leica SP5 laser scanning confocal microscope.

Fluorescence intensity was measured following 405 nm excitation by sequentially imaging at 9 nm wavelength intervals between 420 nm and 780 nm using a 10 nm bandwidth. A final fluorescence readout was produced by quantifying the images produced in ImageJ.

HeLa, MDA-MB-231 and HepG2 cells were incubated with 5 μM of compound 22 in DMEM containing 10% serum for 60 min before washing and imaging in phenol red free DMEM by laser scanning confocal microscopy. Non-treated cells were also analysed as controls.

### 5.6 Statistics

Statistical analyses of parasitological assays were performed using a nonparametric two-way ANOVA followed by Least Significant Difference post-hoc correction (Kruskal-Wallis ANOVA followed by Dunn's multiple comparisons test). During genotoxicity studies, Bartlett and Shapiro-Wilk tests were used to confirm homogenous variance and normal distribution for p53 and ɣH2AX (*p* ≥ 0.05) datasets allowing for parametric data assessment using the Dunnett's test. All Statistical analyses were conducted using GraphPad Prism 7.02 (GraphPad Software Inc., San Diego, CA, USA).

## Author contributions

Conceived and designed the experiments: GP, AB, KFH. Performed the experiments: GP, MB, SF (compound synthesis and characterisation), GP (all *S. mansoni* screening and cell toxicity study), NE, LJL, CL (*S. japonicum* and *S. haematobium* screening), DSGH, REB (genotoxicity study), ES (confocal microscopy). Resources: JFT, HW (parasite material). Manuscript preparation: GP, KFH. Manuscript revision: GP, NE, LJL, CL, DSGH, JFT, HW, MB, SF, ATJ, CRC, IC, AB, KFH.

## Funding sources

KFH, GP and AB thank the Welsh Government, Life Sciences Research Network Wales scheme and the Wellcome Trust (107475/Z/15/Z) for financially supporting this project. The CDIPD team acknowledges the Bill and Melinda Gates Foundation (OPP1171488) for support. Hamsters and mice infected with *S. haematobium* and *S. japonicum*, respectively, were provided by the National Institute of Allergy and Infectious Diseases (NIAID) Schistosomiasis Resource Centre of the Biomedical Research Institute (Rockville, MD, USA) through the National Institutes of Health (NIH)-NIAID Contract HHSN272201700014I for distribution through BEI Resources.

## Declaration of competing interest

The authors declare that they have no known competing financial interests or personal relationships that could have appeared to influence the work reported in this paper.
