# Supplementary figures and images for: Anti-schistosomal activities of quinoxaline-containing compounds: From hit identification to lead optimisation
Source: Eur J Med Chem. 2021 Dec 15;226:113823. doi: 10.1016/j.ejmech.2021.113823 (PMC8626775; doi:10.1016/j.ejmech.2021.113823)

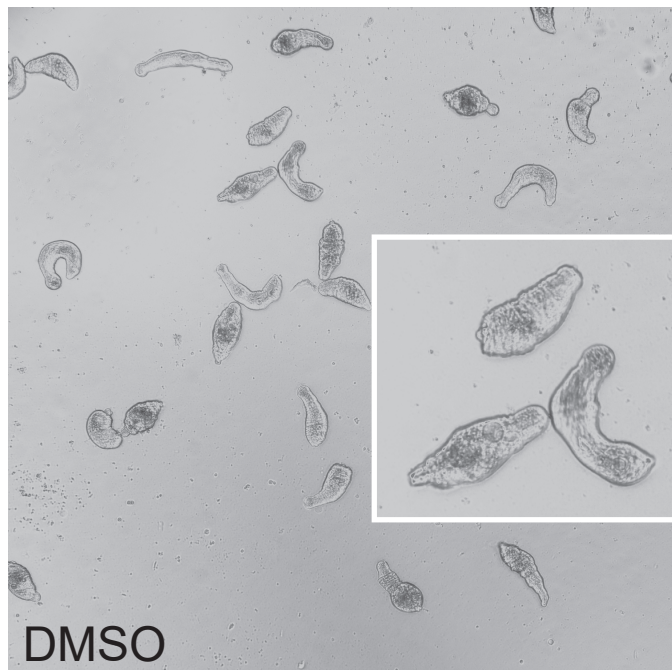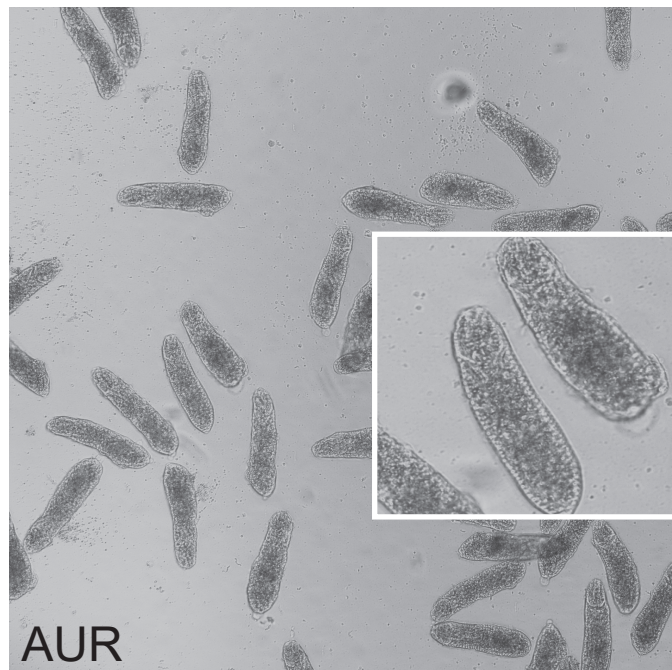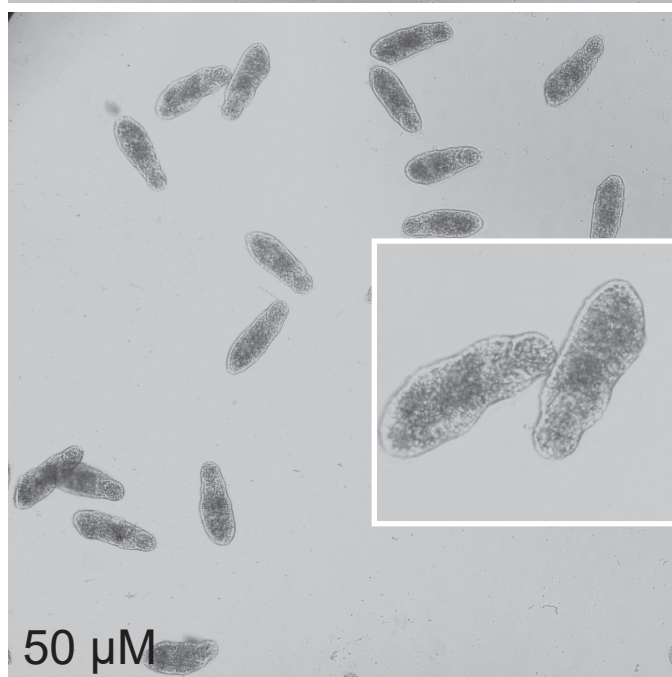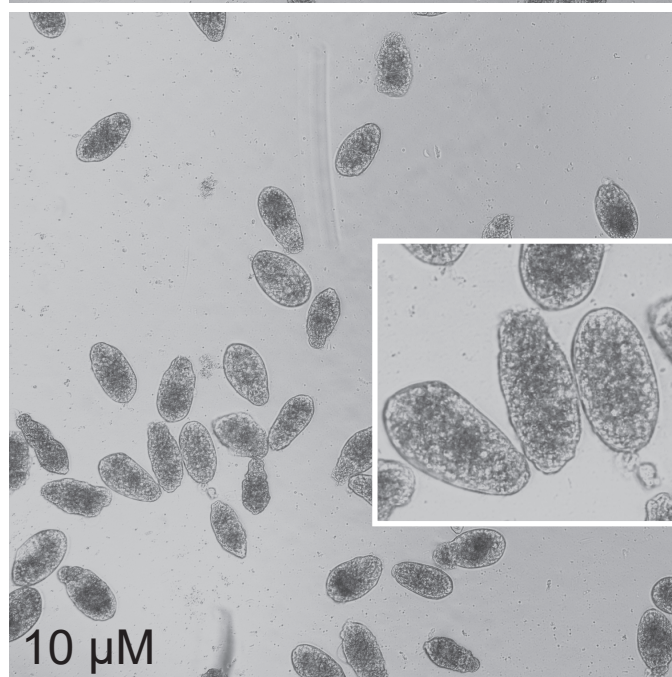

Supplement: Multimedia component 1 [file mmc1.pdf]

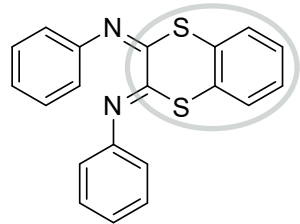

**1**

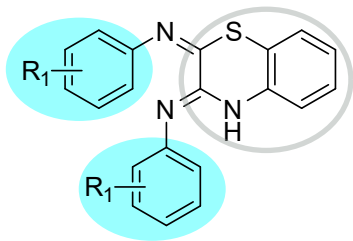

**Compounds 2, 3, 4**

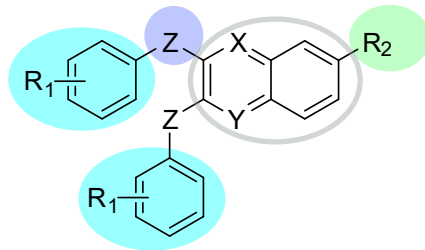

**Compounds 12, 14, 18, 21**

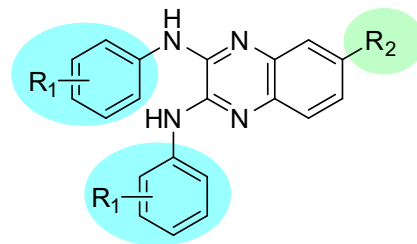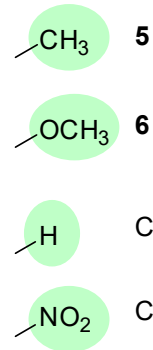

**5**

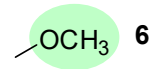

**6**

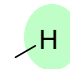

**Compounds 7-18**

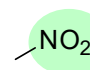

**Compounds 19-24**

Supplement: Multimedia component 2 [file mmc2.pdf]

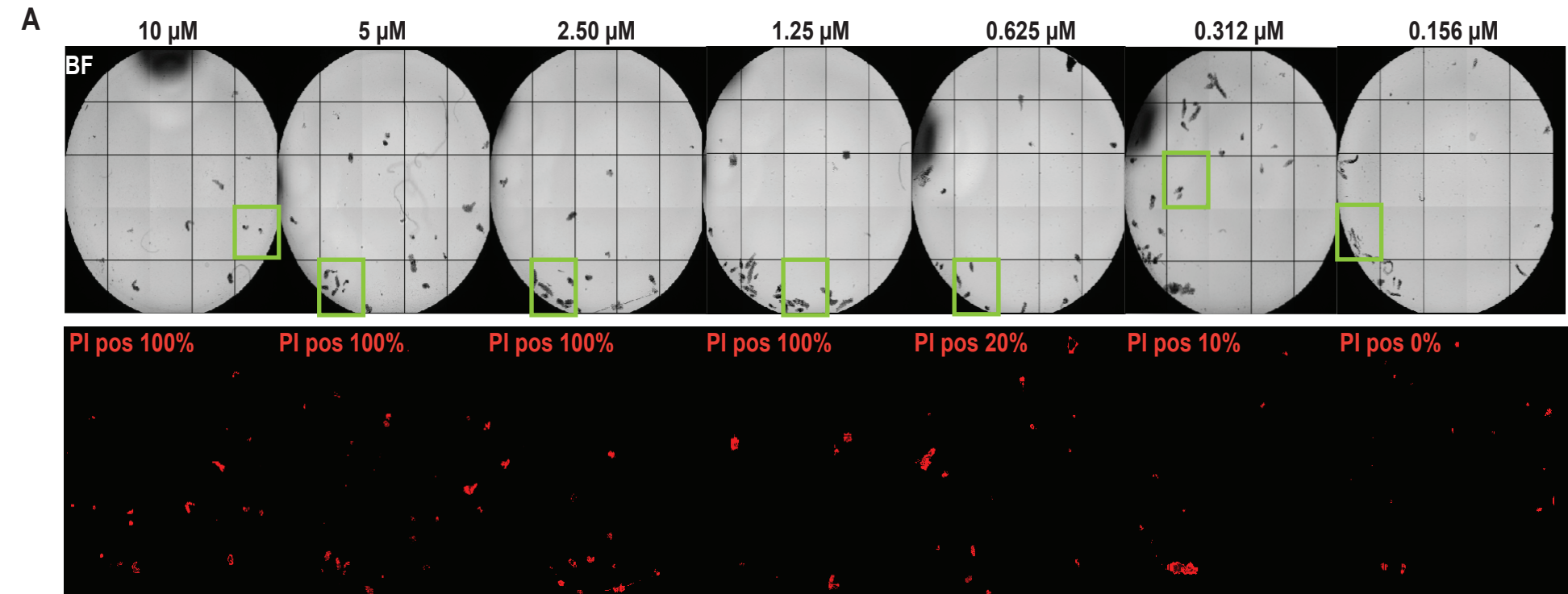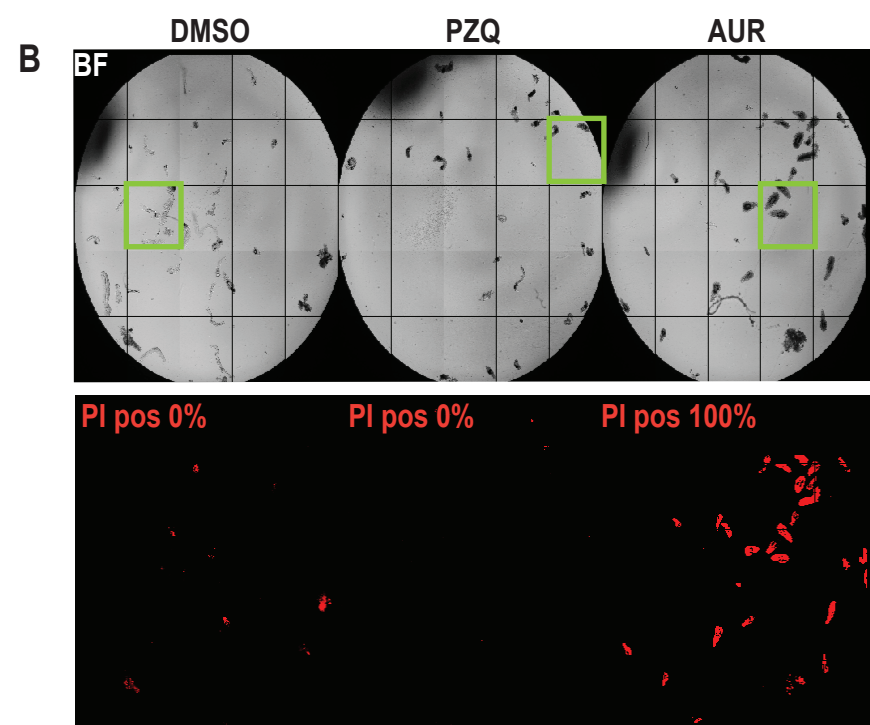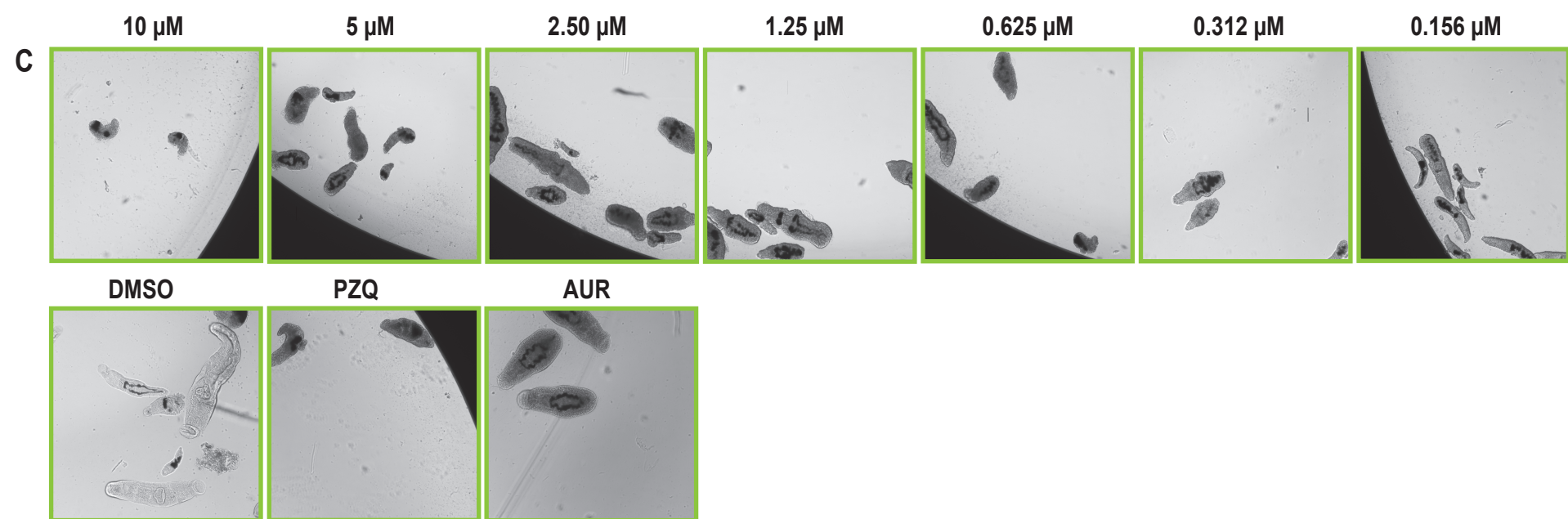

Supplement: Multimedia component 4 [file mmc4.pdf]

A

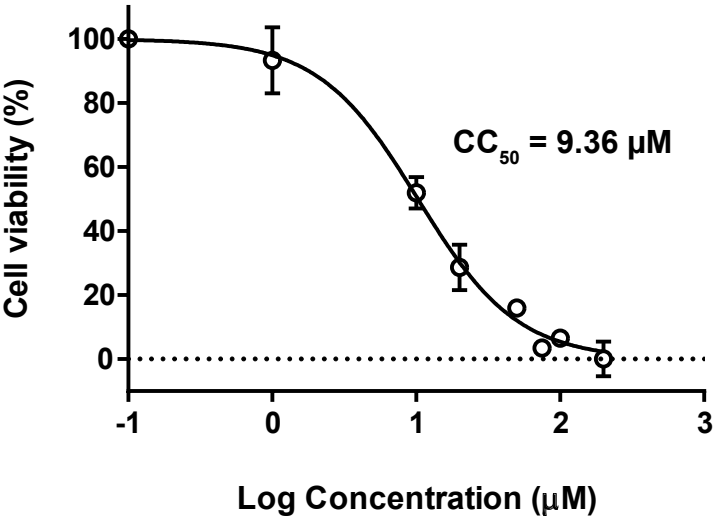

B

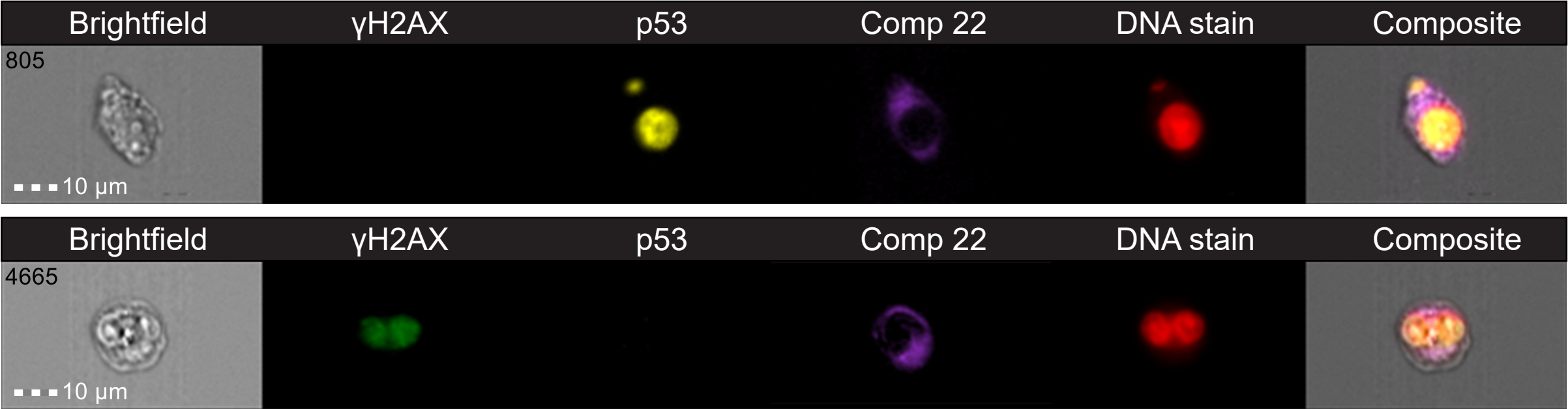

Supplement: Multimedia component 5 [file mmc5.pdf]

**A**

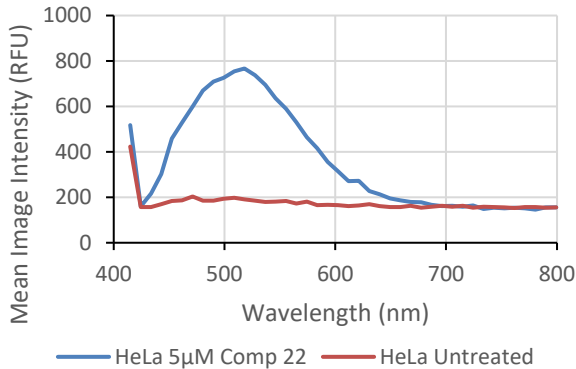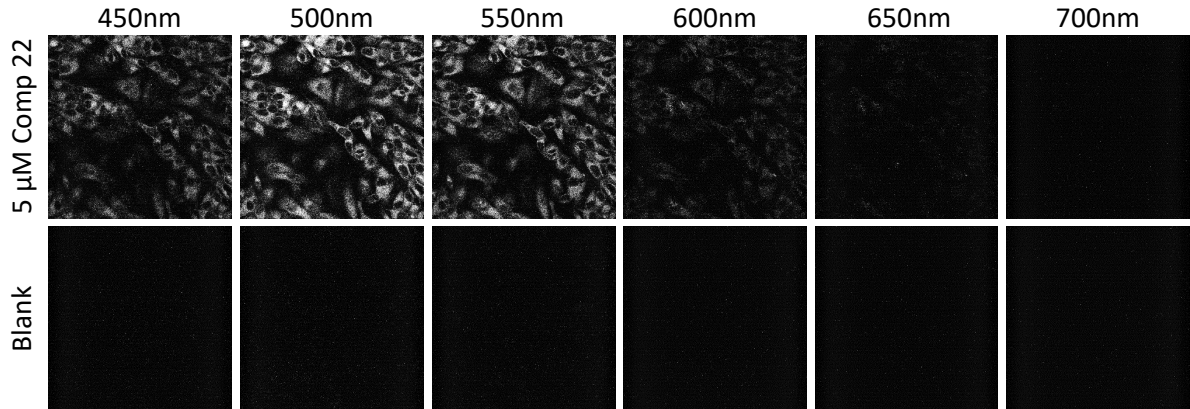

**B**

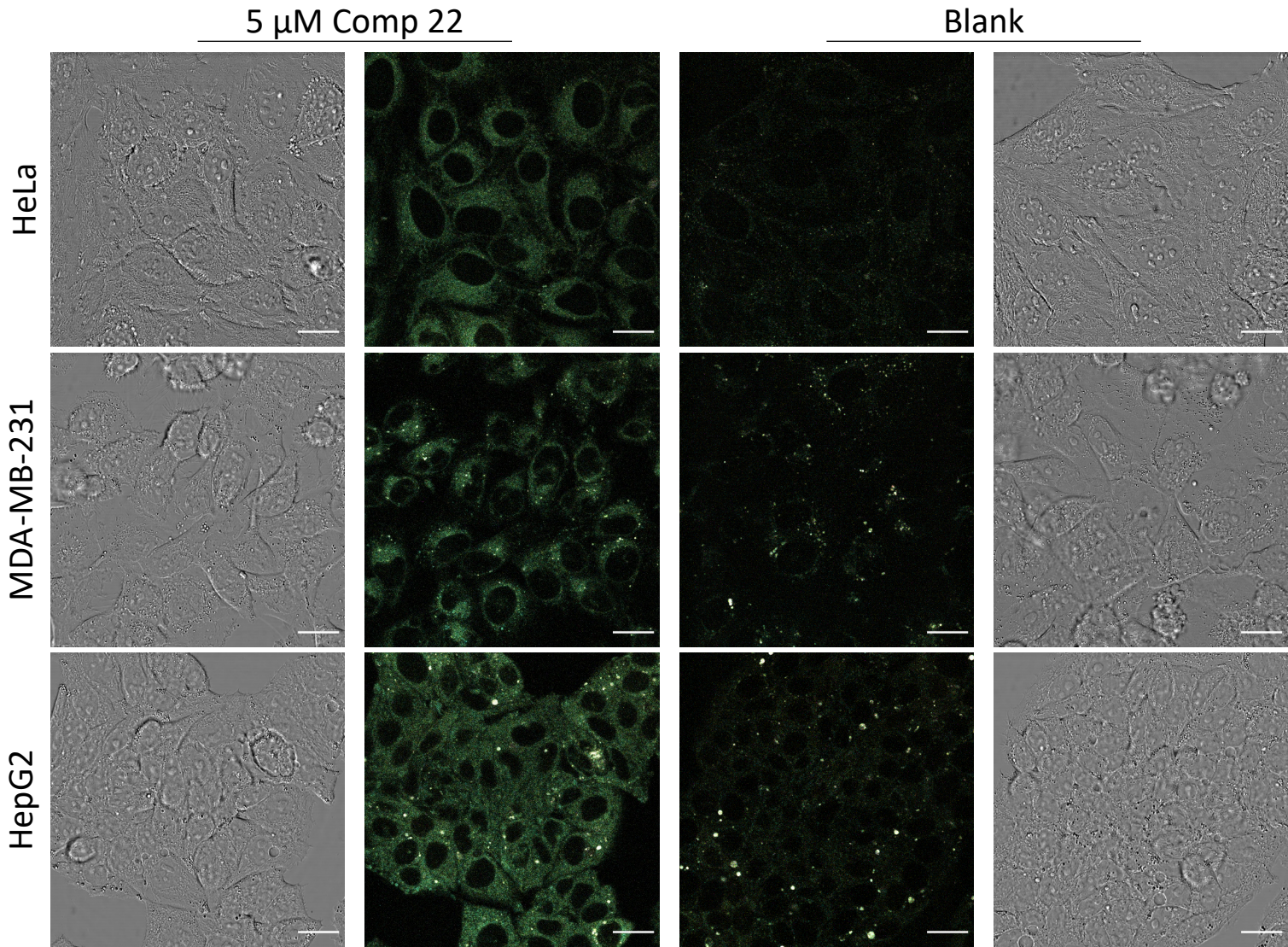

Supplement: Multimedia component 6 [file mmc6.pdf]

**A**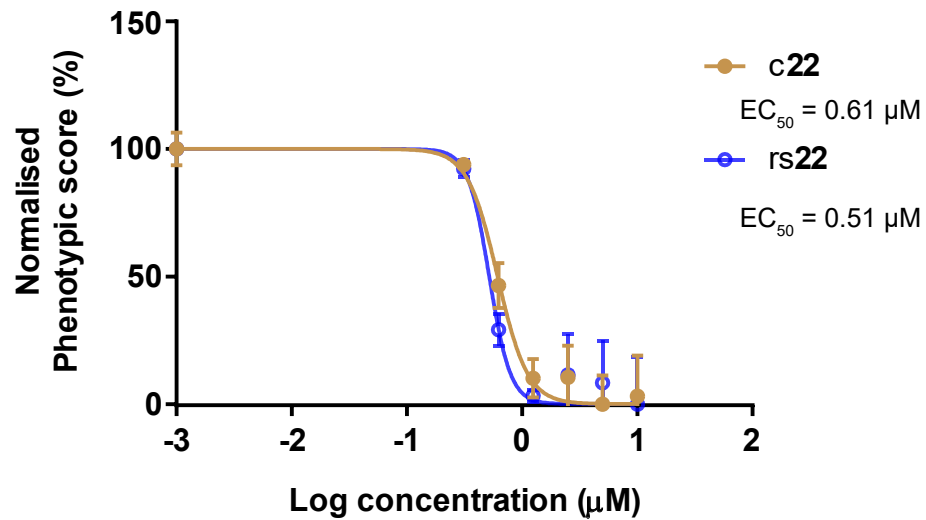**B**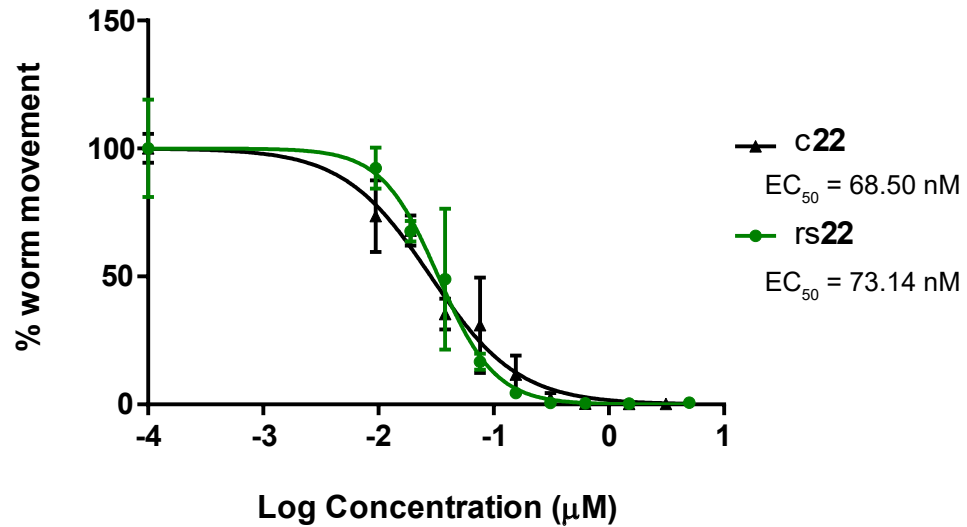

Supplement: Multimedia component 7 [file mmc7.pdf]
