# Supplementary material for: Anti-schistosomal activities of quinoxaline-containing compounds: From hit identification to lead optimisation
Source: Eur J Med Chem. 2021 Dec 15;226:113823. doi: 10.1016/j.ejmech.2021.113823 (PMC8626775; doi:10.1016/j.ejmech.2021.113823)
Supplement: Multimedia component 3 [file mmc3.pdf]

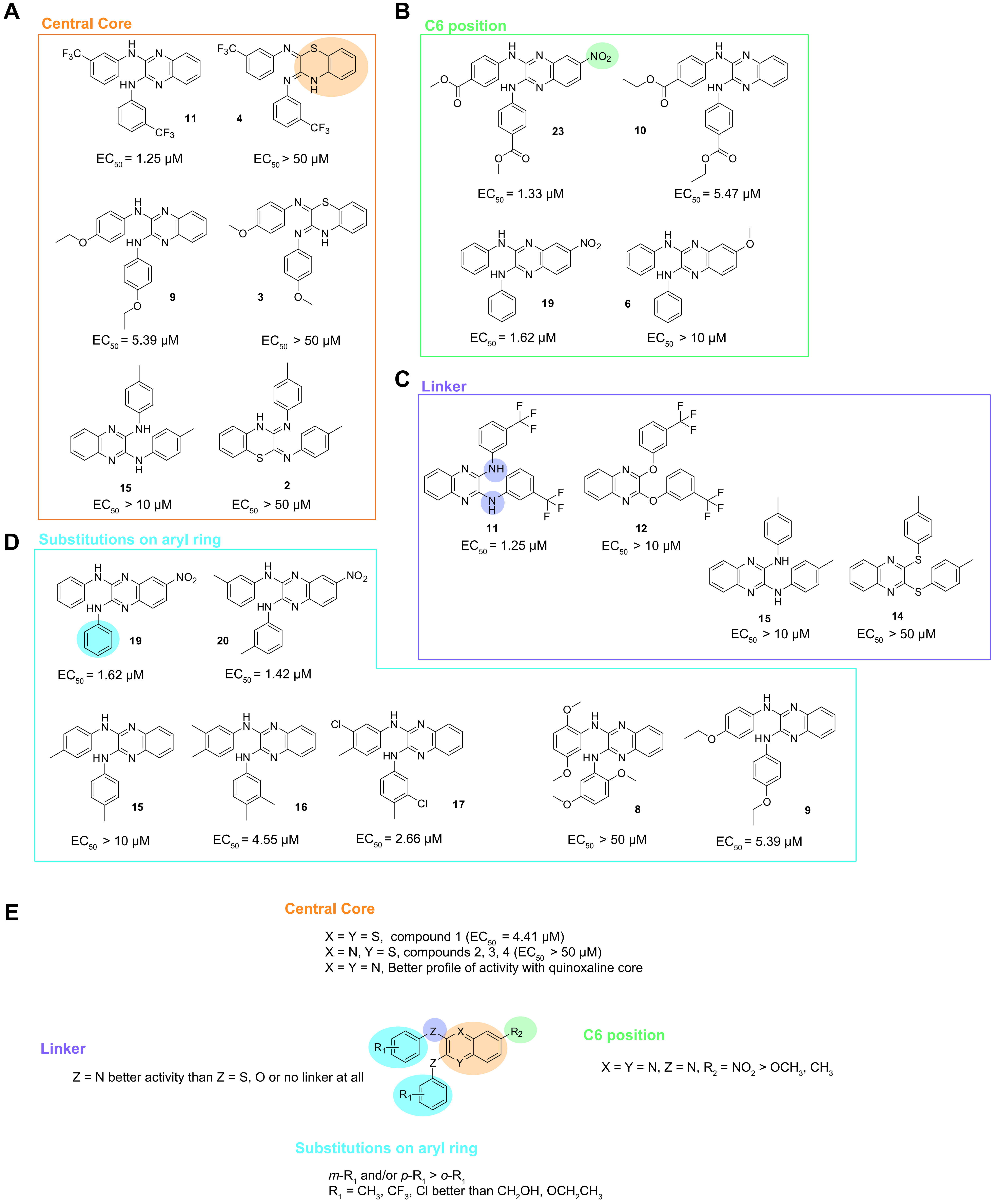
A
Central Core
11
EC50 = 1.25 μM
4
EC50 > 50 μM
9
EC50 = 5.39 μM
3
EC50 > 50 μM
15
EC50 > 10 μM
2
EC50 > 50 μM
B
C6 position
23
EC50 = 1.33 μM
10
EC50 = 5.47 μM
19
EC50 = 1.62 μM
6
EC50 > 10 μM
C
Linker
11
EC50 = 1.25 μM
12
EC50 > 10 μM
15
EC50 > 10 μM
14
EC50 > 50 μM
D
Substitutions on aryl ring
19
EC50 = 1.62 μM
20
EC50 = 1.42 μM
15
EC50 > 10 μM
16
EC50 = 4.55 μM
17
EC50 = 2.66 μM
8
EC50 > 50 μM
9
EC50 = 5.39 μM
E
Central Core
X = Y = S, compound 1 (EC50 = 4.41 μM)
X = N, Y = S, compounds 2, 3, 4 (EC50 > 50 μM)
X = Y = N, Better profile of activity with quinoxaline core
Linker
Z = N better activity than Z = S, O or no linker at all
C6 position
X = Y = N, Z = N, R2 = NO2 > OCH3, CH3
Substitutions on aryl ring
m-R1 and/or p-R1 > o-R1
R1 = CH3, CF3, Cl better than CH2OH, OCH2CH3
