# Supplementary material for: Anti-schistosomal activities of quinoxaline-containing compounds: From hit identification to lead optimisation
Source: Eur J Med Chem. 2021 Dec 15;226:113823. doi: 10.1016/j.ejmech.2021.113823 (PMC8626775; doi:10.1016/j.ejmech.2021.113823)
Supplement: Multimedia component 8 [file mmc8.pdf]

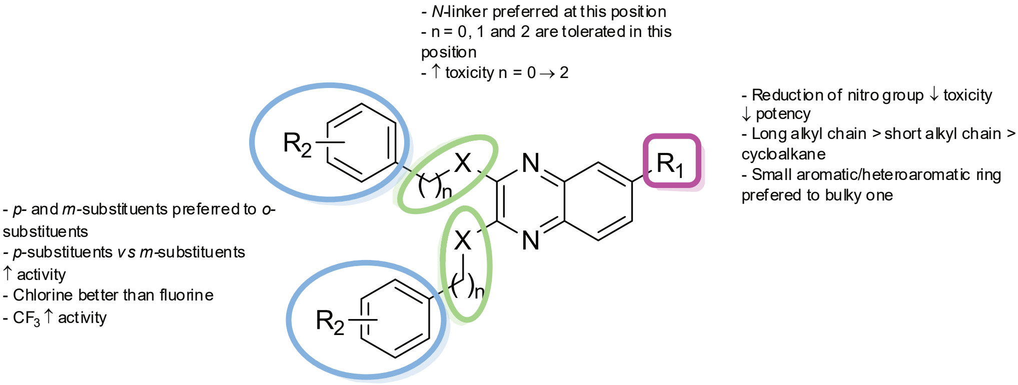

- *N*-linker preferred at this position
- n = 0, 1 and 2 are tolerated in this position
- ↑ toxicity n = 0 → 2
- Reduction of nitro group ↓ toxicity ↓ potency
- Long alkyl chain > short alkyl chain > cycloalkane
- Small aromatic/heteroaromatic ring prefered to bulky one
- *p*- and *m*-substituents preferred to *o*-substituents
- *p*-substituents *vs* *m*-substituents ↑ activity
- Chlorine better than fluorine
- CF_3 ↑ activity
R_2
X
n
N
N
R_1
X
n
R_2
