# Supplementary material for: Anti-schistosomal activities of quinoxaline-containing compounds: From hit identification to lead optimisation
Source: Eur J Med Chem. 2021 Dec 15;226:113823. doi: 10.1016/j.ejmech.2021.113823 (PMC8626775; doi:10.1016/j.ejmech.2021.113823)
Supplement: Multimedia component 10 [file mmc10.docx]

SUPPLEMENTARY INFORMATION

**Anti-schistosomal activities of quinoxaline-containing compounds: from hit identification to lead optimisation**

**Authors**

Gilda Padalino^1^, Nelly El-Sakkary^2^, Lawrence J. Liu^2^, Chenxi Liu^2^, Danielle S. G. Harte^3^, Rachel E. Barnes^3^, Edward Sayers^4^, Josephine Forde-Thomas^1^, Helen Whiteland^1^, Marcella Bassetto^5^, Salvatore Ferla^3^, George Johnson^3^, Arwyn T. Jones^4^, Conor R. Caffrey^2^, Iain Chalmers^1^, Andrea Brancale^4^, Karl F. Hoffmann^1^.

**Affiliations**

^1^Institute of Biological, Environmental and Rural Sciences (IBERS), Aberystwyth University, Aberystwyth, SY23 3DA, United Kingdom

^2^Center for Discovery and Innovation in Parasitic Diseases (CDIPD), Skaggs School of Pharmacy and Pharmaceutical Sciences, University of California, San Diego, La Jolla, CA 92093, USA

^3^Swansea University Medical School, Swansea University, Swansea, SA2 8PP, United Kingdom

^4^School of Pharmacy and Pharmaceutical Sciences, Cardiff University, Redwood Building, King Edward VII Avenue, Cardiff, CF10 3NB, United Kingdom

^5^Department of Chemistry, College of Science and Engineering, Swansea University, Swansea, SA2 8PP, United Kingdom

* Corresponding author:

KFH, e-mail: krh@aber.ac.uk

**UPLC-MS,** **^1^H-NMR and ^13^C-NMR chemical shifts and UPLC-MS of all the compounds**

N^2^, N^3^-bis(3-chlorophenyl)-6-nitroquinoxaline-2,3-diamine (**25**)

Yellow powder, 40% yield. ^1^H NMR (500 MHz, DMSO-*d*_6_) δ 9.57 (s, 1H, N*H*), 9.42 (s, 1H, N*H*), 8.31 (d, *J* = 2.7 Hz, 1H, Ar*H*), 8.17 – 8.03 (m, 3H, 3 x Ar*H*), 7.86 (dd, *J* = 23.1, 8.2 Hz, 2H, 2 x Ar*H*), 7.68 (d, *J* = 8.8 Hz, 1H, Ar*H*), 7.46 (td, *J* = 8.1, 2.5 Hz, 2H, 2 x Ar*H*), 7.20 (dd, *J* = 11.9, 8.5 Hz, 2H, 2 x Ar*H*); ^13^C NMR (125 MHz, DMSO) δ 144.04 (Ar*C*), 142.92 (Ar*C*), 142.20 (Ar*C*), 140.96 (Ar*C*), 140.82 (Ar*C*), 140.69 (Ar*C*),135.04 (Ar*C*Cl), 132.98 (Ar*C*Cl), 130.41 (3 x Ar*C*H), 126.22 (Ar*C*H), 123.31 (Ar*C*H), 122.88 (Ar*C*H), 120.74 (Ar*C*H), 120.64 (Ar*C*H), 120.09 (Ar*C*H), 119.64 (Ar*C*H), 119.13 (Ar*C*H); UPLC-MS: Rt (Retention Time): 2.78 min, MS (ESI)^+^: 426.20 [M]^+^. The spectral data are in accordance with those reported in the literature [1].

N^2^, N^3^-bis(4-chlorophenyl)-6-nitroquinoxaline-2,3-diamine (**26**)

Brown powder, 41% yield. ^1^H NMR (500 MHz, DMSO-d6) δ 9.57 (s, 1H, NH), 9.41 (s, 1H, NH), 8.34 – 8.27 (m, 1H, ArH), 8.10 (dd, J = 7.9, 4.2 Hz, 1H, ArH), 7.95 (ddd, J = 13.9, 8.7, 1.9 Hz, 4H, ArH), 7.64 (dd, J = 9.4, 4.1 Hz, 1H, ArH), 7.48 (ddd, J = 8.9, 4.0, 1.4 Hz, 4H, ArH); ^13^C NMR (125 MHz, DMSO) δ 144.39 (ArC), 143.41(ArC), 142.70 (ArC), 141.40 (ArC), 138.87 (ArC), 138.54 (ArC), 135.59 (ArC), 129.06 (4 x ArCH), 127.89 (ArCCl), 127.36 (ArCCl), 126.50 (ArCH), 123.49 (2 x ArCH), 122.96 (2 x ArCH), 121.12 (ArCH), 119.84 (ArCH); UPLC-MS: Rt: 2.76 min, MS (ESI)^+^: 426.18 [M]^+^. The spectral data are in accordance with those reported in the literature [1].

N_2_, N_3_-bis(2,3-dimethylphenyl)-6-nitroquinoxaline-2,3-diamine (**27**)

Yellow powder, 60% yield. ^1^H NMR (500 MHz, DMSO-*d*_6_) δ 9.17 (s, 1H, N*H*), 8.94 (s, 1H, N*H*), 8.03 (d, *J* = 2.6 Hz, 1H, Ar*H*), 7.98 – 7.84 (m, 1H, Ar*H*), 7.41 (d, *J* = 9.0 Hz, 1H, Ar*H*), 7.29 (s, 2H, 2 x Ar*H*), 7.18 (dt, *J* = 12.0, 8.1 Hz, 4H, 4 x Ar*H*), 2.33 (s, 6H, 2 x CH_3_), 2.13 (s, 6H, 2 x CH_3_); ^13^C NMR (125 MHz, DMSO) δ 144.54 (Ar*C*), 143.80 (Ar*C*), 143.08 (Ar*C*), 141.79 (Ar*C*), 137.42 (Ar*C*), 136.79 (Ar*C*), 136.54 (Ar*C*), 135.61 (Ar*C*), 132.80 (Ar*C*), 132.68 (Ar*C*), 127.80 (Ar*C*H), 127.54 (Ar*C*H), 125.64(Ar*C*H), 125.59 (Ar*C*H), 125.53 (Ar*C*H), 124.71 (Ar*C*H), 124.48 (Ar*C*H), 120.12 (Ar*C*H), 118.47 (Ar*C*H), 20.23 (2 x ArCH_3_), 14.56 (2 x ArCH_3_); UPLC-MS: Rt: 2.76 min, MS (ESI)^+^: 414.37 [M+H]^+^.

N_2_, N_3_-bis(4-fluorophenyl)-6-nitroquinoxaline-2,3-diamine (**28**)

Orange powder, 10% yield. ^1^H NMR (500 MHz, DMSO-*d*_6_) δ 9.56 (s, 1H, N*H*), 9.39 (s, 1H, N*H*), 8.29 (d, *J* = 2.6 Hz, 1H, Ar*H*), 8.19 (dd, *J* = 9.1, 5.0 Hz, 1H, Ar*H*), 8.10 (d, *J* = 8.9 Hz, 1H, Ar*H*), 7.94 (dd, *J* = 16.4, 10.6 Hz, 3H, 3 x Ar*H*), 7.67 – 7.60 (m, 1H, Ar*H*), 7.29 (td, *J* = 8.6, 4.3 Hz, 4H, 4 x Ar*H*); ^13^C NMR (125 MHz, DMSO) δ 176.35 (2 x Ar*C*), 143.88 (Ar*C*), 143.35 (Ar*C*), 142.61 (Ar*C*), 141.32 (Ar*C*), 135.84 (Ar*C*), 135.83 (Ar*C*), 135.37 (Ar*C*), 126.11 (Ar*C*H), 123.86 (Ar*C*H), 123.79 (Ar*C*H), 123.26 (Ar*C*H), 123.20 (Ar*C*H), 120.69 (Ar*C*H), 119.43 (Ar*C*H), 115.71 (Ar*C*H), 115.67 (Ar*C*H), 115.53 (Ar*C*H), 115.49 (Ar*C*H); UPLC-MS: Rt: 2.56 min, MS (ESI)^+^: 394.24 [M+H]^+^. The spectral data are in accordance with those reported in the literature [2].

N_2_, N_3_-bis(4-bromophenyl)-6-nitroquinoxaline-2,3-diamine (**29**)

Red powder, 60% yield. ^1^H NMR (500 MHz, DMSO-*d*_6_) δ 9.59 (s, 1H, N*H*), 9.44 (s, 1H, N*H*), 8.34 (d, J = 2.6 Hz, 1H, Ar*H*), 8.13 (dd, J = 9.1, 2.6 Hz, 1H, Ar*H*), 7.91 (dd, J = 15.5, 8.5 Hz, 3H, 3 x Ar*H*), 7.67 (d, J = 8.9 Hz, 1H, Ar*H*), 7.62 (dd, J = 8.7, 4.1 Hz, 3H, 3 x Ar*H*), 7.41 - 7.39 (m, 1H, Ar*H*), 7.36 - 7.34 (m, 1H, Ar*H*). The spectral data are in accordance with those reported in the literature [1, 3].

6-nitro-N_2_, N_3_-bis(3-(trifluoromethyl)phenyl)quinoxaline-2,3-diamine (**30**)

Yellow powder, 30% yield. ^1^H NMR (500 MHz, DMSO-*d*_6_) δ 9.74 (s, 1H, N*H*), 9.60 (s, 1H, N*H*), 8.36 – 8.21 (m, 5H, 5 x Ar*H*), 8.15 (dd, *J* = 9.0, 2.7 Hz, 1H, Ar*H*), 7.73 – 7.62 (m, 3H, 3 x Ar*H*), 7.49 (dd, *J* = 12.8, 7.9 Hz, 2H, 2 x Ar*H*); ^13^C NMR (125 MHz, DMSO) δ 144.16 (Ar*C*), 142.99 (Ar*C*), 142.27 (Ar*C*), 140.77 (Ar*C*), 140.29 (Ar*C*), 140.02 (Ar*C*), 135.04 (Ar*C*), 130.01 (Ar*C*H), 129.97 (Ar*C*H), 129.53 (Ar*C*), 126.26 (Ar*C*H), 125.32 (Ar*C*), 124.64 (Ar*C*H), 124.18(Ar*C*H), 120.76 (Ar*C*H), 119.86 (Ar*C*H), 119.79 (Ar*C*H), 119.42 (Ar*C*H), 117.28 (Ar*C*H), 116.75 (Ar*C*H); UPLC-MS: Rt: 2.34 min, MS (ESI)^+^: 494.15 [M+H]^+^. The spectral data are in accordance with those reported in the literature [1, 2].

6-nitro-N_2_, N_3_-bis(4-(trifluoromethyl)phenyl)quinoxaline-2,3-diamine (**31**)

Yellow powder, 50% yield. ^1^H NMR (500 MHz, DMSO-*d*_6_) δ 9.82 (s, 1H, N*H*), 9.69 (s, 1H, N*H*), 8.41 (d, *J* = 2.6 Hz, 1H, Ar*H*), 8.22 – 8.09 (m, 5H, 5 x Ar*H*), 7.80 (dd, *J* = 8.3, 3.3 Hz, 4H, 4 x Ar*H*), 7.74 (d, *J* = 9.0 Hz, 1H, Ar*H*); ^13^C NMR (125 MHz, DMSO) δ 144.22 (Ar*C*), 143.16 (Ar*C*), 142.87 (Ar*C*), 142.79 (Ar*C*), 142.07 (Ar*C*), 140.67 (Ar*C*), 135.03 (Ar*C*), 125.90 (5 x Ar*C*H), 123.06 (Ar*C*F_3_), 122.81 (Ar*C*F_3_), 120.94 (3 x Ar*C*H), 120.42 (2 x Ar*C*H), 119.78 (Ar*C*H); UPLC-MS: Rt: 2.36 min, MS (ESI)^+^: 494.13 [M+H]^+^. The spectral data are in accordance with those reported in the literature [2].

N_2_, N_3_-bis(4-fluoro-3-(trifluoromethyl)phenyl)-6-nitroquinoxaline-2,3-diamine (**32**)

Red powder, 60% yield. ^1^H NMR (500 MHz, DMSO-*d*_6_) δ 9.61 (s, 2H, 2 x N*H*), 8.32 (br s, 2H, 2 x Ar*H*), 8.23 (d, *J* = 13.8 Hz, 3H, 3 x Ar*H*), 8.10 (br s, 1H, Ar*H*), 7.57 (ddd, *J* = 17.4, 11.5, 7.0 Hz, 3H, 3 x Ar*H*); ^13^C NMR (125 MHz, DMSO) δ 174.16 (Ar*C*), 144.17 (Ar*C*), 142.96 (Ar*C*), 142.25 (Ar*C*), 140.72 (Ar*C*), 136.27 (Ar*C*), 135.96 (Ar*C*), 135.04 (Ar*C*), 127.22 (Ar*C*H), 126.74 (Ar*C*H), 126.26 (Ar*C*H), 124.73 (Ar*C*), 123.71 (Ar*C*), 120.74 (Ar*C*H), 119.81 (Ar*C*H), 119.18 (Ar*C*H), 118.62 (Ar*C*H), 117.80 (Ar*C*H), 117.63 (Ar*C*H); UPLC-MS: Rt: 2.80 min, MS (ESI)^+^: 530.08 [M+H]^+^.

N_2_, N_3_-bis(2-fluoro-5-(trifluoromethyl)phenyl)-6-nitroquinoxaline-2,3-diamine (**33**)

Yellow powder, 10% yield. ^1^H NMR (500 MHz, DMSO-*d*_6_) δ 9.63 (br s, 2H, 2 x N*H*), 7.67 – 7.48 (m, 4H, 4 x Ar*H*), 7.26 (dd, *J* = 11.6, 8.3 Hz, 1H, Ar*H*), 7.12 (dd, *J* = 20.4, 10.5 Hz, 1H, Ar*H*), 6.93 – 6.86 (m, 1H, Ar*H*), 6.74 – 6.67 (m, 1H, Ar*H*), 6.63 (dd, *J* = 8.2, 1.8 Hz, 1H, Ar*H*); ^13^C NMR (125 MHz, DMSO) δ 157.91 (Ar*C*), 153.77 (Ar*C*), 143.90 (Ar*C*), 135.84 (Ar*C*), 132.90 (Ar*C*H), 131.10 (Ar*C*H), 128.72 (Ar*C*H), 125.29 (Ar*C*), 124.53 (Ar*C*H), 122.03 (Ar*C*H), 118.78 (Ar*C*), 115.49 (Ar*C*H), 115.33 (Ar*C*H), 115.19 (Ar*C*H), 115.03 (Ar*C*H), 114.93 (Ar*C*), 111.95 (Ar*C*); UPLC-MS: Rt: 2.38 min, MS (ESI)^+^: 530.05 [M+H]^+^. The spectral data are in accordance with those reported in the literature [2].

N_2_, N_3_-bis(3-fluoro-5-(trifluoromethyl)phenyl)-6-nitroquinoxaline-2,3-diamine (**34**)

Orange powder, 10% yield. ^1^H NMR (500 MHz, DMSO-*d*_6_) δ 9.71 (br s, 2H, 2 x N*H*), 8.34 – 8.18 (m, 3H, 3 x Ar*H*), 8.10 (d, *J* = 8.9 Hz, 1H, Ar*H*), 7.92 (d, *J* = 31.7 Hz, 2H, 2 x Ar*H*), 7.65 (d, *J* = 9.0 Hz, 1H, Ar*H*), 7.33 (dd, *J* = 24.4, 8.3 Hz, 2H, 2 x Ar*H*); ^13^C NMR (125 MHz, DMSO) δ 163.17 (Ar*C*), 161.24 (Ar*C*), 147.21 (Ar*C*), 144.53 (Ar*C*), 142.61 (Ar*C*), 141.94 (Ar*C*), 140.41 (Ar*C*), 134.88 (Ar*C*), 131.00 (Ar*C*), 126.63 (Ar*C*H), 124.55 (Ar*C*), 122.38 (Ar*C*), 121.11 (Ar*C*H), 120.20 (Ar*C*H), 113.19 (Ar*C*H), 112.71 (Ar*C*H), 111.02 (Ar*C*H), 110.82 (Ar*C*H), 110.58 (Ar*C*H), 110.36 (Ar*C*H); UPLC-MS: Rt: 2.88 min, MS (ESI)^+^: 529.98 [M]^+^. The spectral data are in accordance with those reported in the literature [2].

N_2_, N_3_-bis(4-methoxy-3-(trifluoromethyl)phenyl)-6-nitroquinoxaline-2,3-diamine (**35**)

Red powder, 88% yield. ^1^H NMR (500 MHz, DMSO-*d*_6_) δ 9.50 (s, 1H, N*H*), 9.34 (s, 1H, N*H*), 8.24 – 8.06 (m, 6H, 6 x Ar*H*), 7.55 (d, *J* = 8.8 Hz, 1H, Ar*H*), 7.35 (d, *J* = 9.1 Hz, 2H, 2 x Ar*H*), 3.92 (s, 3H, C*H*_3_), 3.91 (s, 3H, C*H*_3_); ^13^C NMR (125 MHz, DMSO) δ 153.16 (Ar*C*-OCH_3_), 152.86 (Ar*C*-OCH_3_), 143.68 (Ar*C*), 142.99 (Ar*C*), 142.31 (Ar*C*), 141.01 (Ar*C*), 135.07 (Ar*C*), 132.16 (Ar*C*), 131.83 (Ar*C*), 126.92 (Ar*C*H), 126.53 (Ar*C*H), 125.80 (Ar*C*H), 124.70 (Ar*C*F_3_), 122.57 (Ar*C*F_3_), 120.35 (Ar*C*H), 119.96 (Ar*C*H), 119.44 (Ar*C*H), 119.29 (Ar*C*H), 116.73 (Ar*C*), 116.50 (Ar*C*), 113.37 (Ar*C*H), 113.36 (Ar*C*H), 56.28 (2 x *C*H_3_); UPLC-MS: Rt: 2.27 min, MS (ESI)^+^: 554.13 [M+H]^+^.

N_2_, N_3_-dibenzyl-6-nitroquinoxaline-2,3-diamine (**36**)

Orange powder, 65% yield. ^1^H NMR (500 MHz, DMSO-*d*_6_) δ 8.16 (d, *J* = 2.6 Hz, 1H, Ar*H*), 8.09 (t, *J* = 5.2 Hz, 1H, N*H*), 7.97 (dd, *J* = 8.9, 2.7 Hz, 1H, Ar*H*), 7.85 (t, *J* = 5.2 Hz, 1H, N*H*), 7.51 (d, *J* = 8.9 Hz, 1H, Ar*H*), 7.46 – 7.39 (m, 4H, Ar*H*), 7.36 (t, *J* = 7.6 Hz, 4H, Ar*H*), 7.30 – 7.25 (m, 2H, Ar*H*), 4.76 (d, *J* = 5.2 Hz, 2H, C*H*_2_), 4.73 (d, *J* = 5.1 Hz, 2H, C*H*_2_); ^13^C NMR (125 MHz, DMSO) δ 145.08 (Ar*C*), 144.39 (Ar*C*), 142.59 (Ar*C*), 141.92 (Ar*C*), 138.68 (Ar*C*), 138.42 (Ar*C*), 135.58 (Ar*C*), 128.43 (2 x Ar*C*H), 128.40 (2 x Ar*C*H), 128.13 (2 x Ar*C*H), 128.12 (2 x Ar*C*H), 127.20 (Ar*C*H), 127.13 (Ar*C*H), 125.04 (Ar*C*H), 119.77 (Ar*C*H), 118.01 (Ar*C*H), 44.50 (2 x *C*H_2_); UPLC-MS: Rt: 2.19 min, MS (ESI)^+^: 386.32 [M+H]^+^.

6-nitro- N_2_, N_3_- diphenethylquinoxaline-2,3-diamine (**37**)

Red powder, 75% yield. ^1^H NMR (500 MHz, DMSO-*d*_6_) δ 8.18 (d, *J* = 2.7 Hz, 1H, Ar*H*), 7.97 (dd, *J* = 8.9, 2.7 Hz, 1H, Ar*H*), 7.67 (t, *J* = 5.2 Hz, 1H, N*H*), 7.52 (d, *J* = 8.9 Hz, 1H, Ar*H*), 7.43 (t, *J* = 5.1 Hz, 1H, N*H*), 7.34 – 7.26 (m, 8H, 8 x Ar*H*), 7.25 – 7.18 (m, 2H, 2x Ar*H*), 3.73 (td, *J* = 12.6, 6.6 Hz, 4H, 2 x C*H*_2_-N), 2.97 (dd, *J* = 8.1, 6.6 Hz, 4H, 2 x C*H*_2_); ^13^C NMR (125 MHz, DMSO) δ 145.18 (Ar*C*), 144.48 (Ar*C*), 142.48 (Ar*C*), 141.96 (Ar*C*), 139.58 (Ar*C*), 139.46 (Ar*C*), 135.58 (Ar*C*), 128.74(2 x Ar*C*H), 128.73 (2 x Ar*C*H), 128.39 (2 x Ar*C*H), 128.38 (2 x Ar*C*H), 126.20 (Ar*C*H), 126.16 (Ar*C*H), 125.04 (Ar*C*H), 119.83 (Ar*C*H), 117.87 (Ar*C*H), 42.61 (*C*H_2_), 42.57 (*C*H_2_), 34.20 (*C*H_2_), 34.15 (*C*H_2_); UPLC-MS: Rt: 2.27 min, MS (ESI)^+^: 414.35 [M+H]^+^.

N_2_, N_3_-bis(4-bromophenyl)quinoxaline-2,3-diamine (**38**)

Brown powder, 58% yield. ^1^H NMR (500 MHz, DMSO-*d*_6_) δ 9.12 (s, 2H, 2 x N*H*), 7.88 (d, J = 8.9 Hz, 4H, 2 x Ar*H*), 7.60 - 7.54 (m, 6H, 6 x Ar*H*), 7.37 (dd, J = 6.2, 3.4 Hz, 2H, 2 x Ar*H*). The spectral data are in accordance with those reported in the literature [3, 4].

N-(2,3-bis((3,4-dichlorophenyl)amino)quinoxalin-6-yl)hexanamide (**22d**)

Brown powder, 66% yield. ^1^H NMR (500 MHz, DMSO-*d*_6_) δ 10.04 (s, 1H, N*H*-C=O), 9.24 (s, 1H, N*H*), 9.17 (s, 1H¸ N*H*), 8.33 (d, *J* = 2.6 Hz, 1H, Ar*H*), 8.25 (d, *J* = 2.5 Hz, 1H, Ar*H*), 8.05 (d, *J* = 2.2 Hz, 1H, Ar*H*), 7.82 (ddd, *J* = 13.8, 8.9, 2.5 Hz, 2H, 2 x Ar*H*), 7.63 (dd, *J* = 18.7, 8.8 Hz, 2H, 2 x Ar*H*), 7.59 - 7.51 (m, 2H, 2 x Ar*H*), 2.34 (t, *J* = 7.4 Hz, 2H, C*H*_2_), 1.62 (p, *J* = 7.4 Hz, 2H, C*H*_2_), 1.32 ((td, *J* = 3.8, 1.7 Hz, 4H, 2 x C*H*_2_), 0.92 – 0.85 (t, 3H, C*H*_3_); ^13^C NMR (125 MHz, DMSO) δ 171.39 (*C*=O), 141.06 (Ar*C*), 140.64 (Ar*C*), 140.44 (Ar*C*), 139.73 (Ar*C*), 137.48 (Ar*C*), 136.28 (Ar*C*), 132.13 (Ar*C*), 130.80 (Ar*C*), 130.43 (Ar*C*H), 130.39 (Ar*C*H), 125.73 (Ar*C*H), 123.50 (Ar*C*), 123.20 (Ar*C*), 120.98 (Ar*C*H), 120.76 (Ar*C*H), 119.99 (Ar*C*H), 119.78 (Ar*C*H), 118.84 (Ar*C*H), 114.01 (Ar*C*H), 36.49 (*C*H_2_), 30.95 (*C*H_2_), 24.84 (*C*H_2_), 21.96 (*C*H_2_), 13.90 (*C*H_3_); UPLC-MS: Rt: 2.43 min, MS (ESI)^+^: 564.08 [M+H]^+^.

N-(2,3-bis((3,4-dichlorophenyl)amino)quinoxalin-6-yl)cyclohexanecarboxamide (**22e**)

Pale-yellow powder, 55% yield. ^1^H NMR (500 MHz, DMSO-*d*_6_) δ 9.98 (s, 1H, N*H*-C=O), 9.21 (s, 1H, N*H*), 9.14 (s, 1H, N*H*), 8.33 (d, *J* = 2.6 Hz, 1H, Ar*H*), 8.24 (d, *J* = 2.6 Hz, 1H, Ar*H*), 8.06 (d, *J* = 2.3 Hz, 1H, Ar*H*), 7.81 (ddd, *J* = 13.5, 8.8, 2.6 Hz, 2H, 2 x Ar*H*), 7.67 – 7.50 (m, 4H, 4 x Ar*H*), 2.36 (tt, *J* = 11.8, 3.6 Hz, 1H, C*H*), 1.87 – 1.71 (m, 5H, 2 x C*H*_2_ and C*H*), 1.44 (qd, *J* = 12.3, 3.2 Hz, 2H, C*H*_2_), 1.37 – 1.26 (m, 3H, C*H*_2_ and C*H*); ^13^C NMR (125 MHz, DMSO) δ 174.40 (*C*=O), 141.05 (Ar*C*), 140.64 (Ar*C*), 140.44 (Ar*C*), 139.73 (Ar*C*), 137.61 (Ar*C*), 136.26 (Ar*C*), 132.08 (Ar*C*), 130.78 (2xAr*C*), 130.43 (Ar*C*H), 130.40 (Ar*C*H), 125.68 (Ar*C*H), 123.46 (Ar*C*), 123.16 (Ar*C*), 120.94 (Ar*C*H), 120.75 (Ar*C*H), 119.97 (Ar*C*H), 119.77 (Ar*C*H), 118.92 (Ar*C*H), 114.05 (Ar*C*H), 44.96 (*C*H), 29.17 (*C*H_2_), 28.66 (*C*H_2_), 25.43 (*C*H_2_), 25.26 (*C*H_2_), 24.92 (*C*H_2_); UPLC-MS: Rt: 2.46 min, MS (ESI)^+^: 576.09 [M+H]^+^.

N-(2,3-bis((3,4-dichlorophenyl)amino)quinoxalin-6-yl)furan-2-carboxamide (**22f**)

Yellow powder, 45% yield. ^1^H NMR (500 MHz, DMSO-*d*_6_) δ 10.35 (s, 1H, N*H*-C=O), 9.26 (s, 1H, N*H*), 9.20 (s, 1H, N*H*), 8.35 (d, *J* = 2.5 Hz, 1H, Ar*H*), 8.26 (d, *J* = 2.5 Hz, 1H, Ar*H*), 8.14 (d, *J* = 2.3 Hz, 1H, Ar*H*), 7.96 (dd, *J* = 1.8, 0.8 Hz, 1H, Ar*H*), 7.83 (dt, *J* = 8.9, 2.0 Hz, 3H, 3 x Ar*H*), 7.69 – 7.55 (m, 3H, 3 x Ar*H*), 7.39 (dd, *J* = 3.5, 0.9 Hz, 1H, Ar*H*), 6.72 (dd, *J* = 3.5, 1.7 Hz, 1H, Ar*H*); ^13^C NMR (125 MHz, DMSO) δ 156.61 (*C*=O), 147.88 (Ar*C*), 146.23 (Ar*C*H), 141.57 (Ar*C*), 140.97 (Ar*C*), 140.80 (Ar*C*), 140.45 (Ar*C*), 137.07 (Ar*C*), 136.55 (Ar*C*), 133.03 (Ar*C*), 131.20 (Ar*C*), 130.85(Ar*C*H), 130.83 (Ar*C*H), 126.07 (Ar*C*H), 123.95 (Ar*C*), 123.70 (Ar*C*), 121.45 (Ar*C*H), 121.26(Ar*C*H), 120.47 (Ar*C*H), 120.28 (Ar*C*H), 120.18(Ar*C*H), 115.79(Ar*C*H), 115.20 (Ar*C*H), 112.59 (Ar*C*H); UPLC-MS: Rt: 2.30 min, MS (ESI)^+^: 559.93 [M]^+^.

N-(2,3-bis((3,4-dichlorophenyl)amino)quinoxalin-6-yl)-3-(trifluoromethyl)benzamide (**22g**)

Yellow powder, 95% yield. ^1^H NMR (500 MHz, DMSO-*d*_6_) δ 10.62 (s, 1H, N*H*-C=O), 9.28 (s, 1H, N*H*), 9.22 (s, 1H, N*H*), 8.36 (d, *J* = 2.6 Hz, 2H, 2 x Ar*H*), 8.31 (d, *J* = 7.9 Hz, 1H, Ar*H*), 8.28 (d, *J* = 2.5 Hz, 1H, Ar*H*), 8.23 (d, *J* = 7.8 Hz, 1H, Ar*H*), 8.17 (d, *J* = 2.4 Hz, 1H, Ar*H*), 7.98 (d, *J* = 7.8 Hz, 1H, Ar*H*), 7.88 – 7.81 (m, 3H, 3 x Ar*H*), 7.67 – 7.60 (m, 3H, 3 x Ar*H*); ^13^C NMR (125 MHz, DMSO) δ 163.96 (*C*=O), 141.16 (Ar*C*), 140.54 (Ar*C*), 140.39 (Ar*C*), 140.13 (Ar*C*), 136.89 (Ar*C*), 136.12 (Ar*C*), 135.70 (Ar*C*), 133.23 (Ar*C*H), 132.79 (Ar*C*), 131.88 (Ar*C*H), 130.80 (Ar*C*), 130.42 (Ar*C*H), 130.10 (Ar*C*H), 129.76 (Ar*C*H), 125.70 (Ar*C*H), 124.29 (Ar*C*H), 123.57 (Ar*C*), 123.34 (Ar*C*), 121.03 (Ar*C*H), 120.90 (Ar*C*H), 120.06 (Ar*C*H), 119.91 (Ar*C*H), 119.84 (Ar*C*H), 115.65 (Ar*C*H); UPLC-MS: Rt: 2.44 min, MS (ESI)^+^: 638.07 [M+H]^+^.

**^1^H-NMR and ^13^C-NMR spectra**

Compound **27**

^1^H NMR (500 MHz, DMSO-d6)


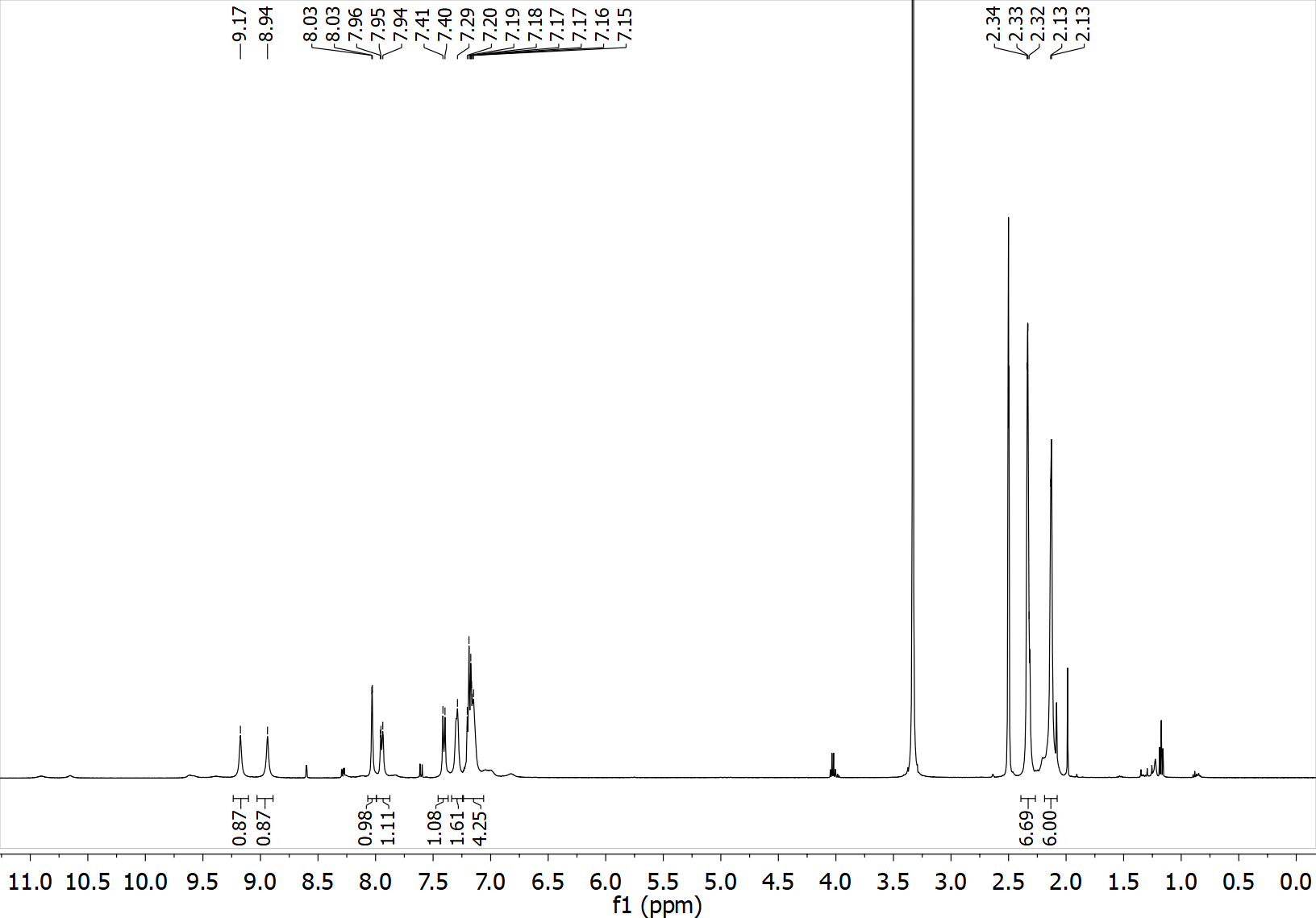


^13^C NMR (125 MHz, DMSO)

**
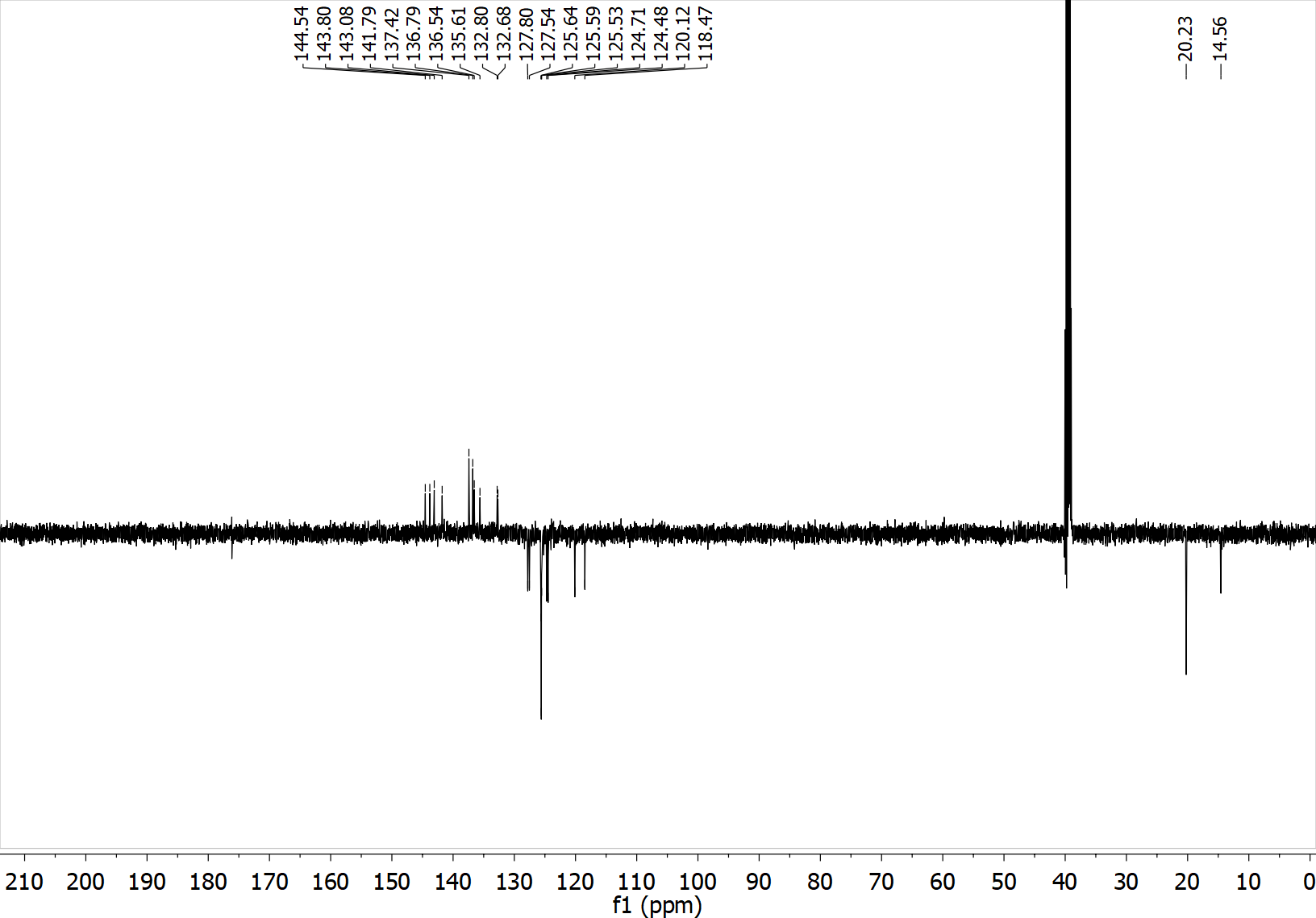
**

 Compound **32**

^1^H NMR (500 MHz, DMSO-d6)

**
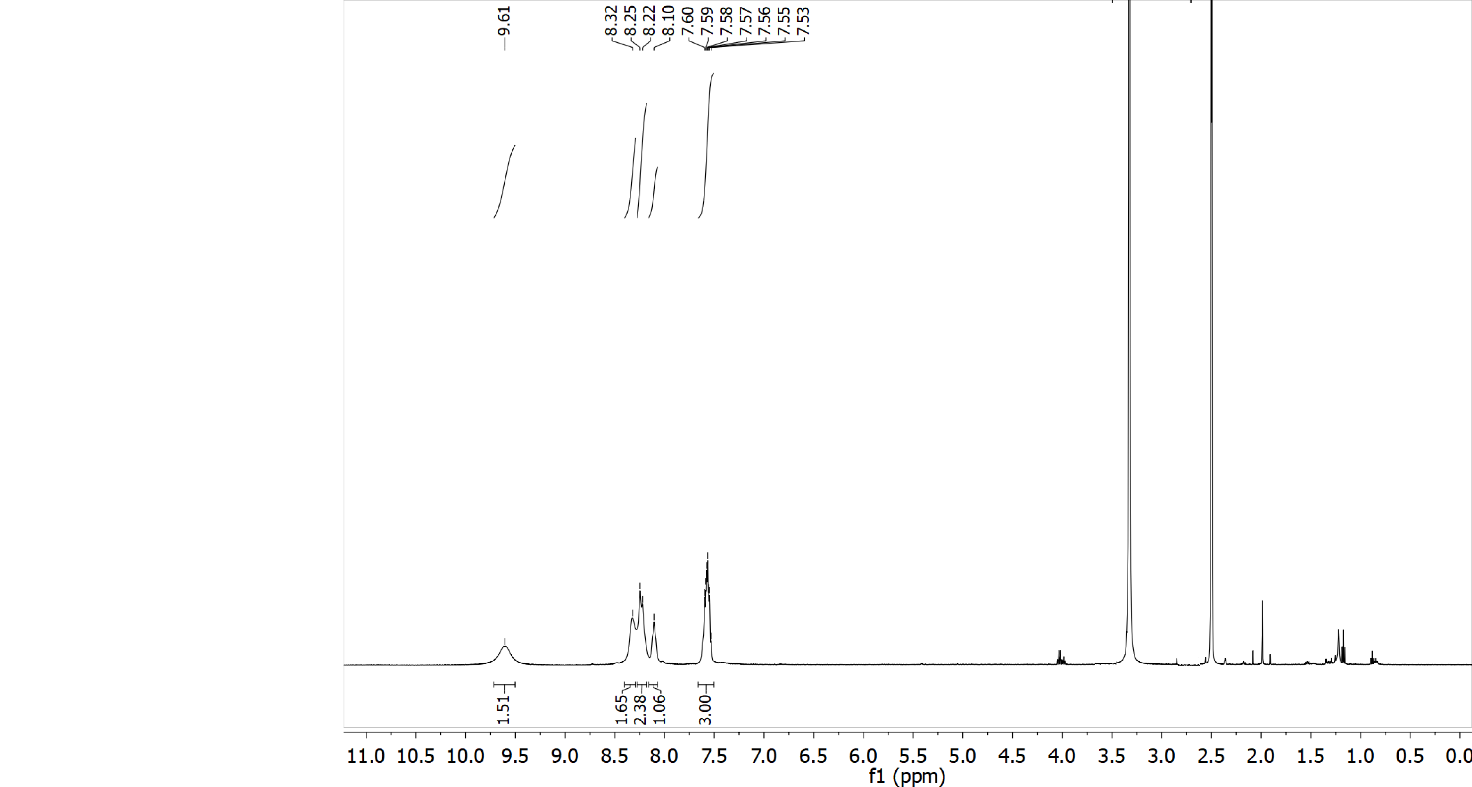
**

^13^C NMR (125 MHz, DMSO)

^
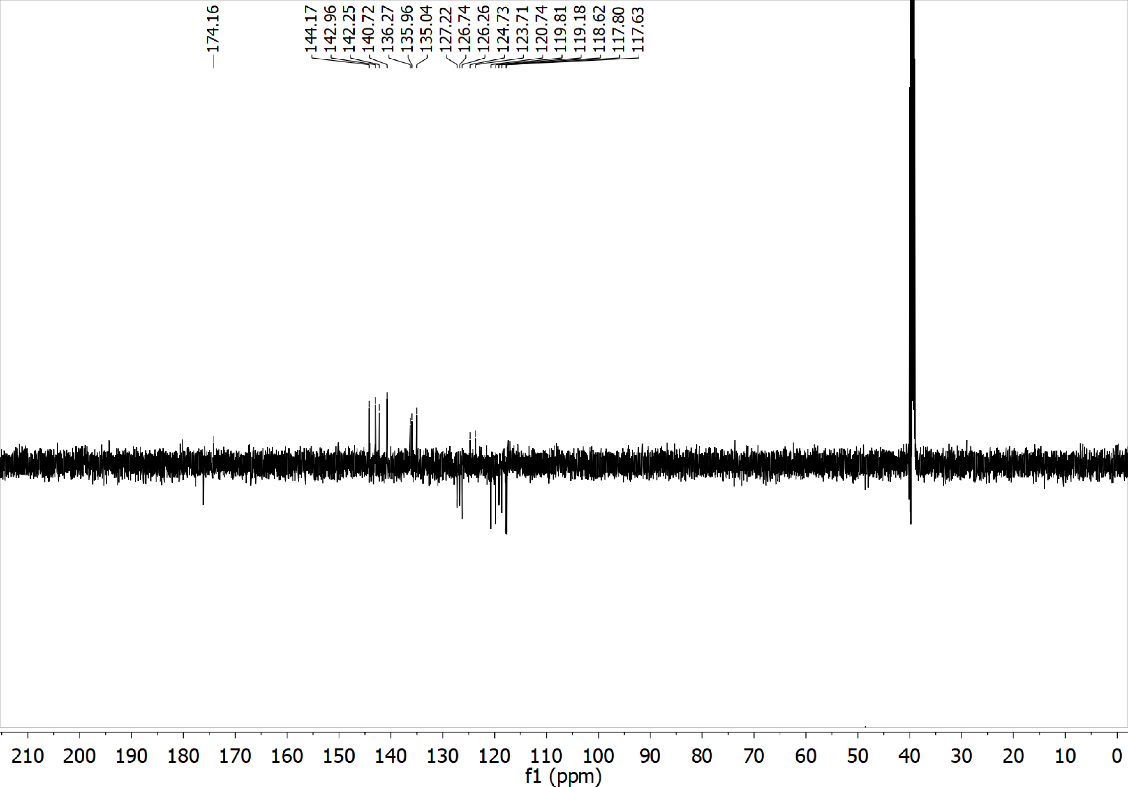
^

Compound **35**

^1^H NMR (500 MHz, DMSO-d6)


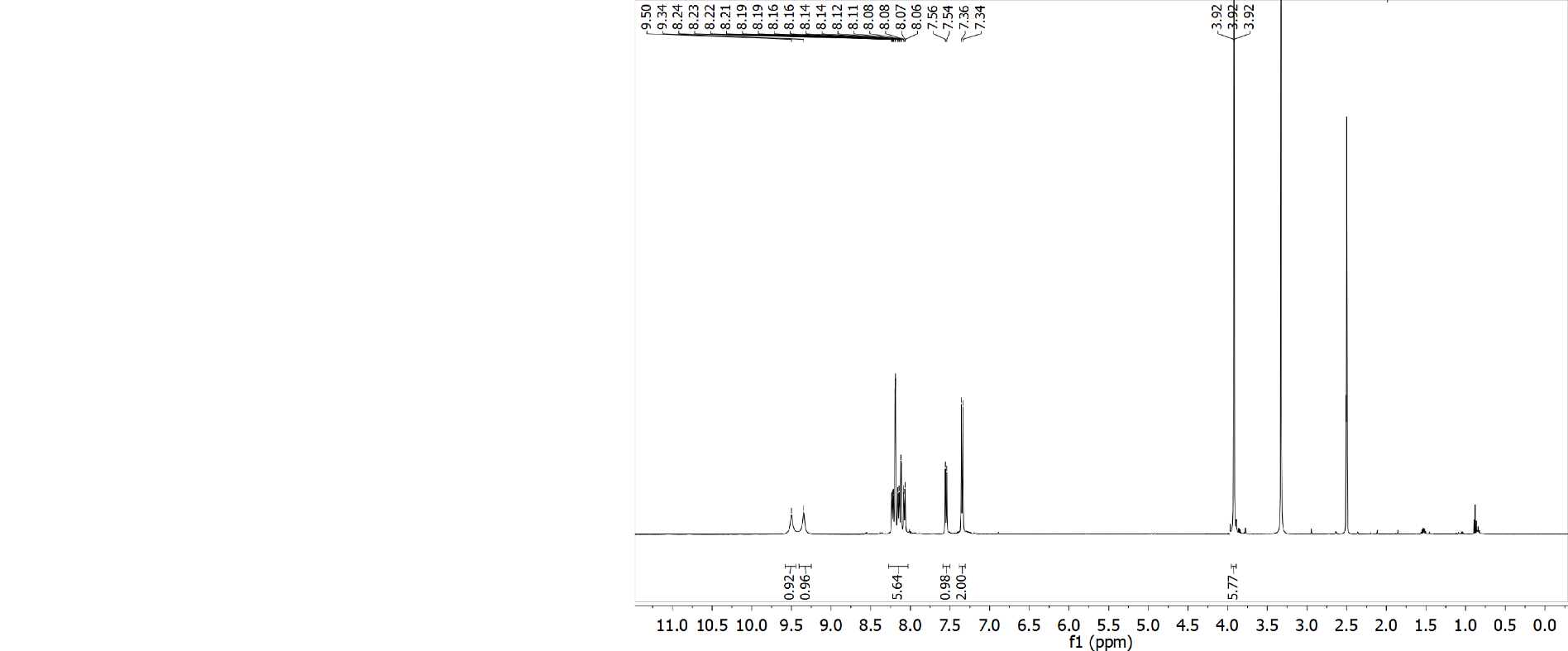


^13^C NMR (125 MHz, DMSO)


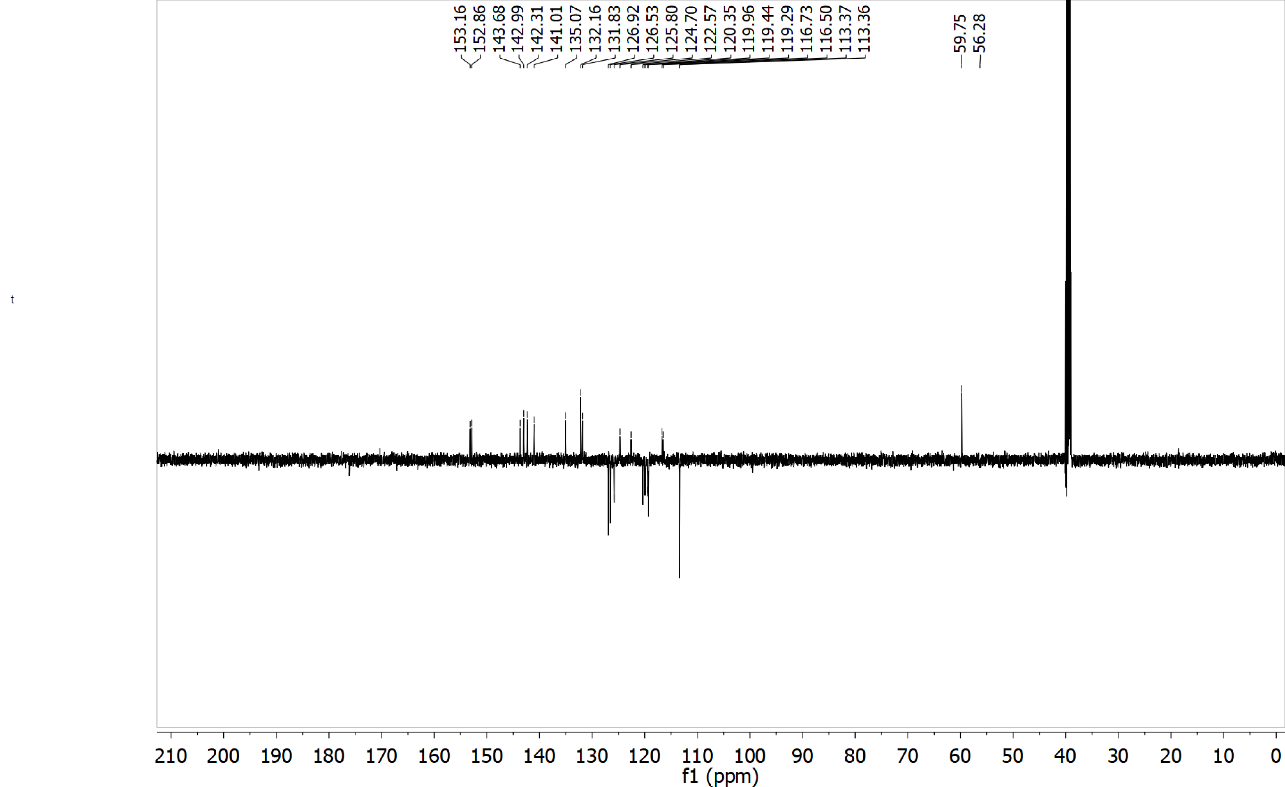

Compound **36**

^1^H NMR (500 MHz, DMSO-d6)


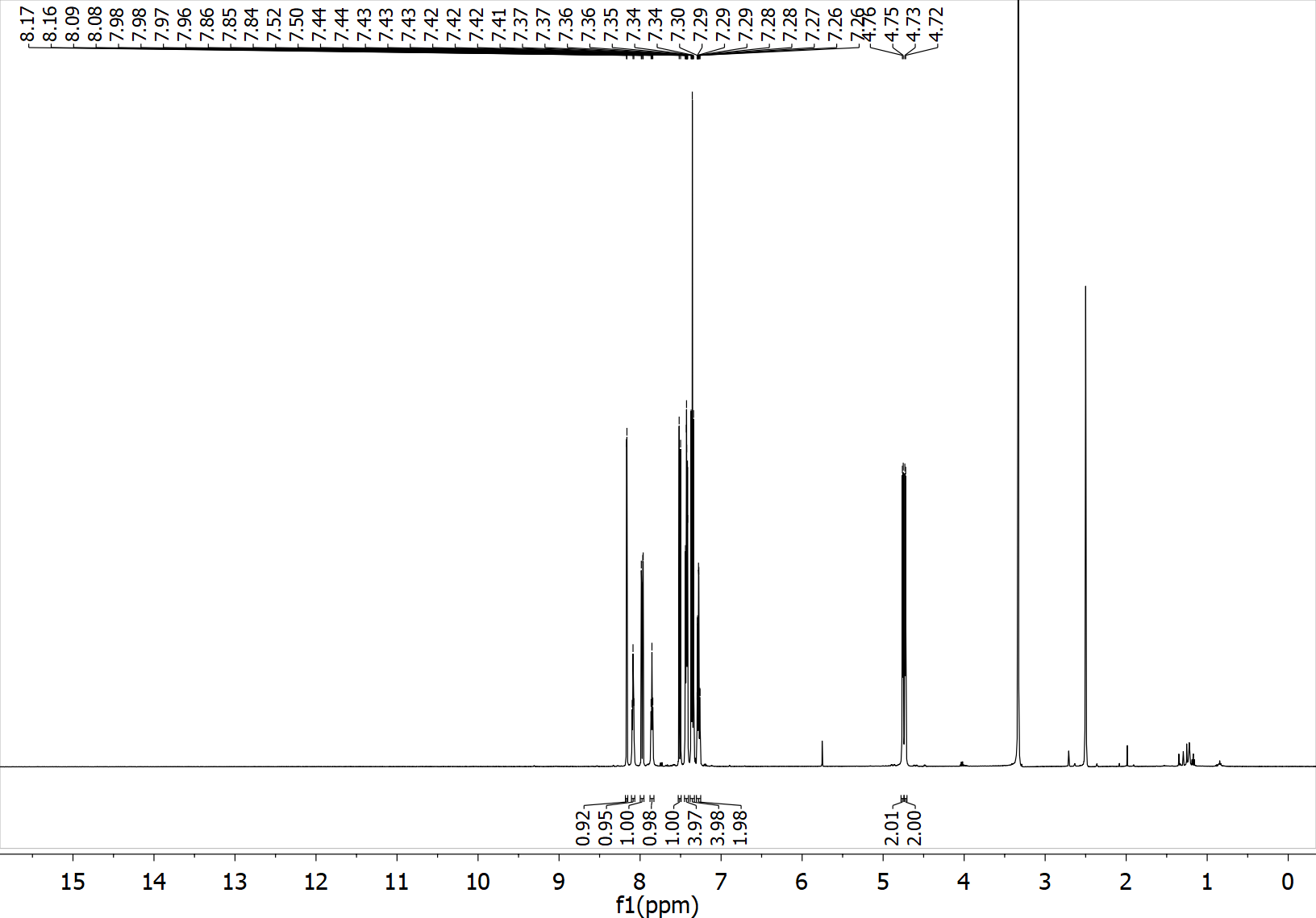


^13^C NMR (125 MHz, DMSO)


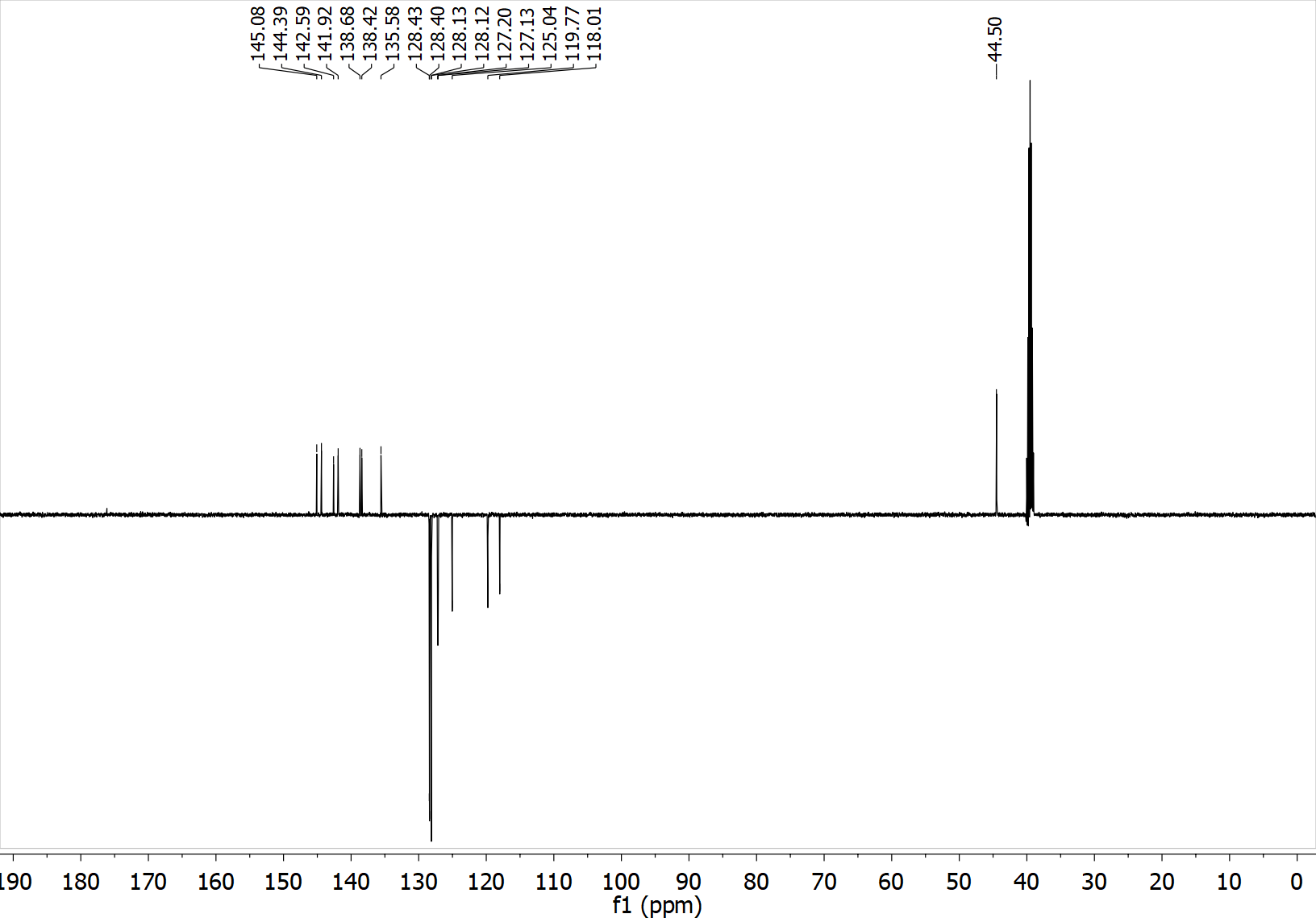

Compound **37**

^1^H NMR (500 MHz, DMSO-d6)


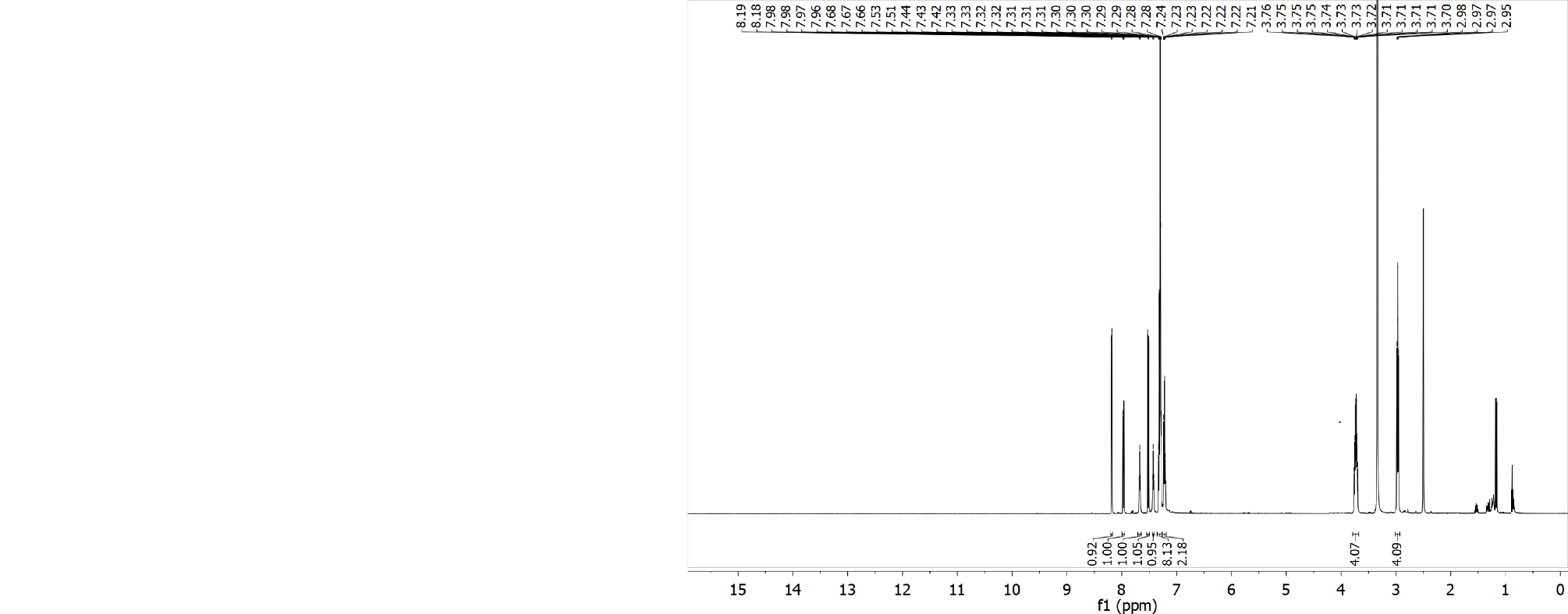


^13^C NMR (125 MHz, DMSO)


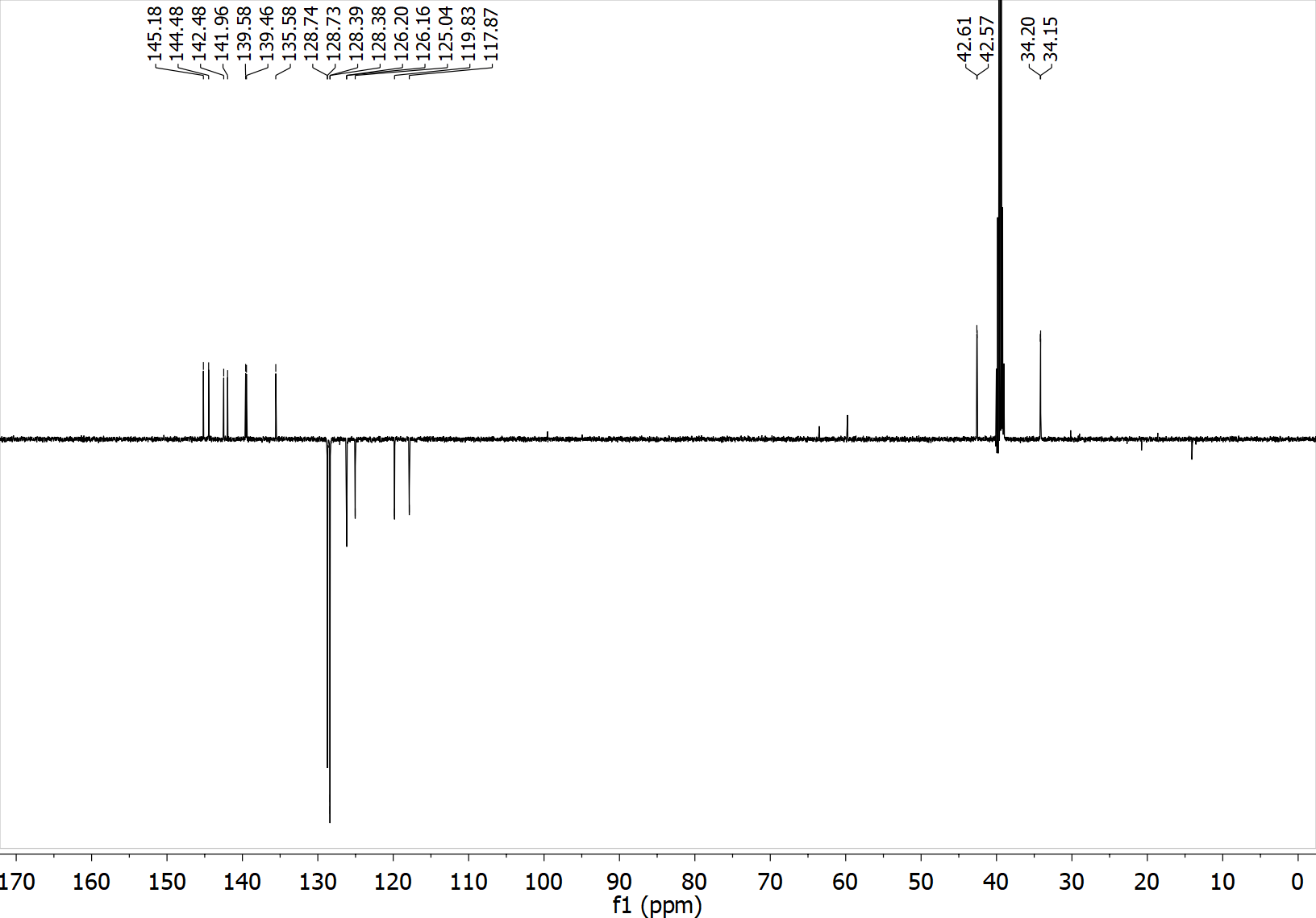

Compound **22a**

^1^H NMR (500 MHz, DMSO-d6)


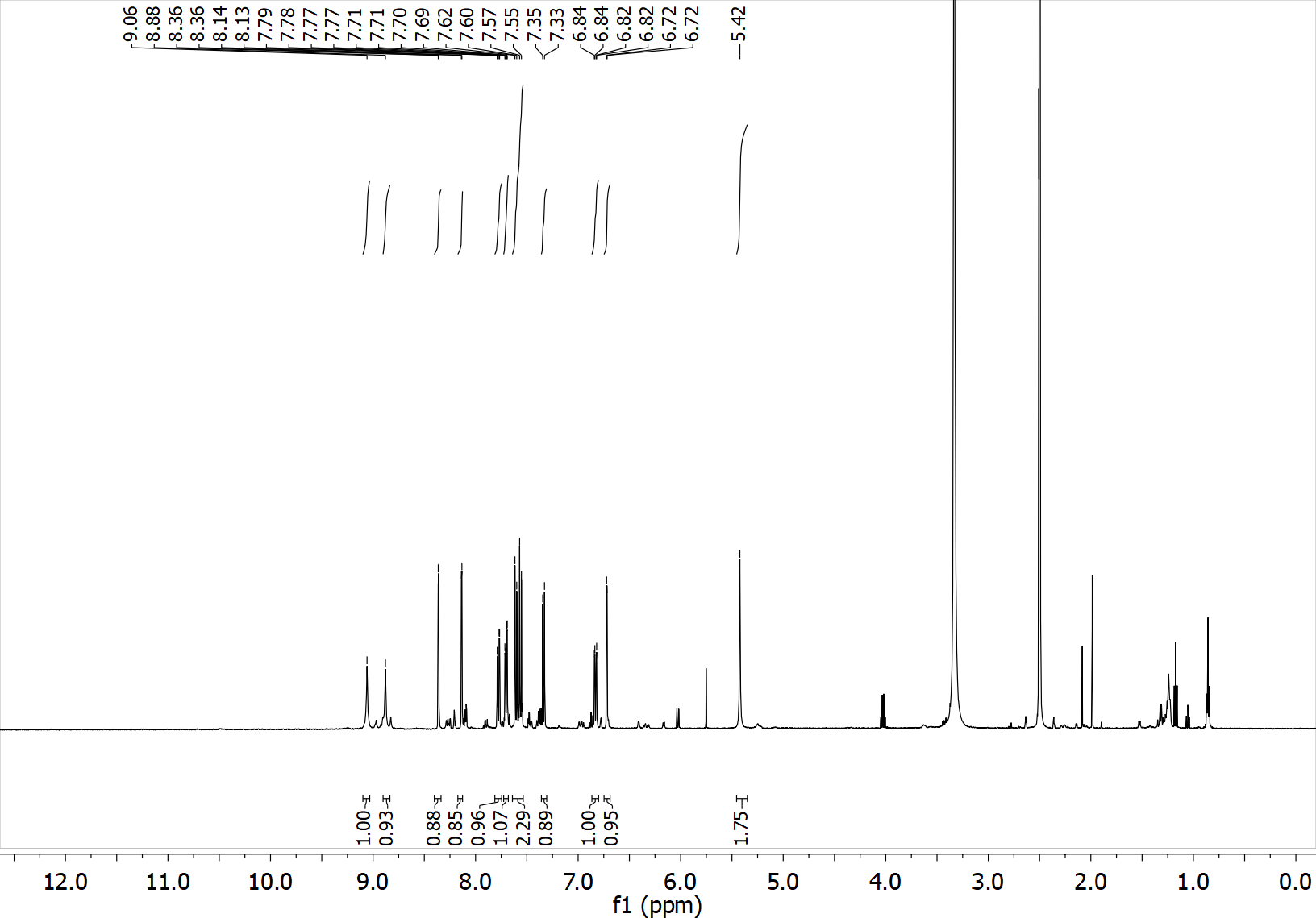


^13^C NMR (125 MHz, DMSO)


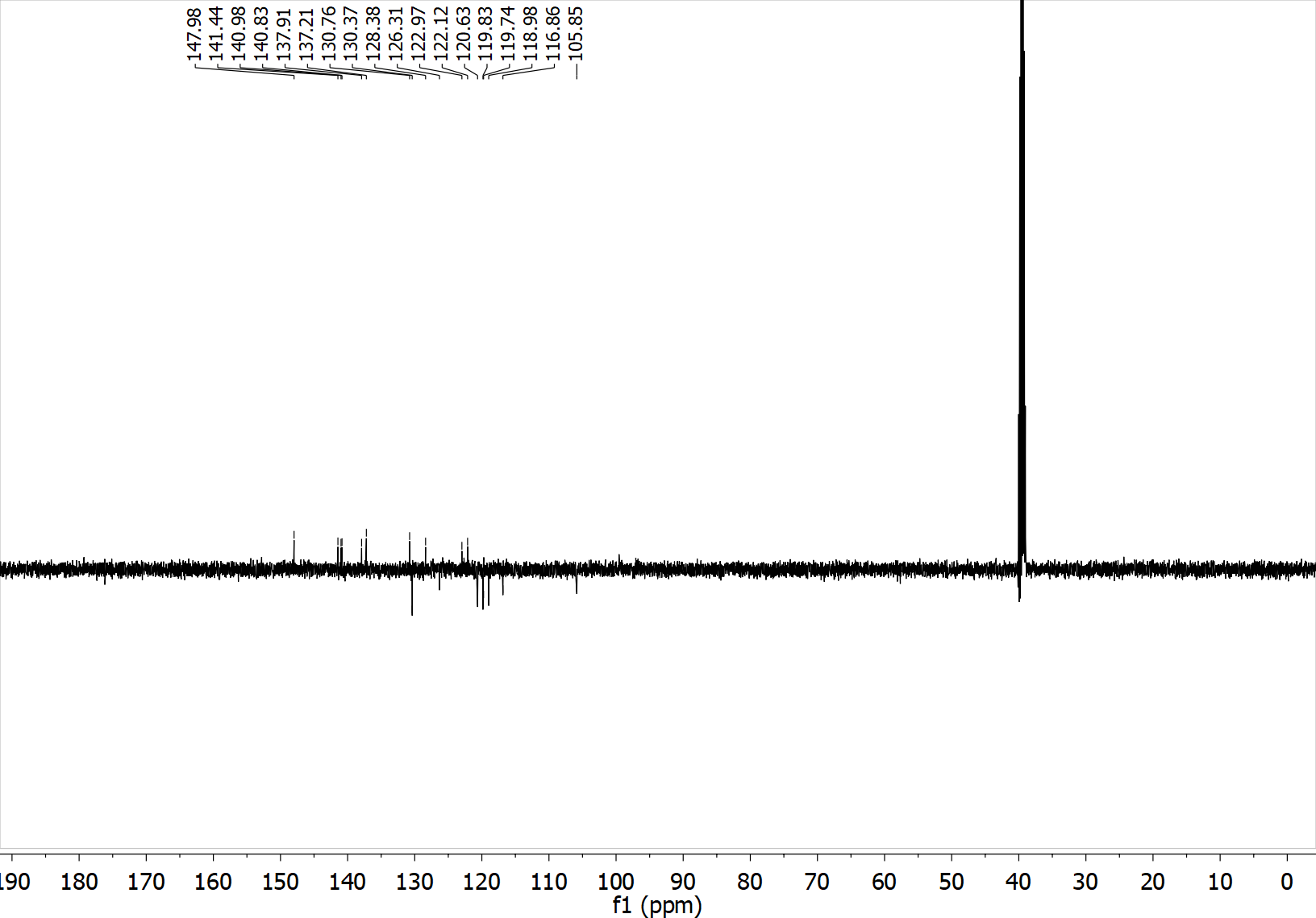

Compound **22b**

^1^H NMR (500 MHz, DMSO-d6)


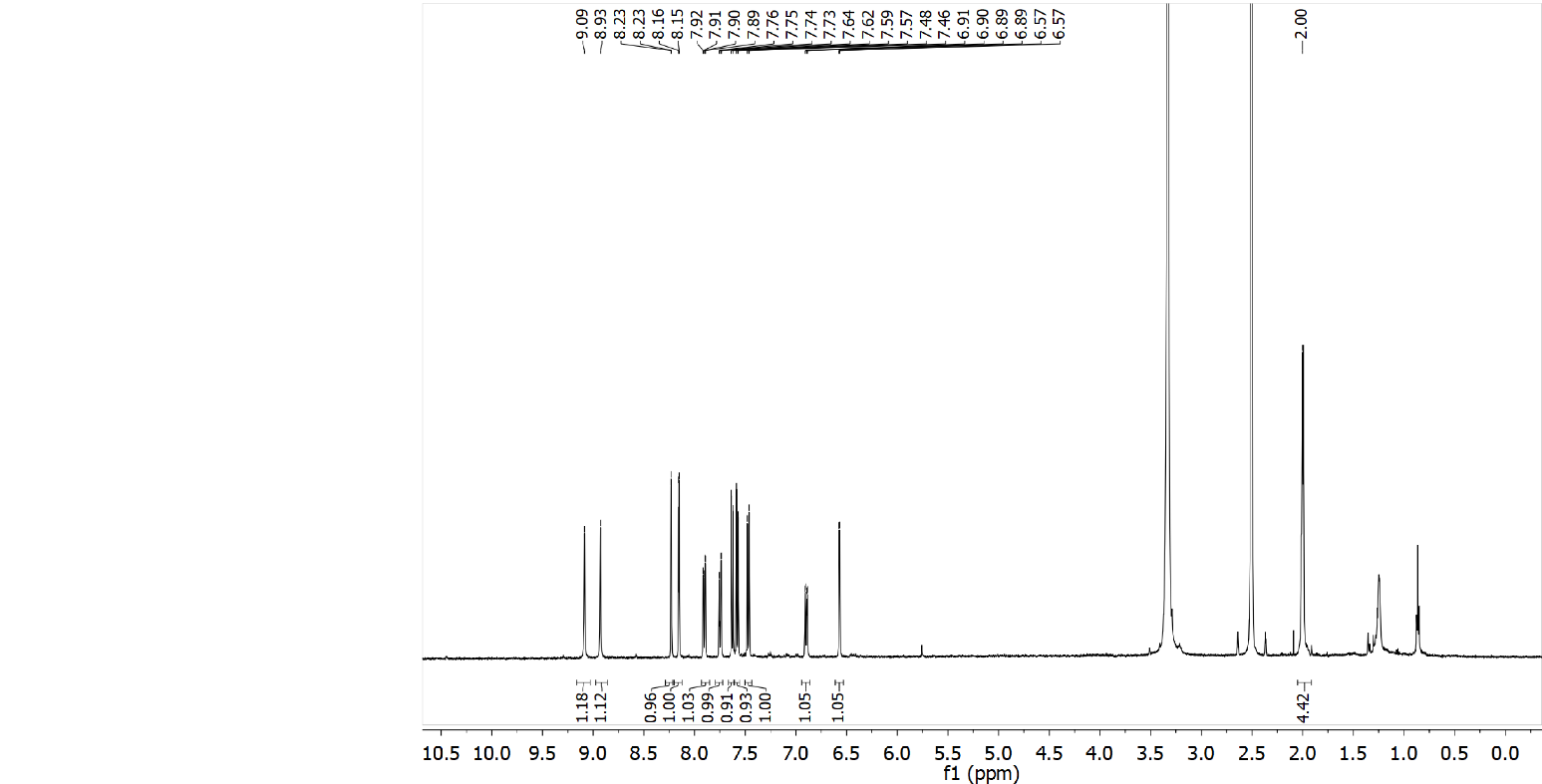


^1^H NMR (500 MHz, DMSO-d6) + TFA


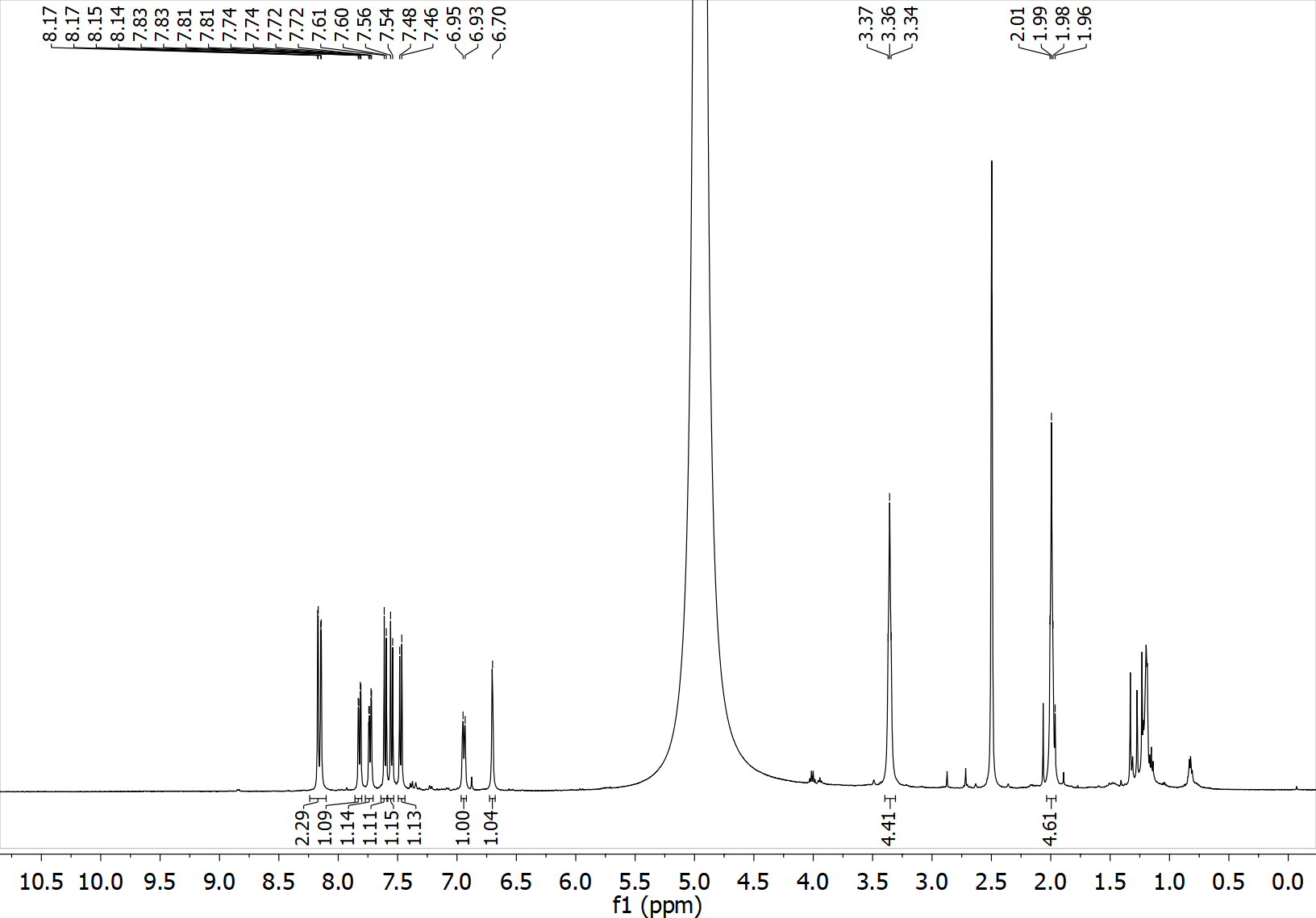


^13^C NMR (125 MHz, DMSO)


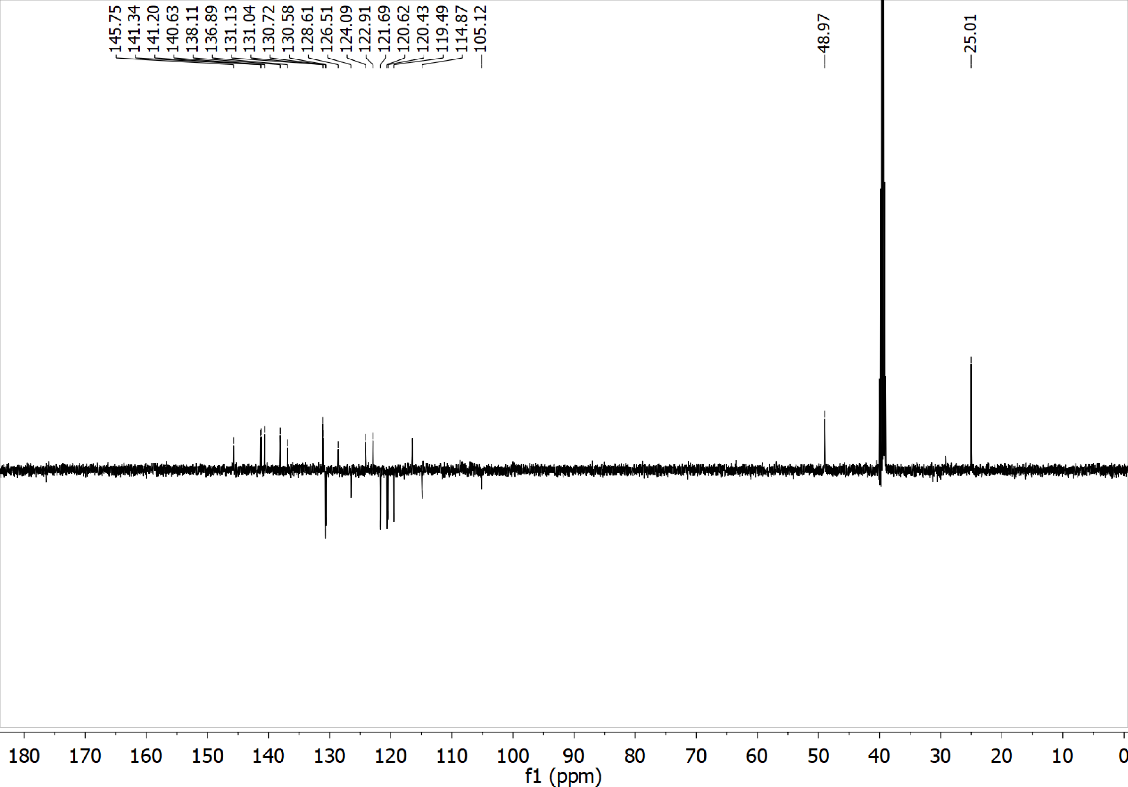

Compound **22d**

^1^H NMR (500 MHz, DMSO-d6)


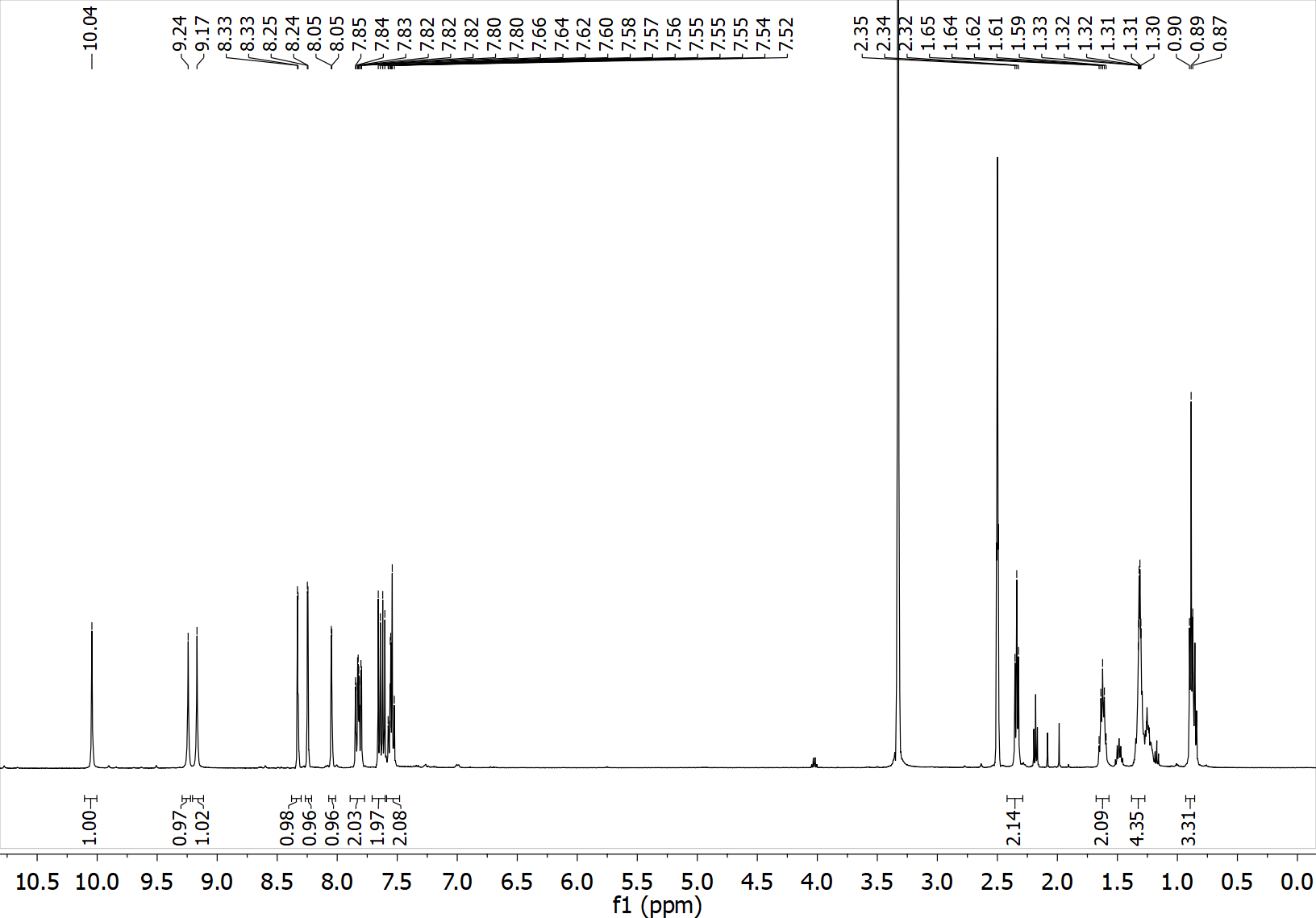


^13^C NMR (125 MHz, DMSO)


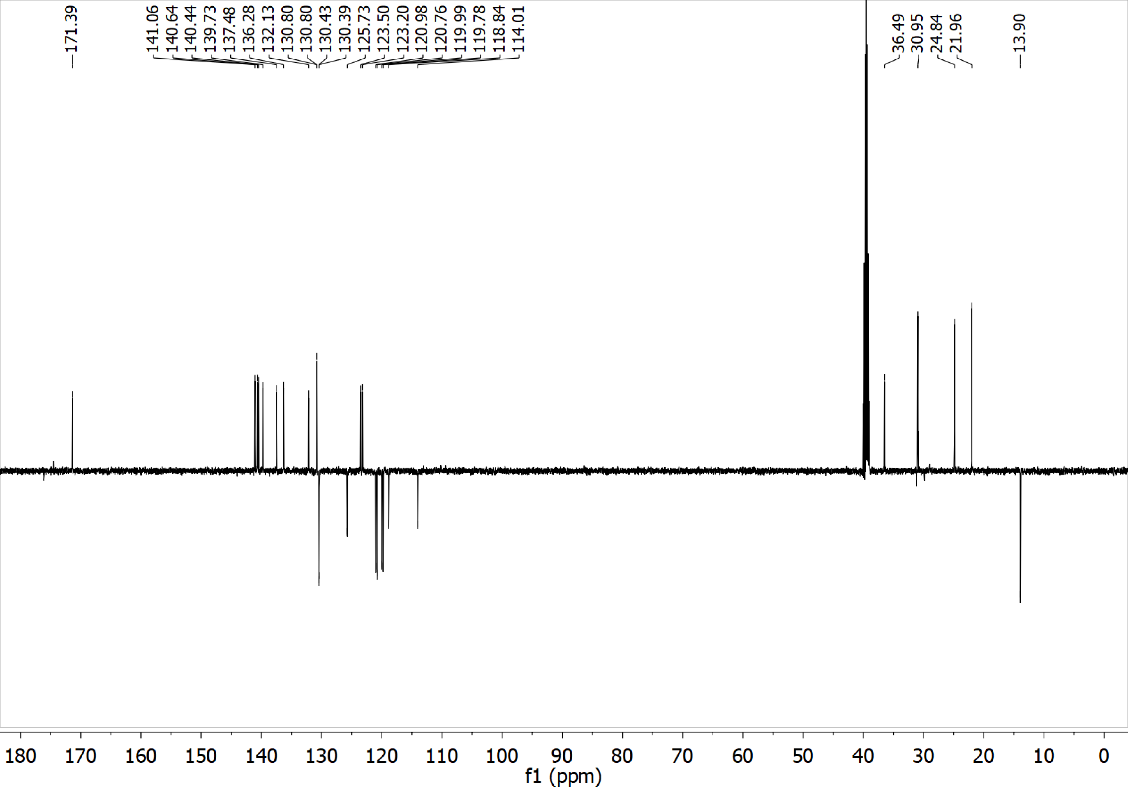

Compound **22e**

^1^H NMR (500 MHz, DMSO-d6)


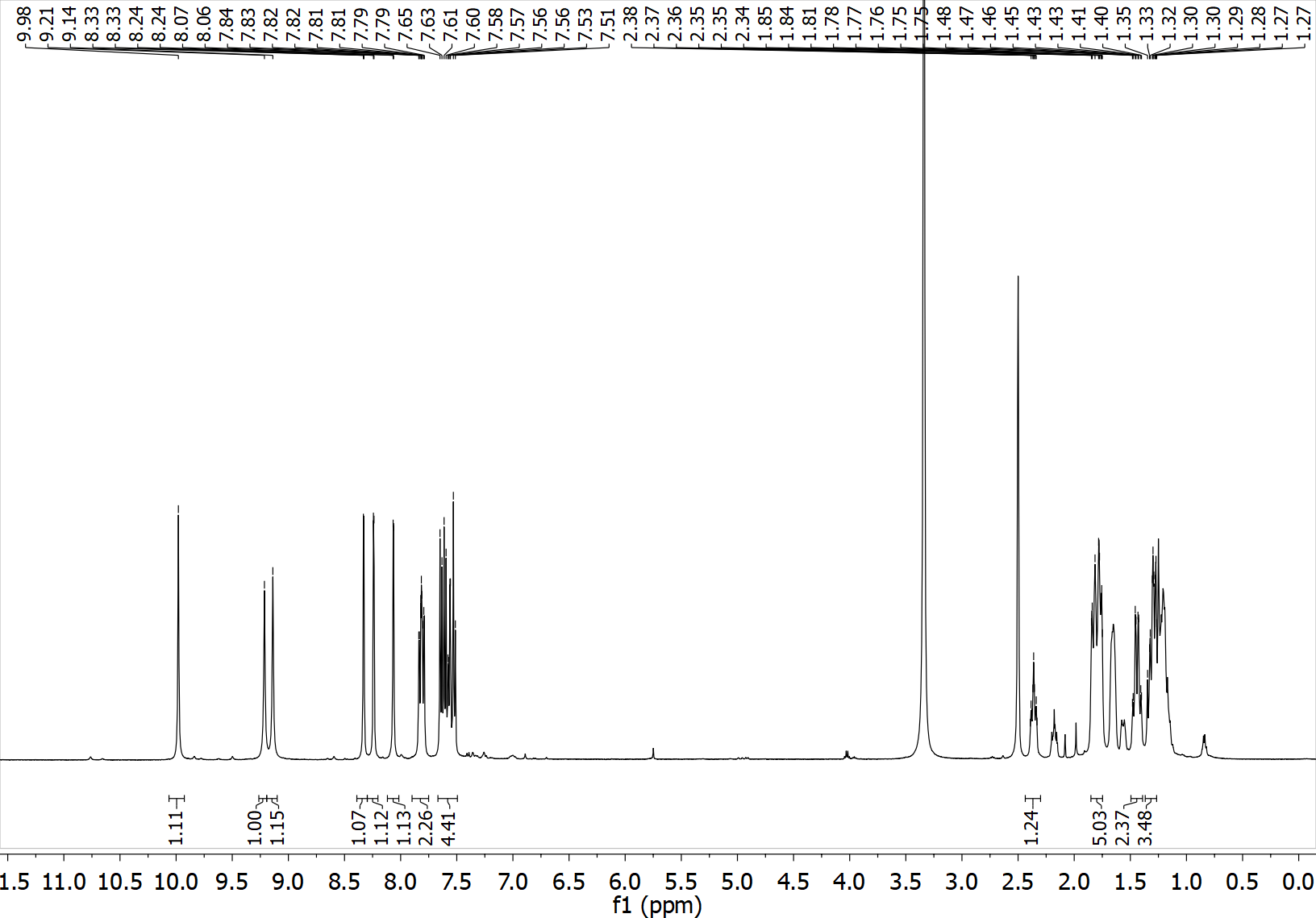


^13^C NMR (125 MHz, DMSO)


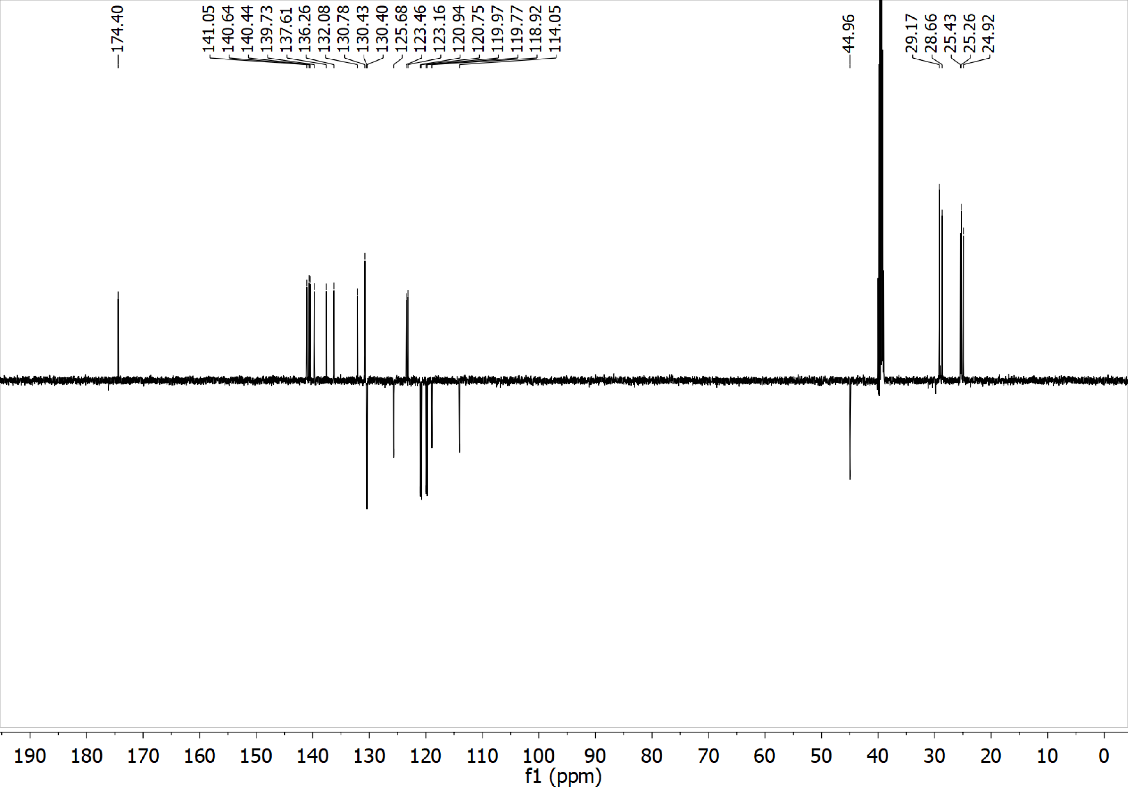

Compound **22f**

^1^H NMR (500 MHz, DMSO-d6)


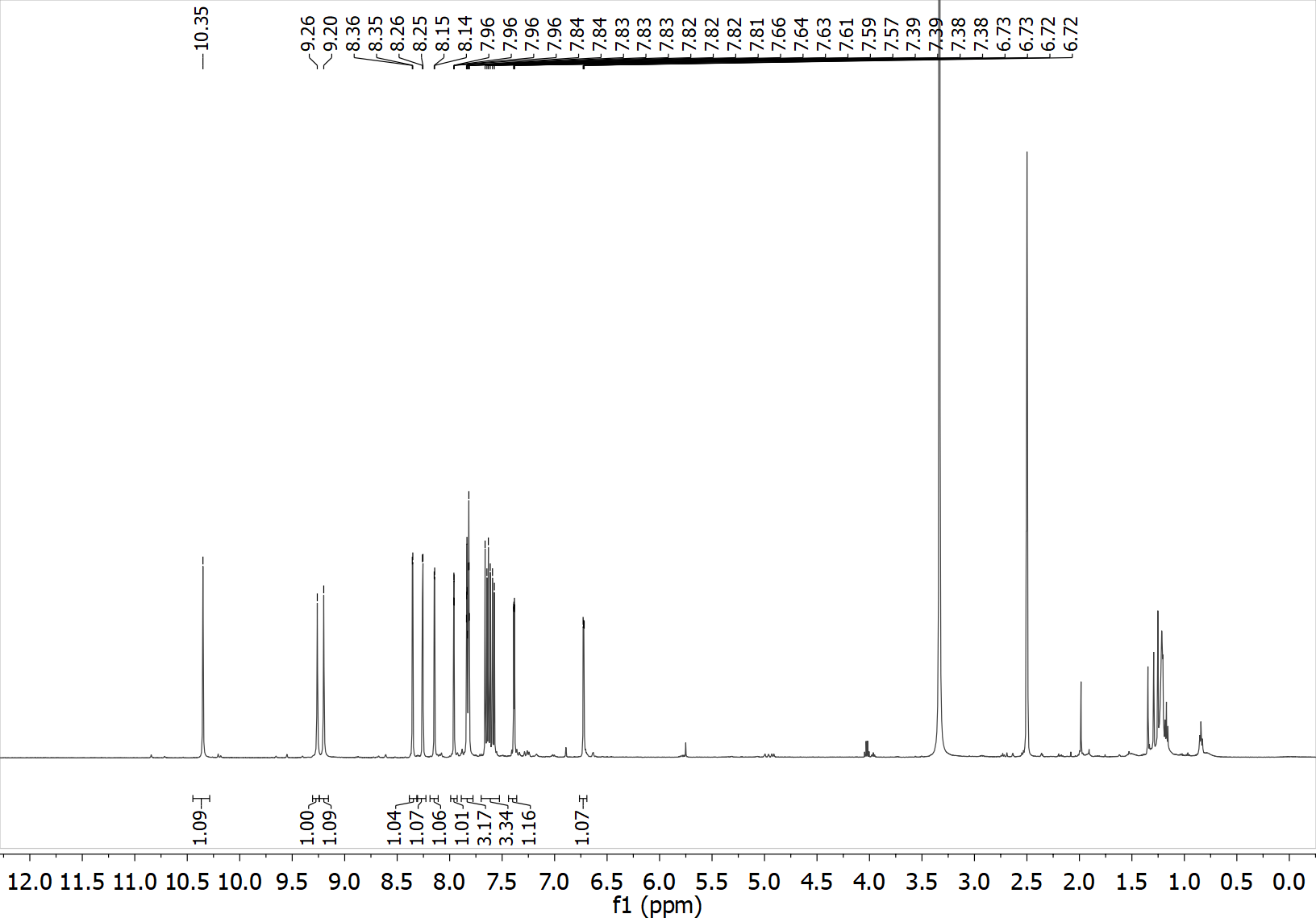


^13^C NMR (125 MHz, DMSO)


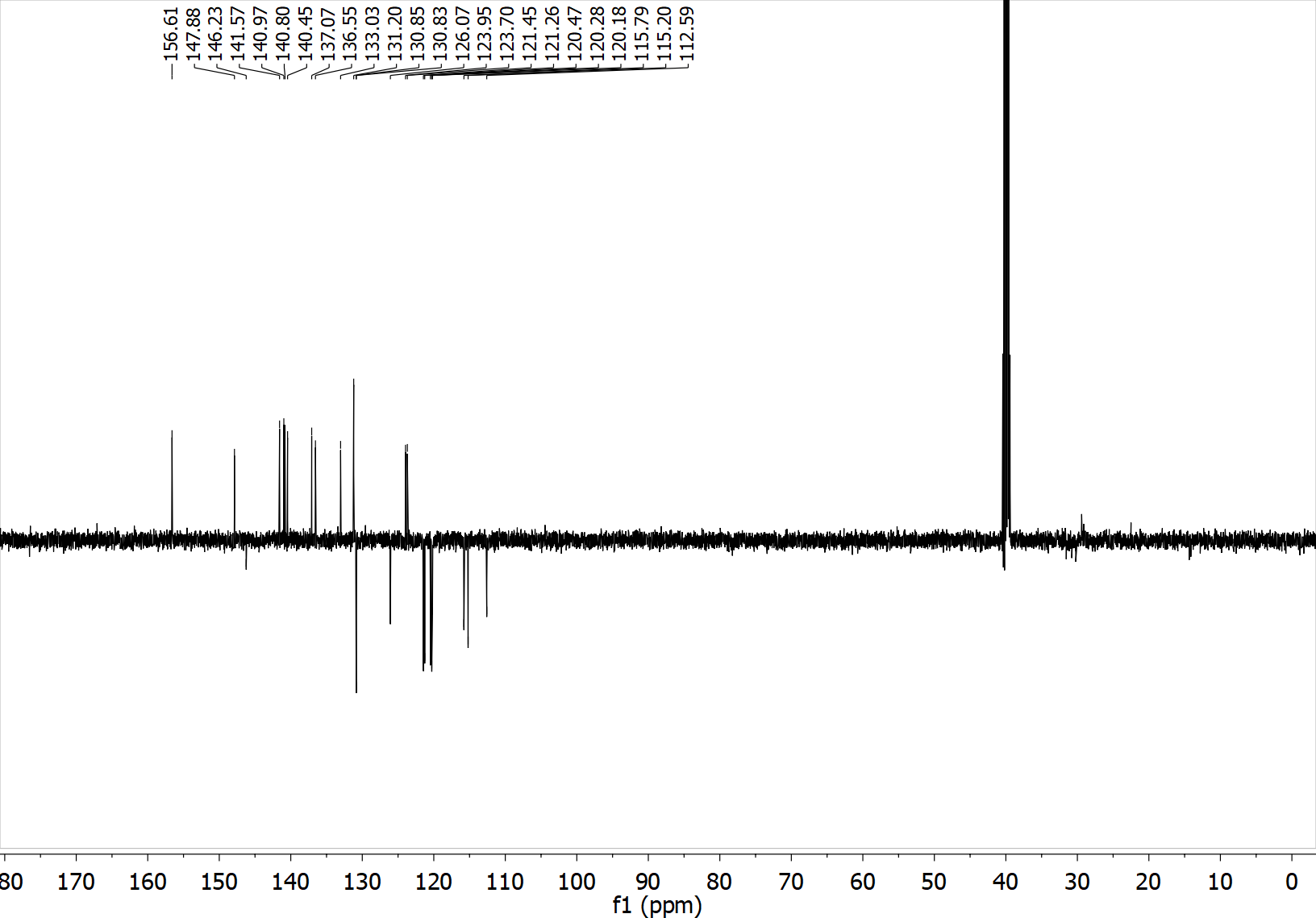

Compound **22g**

^1^H NMR (500 MHz, DMSO-d6)


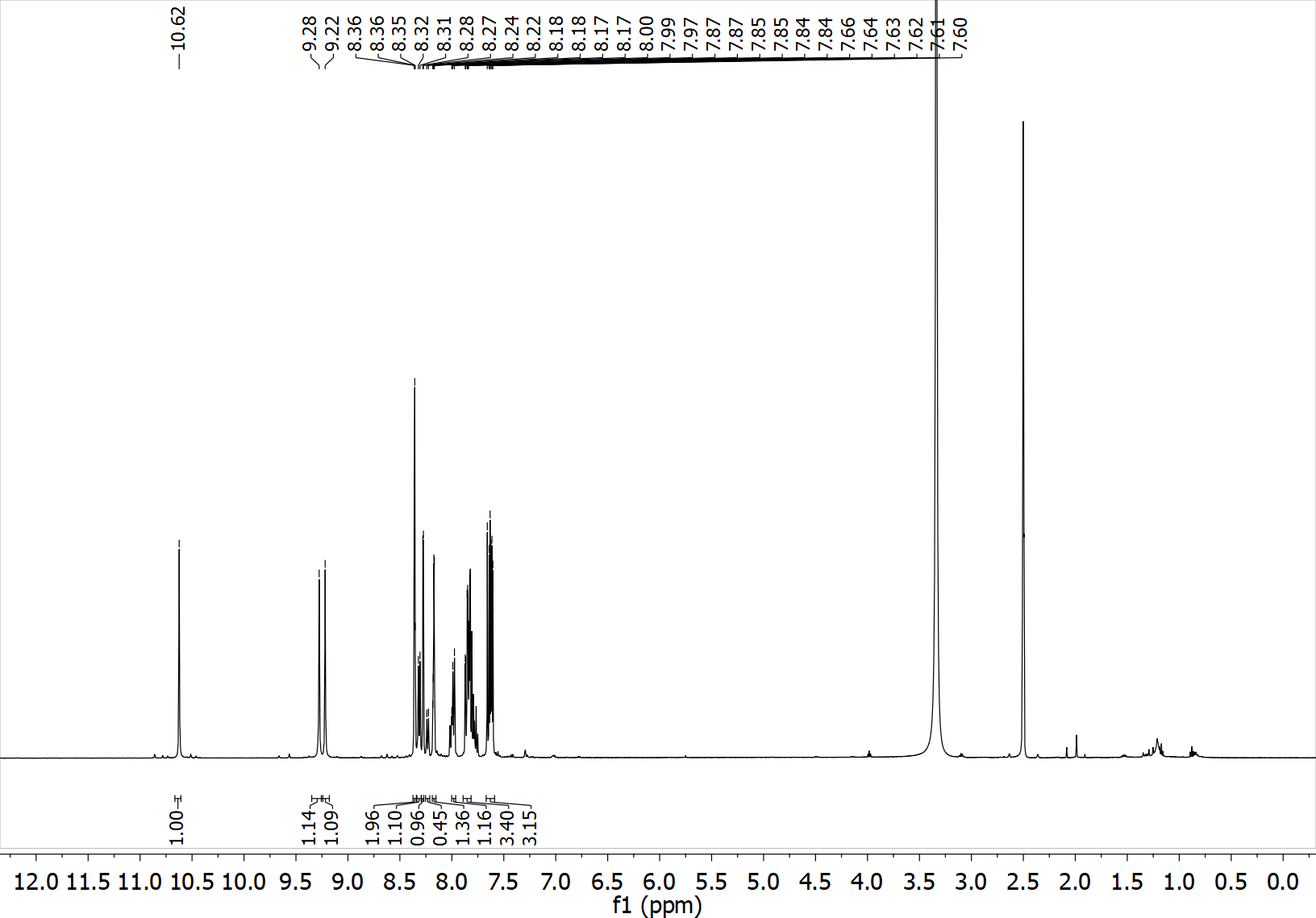


^13^C NMR (125 MHz, DMSO)


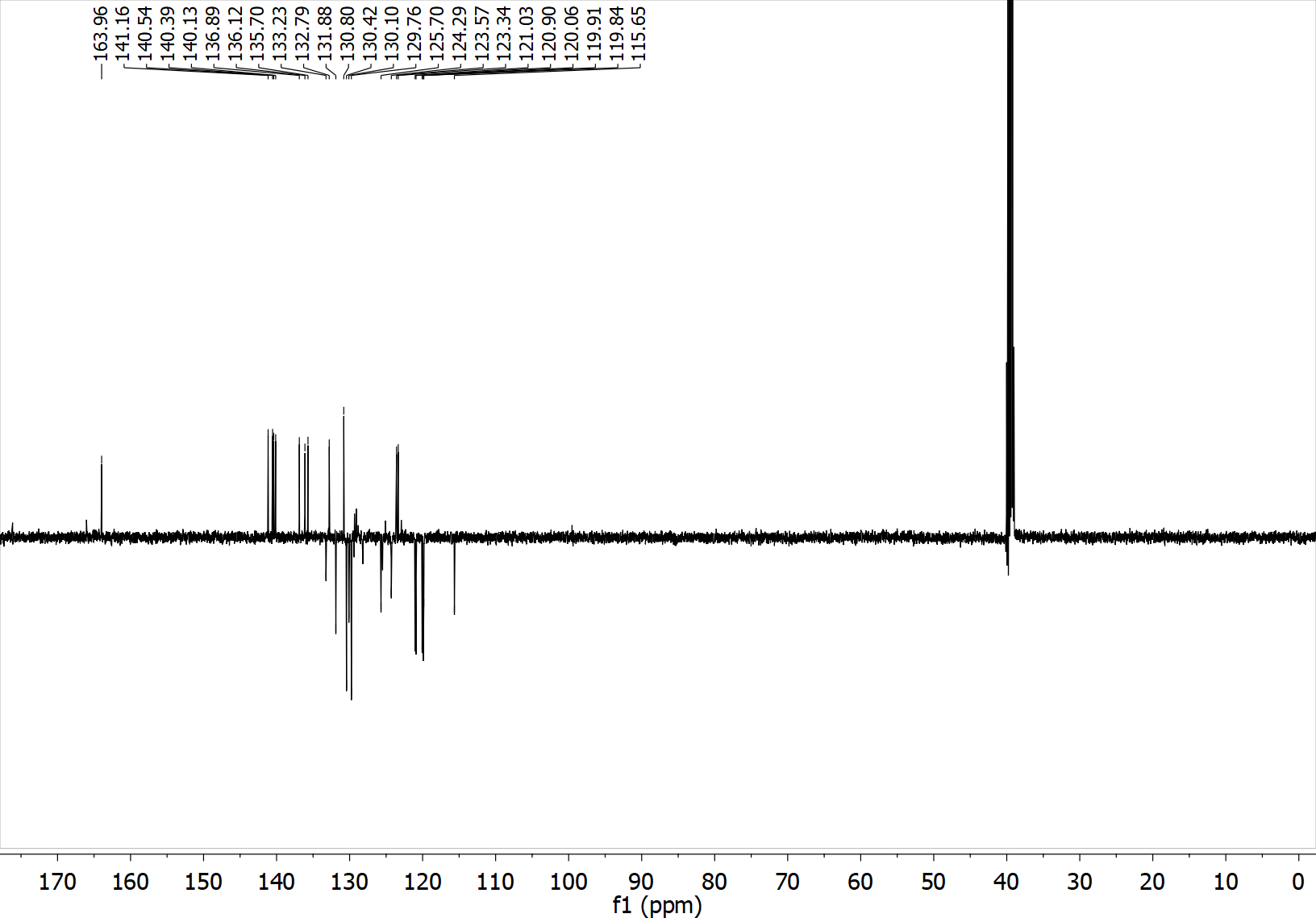


REFERENCES

[1] S.L. Debbert, M.J. Hintz, C.J. Bell, K.R. Earl, G.E. Forsythe, C. Häberli, J. Keiser, Activities of Quinoxaline, Nitroquinoxaline, and [1,2,4]Triazolo[4,3-a]quinoxaline Analogs of MMV007204 against Schistosoma mansoni, Antimicrobial Agents and Chemotherapy, 65 (2021) e01370-01320.

[2] K.-F. Chen, C.-W. Shiau, C.-H. Chen, Aryl amine substituted quinoxaline used as anticancer drugs, in, Google Patents, 2019.

[3] S.S. Kher, M. Penzo, S. Fulle, J.P. Ebejer, P.W. Finn, M.J. Blackman, A. Jirgensons, Quinoxaline-Based Inhibitors of Malarial Protease PfSUB1, Chemistry of Heterocyclic Compounds, 50 (2015) 1457-1463.

[4] J. Bosch, L.E. Boucher, Cell egress and invasion inhibitors and their use as antiparasitical agents, in, Google Patents, 2018.
